# Revision of the Malagasy genus *Trichoteleia* Kieffer (Hymenoptera, Platygastroidea, Platygastridae)

**DOI:** 10.3897/zookeys.80.907

**Published:** 2011-02-16

**Authors:** Elijah J. Talamas, Lubomír Masner, Norman F. Johnson

**Affiliations:** 1 Department of Entomology, The Ohio State University, 1315 Kinnear Road, Columbus, Ohio 43212, U.S.A.; 2 Agriculture and Agri-Food Canada, K.W. Neatby Building, Ottawa, Ontario K1A 0C6, Canada; 3 Department of Evolution, Ecology, and Organismal Biology, The Ohio State University, 1315 Kinnear Road, Columbus, Ohio 43212, U.S.A.

**Keywords:** Platygastridae, Scelionidaem, Trichoteleia, key, revision, monograph, database, endemicity, Madagascar

## Abstract

The species of the genus Trichoteleia Kieffer (Hymenoptera: Platygastridae) are revised: 42 species are recognized, of which two were previously named and are redescribed: Trichoteleia afo Talamas, **sp. n.**, Trichoteleia albidipes Kieffer, Trichoteleia bicolor Talamas, **sp. n.**; Trichoteleia bidentata Talamas **sp. n.**; Trichoteleia carinata Talamas, **sp. n.**; Trichoteleia cincta Talamas & Masner, **sp. n.**; Trichoteleia delilah Talamas, **sp. n.**; Trichoteleia eburata Talamas, **sp. n.**; Trichoteleia echinata Talamas, **sp. n.**; Trichoteleia fisheri Talamas & Masner, **sp. n.**; Trichoteleia funesta Talamas, **sp. n.**; Trichoteleia halterata Talamas & Masner, **sp. n.**; Trichoteleia hemlyae Talamas & Masner, **sp. n.**; Trichoteleia irwini Talamas & Masner, **sp. n.**; Trichoteleia janus Talamas, **sp. n.**; Trichoteleia jiro Talamas, **sp. n.**; T. *ketrona* Talamas, **sp. n.**; Trichoteleia levii Talamas & Johnson, **sp. n.**; Trichoteleia longiventris Talamas & Masner, **sp. n.**; Trichoteleia minima Talamas, **sp. n.**; Trichoteleia nify Talamas & Masner, **sp. n.**; Trichoteleia oculea Talamas, **sp. n.**; Trichoteleia orona Talamas & Masner, **sp. n.**; Trichoteleia parvipennis Talamas & Masner, **sp. n.**; Trichoteleia pauliani (Risbec); Trichoteleia picturata Talamas, **sp. n.**; Trichoteleia prima Talamas, **sp. n.**; Trichoteleia prolixa Talamas, **sp. n.**; Trichoteleia quazii Talamas, **sp. n.**; Trichoteleia ravaka Talamas, **sp. n.**; Trichoteleia rugifrons Talamas & Masner, **sp. n.**; Trichoteleia solocis Talamas, **sp. n.**; Trichoteleia sphaerica Talamas, **sp. n.**; Trichoteleia subtilis Talamas & Masner, **sp. n.**; Trichoteleia tahotra Talamas & Masner, **sp. n.**; Trichoteleia takariva Talamas, **sp. n.**; Trichoteleia tezitra Talamas, **sp. n.**; Trichoteleia tigris Talamas, **sp. n.**; Trichoteleia tonsa Talamas, **sp. n.**; Trichoteleia warreni Talamas & Masner, **sp. n.**; Trichoteleia xantrox Talamas, **sp. n.**; Trichoteleia zuparkoi Talamas & Masner, **sp. n.** A neotype is designated for Trichoteleia albidipes and a lectotype is designated for Trichoteleia pauliani.

## Introduction

The richness and endemicity of Madagascar’s fauna and flora is well documented in a wide variety of organisms. The genus Trichoteleia is an example that fits this pattern with 42 species known only from Madagascar and the surrounding islands. More than half of the species treated in this revision are represented by five or fewer specimens, and many are known from singletons, suggesting that more species remain to be discovered. The extraordinary explosion of species presented in this paper highlights the importance of faunal exploration in Madagascar and other regions with rapidly vanishing biodiversity.

The genus Trichoteleia was described by J. J. Kieffer (1910) to contain one species, Trichoteleia albidipes Kieffer, which was based on a single male specimen collected in Madagascar during an expedition of the German zoologist Alfred Voeltzkow. Alan Dodd described 6 species as Trichoteleia between 1914 and 1920, all of which were eventually transferred to Calliscelio Ashmead (Galloway 1976, Masner 1976), Styloteleia Kieffer (Dodd 1929, Masner 1976) and Baryconus Förster (Masner 1965). Masner (1976) transferred Alloteleia pauliani Risbec to Trichoteleia. At the time of that publication Masner was unable to locate the holotype of Trichoteleia albidipes in Berlin, and all of the platygastroids described by Kieffer from the Voeltzkow expedition are now considered to be lost.

The goal of this paper is to reevaluate the generic concept of Trichoteleia, to provide characters by which it may be unambiguously identified, and to document its species. This work is conducted as part of the Platygastroidea Planetary Biodiversity Inventory and represents a step toward revision of the Scelionini *sensu lato* and resolution of the relationships between its constituent genera. The contributions of the authors are as follows: E.J. Talamas: character definition, generic concept development, species concept development, imaging, key development, manuscript preparation; L. Masner: generic concept development, species concept development, manuscript preparation, key preparation; N.F. Johnson: character definition, species concept development, manuscript preparation, key development. The responsible authors of the new species are indicated by their names in the heading of each description.

## Materials and methods

This work is based upon specimens deposited in the following collections, with abbreviations used in the text: CASC, California Academy of Sciences, San Francisco, CA1; CNCI, Canadian National Collection of Insects, Ottawa, Canada2; MNHN, Muséum National d’Histoire Naturelle, Paris, France3; OSUC, C.A. Triplehorn Insect Collection, Columbus, OH4; UCDC, R. M. Bohart Museum of Entomology, Davis, CA5; USNM, National Museum of Natural History, Washington, DC6.

Abbreviations and morphological terms used in text: A1, A2, ... A12: antennomere 1, 2, ... 12; claval formula: distribution of the large, multiporous basiconic sensilla on the underside of apical antennomeres of the female, with the segment interval specified followed by the number of sensilla per segment (Bin 1981); S1, S2, ... S6: metasomal sternite 1, 2, ... 6; T1, T2, ... T7: metasomal tergite 1, 2, ... 7. basal node on mandible: dorsal enlargement at base of mandible, usually with apically adjacent notch (bn; Figs 112, 142); sublateral tergal carina: longitudinal carina at the junction of dorsal and lateral faces of a metasomal tergite (stc; Fig. 84); pronotal setal patch: patch of setae along posterior pronotal sulcus (psp; Figs 14, 16); posterior vertex: area between the posterior ocelli and the occipital carina; transverse sulcus on T2: transverse sulcus along anterior margin of T2 (trs; Fig. 40); metapleural spine: posteriorly pointing process formed from the metapleural carina (mtsp; Fig. 29).

We divide the epomial carina into vertical and dorsal components. The former is not present as external ridge in Trichoteleia but we consider it to be homologous with the division line between the setaceous cervical pronotal area and the glabrous (anteriorly) lateral pronotal area.

Mikó et al. (2007) used the metapleural epicoxal carina to posteriorly delimit the metapleural triangle. This carina is not present in some species of Trichoteleia, but we use the term metapleural triangle nonetheless to refer to the same anteroventral area of the metapleuron.

The following morphological terms are illustrated and labeled to facilitate their use. Definitions and abbreviations follow Mikó et al. (2007).

Antero-admedian lines (aal; Figs 7, 10)

Axillular carina (axc: Figs 11, 12)

Central keel (ctk; Fig. 3)

Cervical pronotal area (cpa; Figs 15, 16)

Dorsal epomial carina (depc; Figs 13, 14)

Dorsal metapleural area (dmpa; Figs 30, 145)

Lateral pronotal area (lpa; Figs 15, 16)

Lateral propodeal area (lpar; Figs 25, 27)

Mesoscutal suprahumeral sulcus (shms; Figs 7, 9)

Metapleural triangle (mtp; Fig. 29)

Postacetabular sulcus (ats; Figs 15, 16)

Posterior pronotal sulcus (ppsu; Figs 11, 15)

Posterior propodeal projection (ppp; Fig. 25)

Morphological terminology otherwise follows Mason (1986), Masner (1980) and Mikó et al. (2007).

The locality data reported for primary types are not a literal transcription of the labels: some abbreviations are expanded; additional data from the collectors is also included. The holotypes should be unambiguously identifiable by means of the unique identifier or the red holotype label. The numbers prefixed with “OSUC ” and “CASENT ” are unique identifiers for the individual specimens (note the blank space after the acronyms). Details on the data associated with these specimens may be accessed at the following link, purl.oclc.org/NET/hymenoptera/hol, and entering the identifier in the form.

The species descriptions are generated by a database application, vSysLab (purl.oclc.org/NET/hymenoptera/vSysLab), designed to facilitate the generation of taxon by character data matrices, to integrate these with the existing taxonomic and specimen-level database, and to export the data both as text and as input files for other applications. The output is in the format of “Character: Character state(s).” Images were produced using AutoMontage extended-focus software. The individual images are archived at the image database at The Ohio State University (purl.oclc.org/NET/hymenoptera/specimage) and with MorphBank (www.morphbank.net), the latter also contains collections of images organized by plate. All new species have been prospectively registered with Zoobank (Polaszek et al. 2005) and other taxonomic names have been retrospectively registered therein. All names are also registered in the Hymenoptera Name Server (hns.osu.edu). Life sciences identifiers, lsids, may be resolved at the URLs specified in the footnotes or at lsid.tdwg.org.

For the purpose of this revision, species are defined as taxa diagnosable by putative autapomorphies or a unique combination of fixed character states.

## Taxonomy

### Trichoteleia

Kieffer

urn:lsid:zoobank.org:act:443BC2B3-C498-4899-8291-07F0EA347FEE

urn:lsid:biosci.ohio-state.edu:osuc_concepts:573

Trichoteleia : Kieffer 1910: 530. Original description. Type: Trichoteleia albidipes Kieffer, by monotypy. Kieffer  1910: 64, 77. (description, list of species, keyed); Kieffer  1912: 227. (description); Kieffer 1926: 269, 402. (description, keyed); Muesebeck and Walkley 1956: 405. (citation of type species); Masner 1976: 41. (description); Johnson 1992: 506. (catalog of world species).

#### Description.

Length 2.1–4.1 mm; body moderately to markedly elongate, robust.

##### Head

in dorsal view transverse to subspherical; vertex smooth to coarsely sculptured; hyperoccipital carina absent; occipital carina usually well developed and continuous medially; lateral ocellus contiguous with inner orbit of compound eye or distinctly separated from it, OOL less than or equal to diameter of lateral ocellus; compound eye large, setose; frons shallowly concave to slightly convex, usually with central keel; interantennal process present, large; antennal foramen very large; submedian carina absent; orbital carina present or absent; lower frons, laterad of orbital carina, with fanlike striae; lower frons between orbital carina and interantennal process without striae; inner ocular orbits parallel or diverging ventrally; clypeus narrow, slightly convex; malar sulcus present; gena in dorsal view variably expanded posteriorly, sculpture variable; labrum hidden behind clypeus; mandible of moderate length, apex with two or three apical, acute, teeth, teeth arrayed transversely; maxillary palpus 4-segmented, all segments cylindrical; labial palpus 2-segmented; antenna 12-merous in both sexes; radicle inserted into ventral apex of scape, strongly curved; scape more or less cylindrical, ventral surface flattened; A3 in females longer than A2; basiconic sensilla on female antenna arranged singly or in longitudinal pairs on apical antennomeres; claval formula A12–A7:1-2-2-2-2-(1,0); male antenna with tyloid on A5.

**Figures 1–6.**
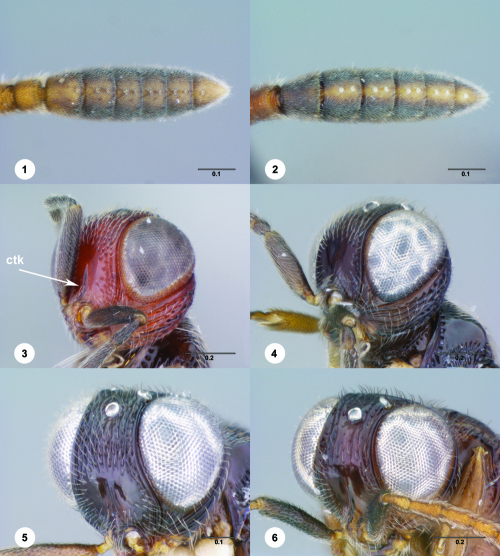
^68^ **1** Trichoteleia delilah sp. n., Antennal clava, ventral view, female (CASENT 2135905) **2** Trichoteleia orona sp. n., Antennal clava, ventral view, female (CASENT 2043194) **3** Trichoteleia hemlyae sp. n., Head, anterolateral view, female (CASENT 2043942) **4** Trichoteleia pauliani sp. n., Head, anterolateral view, female (CASENT 2136193) **5** Trichoteleia subtilis sp. n., Head, anterolateral view, female (CASENT 2134158) **6** Trichoteleia janus sp. n., Head, anterolateral view, female (CASENT 2042242). Scale bars in millimeters.

**Figures 7–12.**
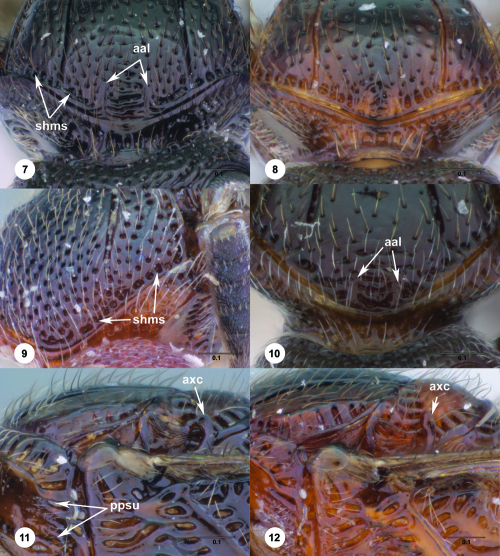
^69^ **7** Trichoteleia takariva sp. n., Antero-admedian line, anterodorsal view, male holotype (CASENT 2043217) **8** Trichoteleia janus sp. n., Antero-admedian line, anterodorsal view, male (CASENT 2043786) **9** Trichoteleia hemlyae sp. n., Notaulus, anterodorsal view, female (OSUC 181027) **10** Trichoteleia minima sp. n., Notaulus, anterodorsal view, female holotype (CASENT 2043543) **11** Trichoteleia prima sp. n., Scutellar-axillar complex, lateral view, female (CASENT 2134201) **12** Trichoteleia levii sp. n., Scutellar-axillar complex, lateral view, female (CASENT 2043789). Scale bars in millimeters.

**Figures 13–18.**
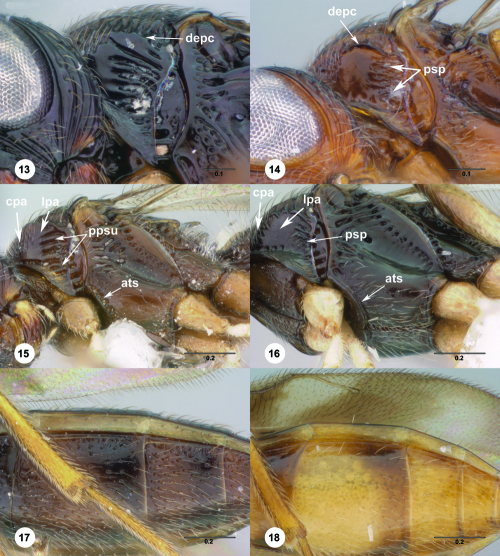
^70^ **13** Trichoteleia takariva sp. n., Pronotum, lateral view, male holotype (CASENT 2043217) **14** Trichoteleia janus sp. n., Pronotum, lateral view, male (CASENT 2043786) **15** Trichoteleia oculea sp. n., Mesosoma, ventrolateral view, female (CASENT 2135908) **16** Trichoteleia tahotra sp. n., Mesosoma ventrolateral view, female holotype (OSUC 181002) **17** Trichoteleia delilah sp. n., Metasoma, ventrolateral view, female holotype (CASENT 2135905) **18** Trichoteleia picturata sp. n., Metasoma, ventrolateral view, female holotype (CASENT 2135904). Scale bars in millimeters.

##### Mesosoma

in dorsal view longer than wide, in lateral view longer than high; pronotum in dorsal view strip-like to moderately broad laterally, anterolateral corners rounded to angulate; transverse pronotal carina present or absent; lateral pronotum without vertical epomial carina but usually with distinct transition line between sculptured cervical pronotal area and smooth lateral pronotal area; dorsal epomial carina present; lateral face of pronotum slightly to moderately concave below dorsal epomial carina, facing anterolaterally; pronotal setal patch present, accompanied by striate or rugulose sculpture; netrion present, glabrous, moderately wide, open or closed ventrally; anterior margin of mesoscutum weakly to strongly flexed ventrally to meet pronotum; mesoscutum pentagonal in outline, posterolateral corner rounded; parapsidal line visible; notaulus present, percurrent or incomplete and reaching mesoscutal suprahumeral sulcus as a row of punctures; skaphion absent; transscutal articulation well-developed; mesoscutellum transverse, narrowing laterally, posterior margin convex to straight; axilla small, dorsal margin sinuate; metanotum short, metascutellum sparsely to moderately setose, clearly differentiated, apex bifid to bispinose; plical area densely setose; lateral propodeal carina and plica well developed, forming triangular to quadrate lateral propodeal area; mesopleural depression well developed; anterior half of mesopleural carina present; posterior half of mesopleural carina present, absent or indicated by rows of punctures; anteroventral portion of mesepisternum coarsely sculptured to smooth; sternaulus not distinguishable; postacetabular foveae not distinguishable; mesopleural pit present, distinct; anterior margin of ventral portion of mesepisternum and acetabular carina transverse, not extended forward between forecoxae; mesepimeral sulcus indicated by dorsoventral line of punctures or crenulae; posterodorsal corner of mesepimeron prominent, rounded or angulate, not produced into sharp posteriorly directed tooth; mesopleuron with a strong longitudinal ledge or row of robust longitudinal carinae below subalar pit, dorsally delimiting mesopleural furrow; anteroventral portion of metapleuron continuous with lateral face; metapleural triangle often setose; metapleural epicoxal carina present or absent; metapleural epicoxal sulcus absent; paracoxal sulcus present as a dorsoventral line of cells or punctures; metapleural sulcus present; metapleural pit present or absent; posterior margin of metapleuron narrowly lamellate; legs not unusually proportioned; posterior surface of hind coxa smooth, sparsely setose to glabrous; trochantellus absent; tibial spur formula 1-1-1; tarsal formula 5-5-5; pretarsal claws simple.

##### Wings

hyaline to infuscate, often banded or patterned; Sc+R (submarginal vein) straight basal to intersection with Rs+M (basal vein), curved costad apically; R (marginal vein) present, shorter than r (stigmal vein); R1 (postmarginal vein) as long as r or longer; bulla absent; no other tracheate veins in forewing; M+Cu and Rs+M indicated by folds or pigmentation; M (medial vein), Cu (cubital vein), and Rs usually present as folds or lines of faint pigmentation; hindwing with tracheate portion of R present and reaching anterior margin; three hamuli present.

##### Metasoma

generally flattened dorsally; female with 6 terga, 6 sterna visible externally, male with 8 terga, 7 sterna visible externally; submarginal ridge well-developed, defined by narrow laterotergites to form submarginal rim; no spiracles visible; anterior part of T1 laterally compressed, with lateral depression filled with fine setae; T2 with transverse sulcus; female T6 without median raised field of microsetae or secretion; basal transverse ridge on S2 present; lateral S2 with longitudinal depression filled with fine setae; ovipositor tubular, *Scelio*-type.

#### Diagnosis.

Trichoteleia may be distinguished from other genera of Scelioninae by the combination of the following characters: eyes setose; facial striae absent between orbital carina and interantennal process, R1 (postmarginal vein) as long as r (stigmal vein) or longer; metascutellum bifid to bispinose, setose; lateral T1 and S2 with patches of dense fine setae.

#### Comments.

The species of Trichoteleia exhibit remarkable diversity in size, patterns of sculpture and color, and relative proportions of the body. The variety of sizes and shapes found in this genus suggest a corresponding diversity of host species. No host has been recorded, but the species of Trichoteleia are suspected to parasitize the eggs of ground crickets (Gryllidae). This assumption is based on the putative relation of this genus to Paridris, which has one documented host association with Gryllus pennsylvanicus Burmeister (label data of a specimen in the USNM reported by Masner and Muesebeck, 1968).

**Figures 19–24.**
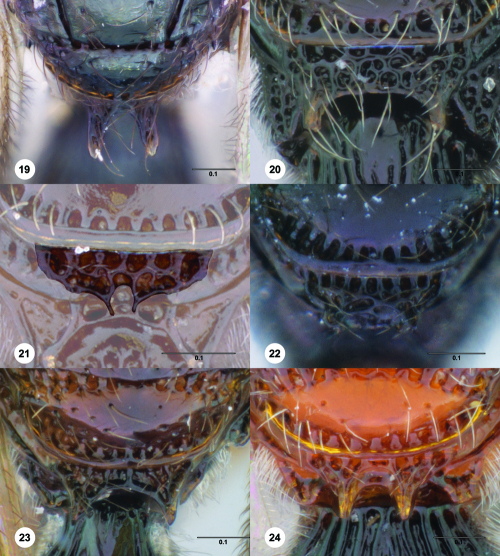
^71^ **19** Trichoteleia tahotra sp. n., Metascutellum, anterodorsal view, female (OSUC 181001) **20** Trichoteleia bidentata sp. n., Metascutellum, dorsal view, female holotype (CASENT 2136543) **21** Trichoteleia minima sp. n. Metascutellum, dorsal view, female holotype (CASENT 2043543) **22** Trichoteleia tigris sp. n., Metascutellum, dorsal view, male (CASENT 2135966) **23** Trichoteleia janus sp. n., Metascutellum, dorsal view, female (CASENT 2042242) **24** Trichoteleia afo sp. n., Metascutellum, dorsal view, female (CASENT 2043326). Scale bars in millimeters.

The shape of the metascutellum is particularly plastic. It may be present as two large spines (Fig. 19), an apically bispinose plate (Fig. 20), or a narrow strip that is notched medially (Fig. 21). The setation of the metascutellum is always present and is thus a more unambiguously interpreted character for generic identification. Within Platygastroidea, such setation is rare, and is otherwise known to the authors only in Chromoteleia Ashmead, Romilius Walker, one species of Paridris Kieffer, one species of Triteleia Kieffer and two species in the subfamily Teleasinae.

#### Link to Distribution Map.

Trichoteleia is endemic to Madagascar, the Comoros and the island of Mayotte.

#### Key to species of Trichoteleia

##### Females

(unknown for Trichoteleia takariva, Trichoteleia carinata)

**Table:** 

1	Posterior vertex covered by coarse, concentric, semicircular rugae (Figs 64, 135, 153)	2
–	Posterior vertex punctate to rugose, if rugae present then fine, irregular, or broadly interrupted medially (Figs 189, 207, 266)	4
2	Striae of pronotum reaching mesoscutal suprahumeral sulcus anteriorly (Fig. 62)	Trichoteleia albidipes Kieffer
–	Striae of pronotum separated from mesoscutal suprahumeral sulcus by smooth area (Figs 133, 151)	3
3	A7 with basiconic sensillum (as in Fig. 1); T2–T4 with microsculpture throughout (Fig. 136); T2 weakly striate, few striae reaching posterior margin; T6 densely microsculptured throughout (Fig. 137); metasoma more than 1.5 times as long as head and mesosoma combined (Figs 132, 134)	Trichoteleia irwini Talamas & Masner, sp. n.
–	A7 without basiconic sensillum (as in Fig. 2); T2–T4 without microsculpture (Fig. 155); T2 distinctly striate throughout, striae reaching posterior margin; T6 with small sparse punctures, otherwise smooth; metasoma less than 1.4 times as long as head and mesosoma combined (Figs 150, 152)	Trichoteleia ketrona Talamas, sp. n.
4	Striae of pronotal shoulder forming uniform and continuous band with striae of posterior pronotal sulcus (Figs 11, 12, 157, 211, 282); posterior vertex with fine punctation, punctation sparse to moderately dense, without rugae (Figs 159, 213, 284)	5
–	Posterior margin of pronotum with different patterns of sculpture above and below dorsal epomial carina (Fig. 252) or posterior vertex rugose (Fig. 278)	7
5	T6 densely microsculptured throughout (Fig. 286)	Trichoteleia tonsa Talamas, sp. n.
–	T6 with microsculpture or fine rugulae along anterior margin, otherwise smooth with sparse punctation (Figs 161, 215)	6
6	Metapleural triangle smooth, sometimes with very fine, sparse setae (Fig. 157); axillular carina nearly straight and perpendicular to longitudinal axis of body (Fig. 12)	Trichoteleia levii Talamas & Johnson, sp. n.
–	Metapleural triangle coarsely sculptured (Fig. 211); axillular carina rounded posteriorly (Fig. 11)	Trichoteleia prima Talamas, sp. n.
7	Forewing with purple metallic sheen (Figs 32, 167, 226); metasoma elongate, dark brown to black (Figs 167, 226)	8
–	Forewing without purple metallic sheen; length and color of metasoma variable	9
8	S2 with median longitudinal carina (as in Fig. 45); wings darkly infuscate (Figs 32, 184, 226); hind coxa yellow (Fig. 222)	Trichoteleia quazii Talamas, sp. n.
–	S2 without median longitudinal carina; wings moderately infuscate (Fig. 167); hind coxa brown (Fig. 163)	Trichoteleia longiventris Talamas & Masner, sp. n.
9	Notaulus incomplete or reaching suprahumeral sulcus as a row of punctures (Figs 9, 10); frons below median ocellus evenly punctate throughout (Figs 130, 202, 273), rarely with dorsoventral rugulae laterally; S2 punctate, punctation usually coarse and uniform throughout (Fig. 43)	10
–	Notaulus complete and reaching suprahumeral sulcus as a smooth furrow (Figs 7, 8); frons between median ocellus and antennal scrobe variably sculptured; S2 punctate, smooth or longitudinally striate	23
10	Mandible bidentate (Fig. 77); lateral propodeal area punctate rugose (Fig. 27)	11
–	Mandible tridentate; sculpture of lateral propodeal area variable	12
11	Metasoma elongate, length of T5 more than 2.5 times width of posterior margin (Fig. 220); gena broad, with small punctures and well-defined striae (Fig. 217)	Trichoteleia prolixa Talamas, sp. n.
–	Metasoma moderately long, length of T5 less than 2 times width of posterior margin (Fig. 78); gena not broad, with striation confused by dense punctation (Fig. 74)	Trichoteleia bidentata Talamas, sp. n.
12	M+Cu and Rs+M veins in forewing spectral, i.e., without pigment, visible only as fold in wing membrane (Fig. 35)	13
–	M+Cu and Rs+M veins in forewing nebulous, i.e., indicated by diffuse pigmented lines (Figs 31–34, 36)	20
13	T1 produced anteriorly into horn, horn sometimes small but always with dorsal surface smooth and convex (Figs 67, 246, 272, 298)	14
–	T1 without horn (Figs 55, 138, 173)	18
14	Forewing with transverse infuscate band medially (Fig. 72); lateral T4 striate throughout (Fig. 72); sculpture of medial T3 reticulate rugose (Fig. 72)	Trichoteleia bicolor Talamas, sp. n.
–	Forewing hyaline, sometimes with diffuse longitudinal infuscation (Figs 31, 247, 271, 298); sculpture of T4 and medial T3 variable (Figs 250, 274, 298)	15
15	Posterior margin of transverse sulcus on T2 straight (Fig. 298); head and metasoma black, mesosoma red (Figs 293, 295); mandibles dark brown to black (Fig. 297)	Trichoteleia xantrox Talamas, sp. n.
–	Posterior margin of transverse sulcus on T2 convex (Figs 250, 274); body color pattern not as above; color of mandibles variable	16
16	Lateral T4–T5 longitudinally strigose (Fig. 119)	Trichoteleia funesta Talamas, sp. n.
–	T4 smooth or with faint striae; T5 without macrosculpture	17
17	T5–T6 sparsely punctate (Fig. 250); gena punctate rugose, broad (Fig. 246); head, mesosoma and metasoma brown (Fig. 245)	Trichoteleia sphaerica Talamas, sp. n.
–	T5–T6 densely punctate (Fig. 274); gena dorsoventrally strigose, moderately narrow (Fig. 270); head, mesosoma and metasoma black (Fig. 269)	Trichoteleia tezitra Talamas, sp. n.
18	Forewing with faint infuscation below R (marginal vein) (Fig. 149); metasoma black, head and mesosoma orange (Fig. 144)	Trichoteleia jiro Talamas, sp. n.
–	Forewing with percurrent transverse infuscate band medially (Figs 57, 170); color pattern variable	19
19	Metascutellar armature short, posterior margin with small median notch (Fig. 21); T2 longitudinally strigose (Fig. 173); T2–T3 with microsculpture present interstitially (Fig. 173); occipital carina bordered anteriorly by large cells (Fig. 171)	Trichoteleia minima Talamas, sp. n.
–	Metascutellum markedly bispinose, posterior margin distinctly emarginate (Fig. 58); T2 reticulate rugose (Fig. 60); T2–T3 without microsculpture (Fig. 60); occipital carina bordered anteriorly by medium sized cells (Fig. 58)	Trichoteleia afo Talamas, sp. n.
20	T2–T4 without microsculpture (Fig. 101); S2 smooth with sparse fine punctation	Trichoteleia eburata Talamas, sp. n.
–	T2–T4 with microsculpture throughout (Figs 131, 203, 232); S2 variably sculptured	21
21	Central of keel of frons absent (Fig. 4), if faintly indicated, then not extending onto interantennal process (Fig. 202); metapleural triangle smooth to sparsely and finely punctate (Fig. 199); setation of metapleural triangle absent to sparse (Fig. 199)	Trichoteleia pauliani (Risbec)
–	Central keel of frons present and extending onto interantennal process (Figs 3, 130); metapleural triangle coarsely sculptured, densely and finely punctate to rugose (Figs 127, 228); setation of metapleural triangle sparse to moderately dense (Figs 127, 228)	22
22	Apical half of forewing with diffuse infuscation along longitudinal midline (Fig. 31)	Trichoteleia hemlyae Talamas & Masner, sp. n.
–	Forewing with transverse infuscate band below R (marginal vein) and infuscate patch distally (Fig. 231)	Trichoteleia avaka Talamas & Masner, sp. n.
23	Brachypterous, apex of forewing ending before midpoint of T3 (Figs 122, 192)	24
–	Macropterous, apex or forewing extending beyond posterior margin of T3	26
24	Horn on T1 with posterodorsally oriented spine at apex (Fig. 102); striae of lateral T4 merging medially in anterior half of tergite (Fig. 107); metascutellar spines large, diverging apically, with large black setae (Figs 103, 105)	Trichoteleia echinata Talamas, sp. n.
–	Horn on T1 smooth and convex dorsally, without spine (Fig. 121); striae of lateral T4 separated medially by smooth midline field (Figs 125, 197); metascutellar spines variable	25
25	A7 without basiconic sensillum (as in Fig. 2); lateral T3 and entire surface of T4–T5 obliquely strigose (Fig. 125); basal node on mandible absent (Fig. 124, as in Fig. 255)	Trichoteleia halterata Talamas & Masner, sp. n.
–	A7 with basiconic sensillum (as in Fig. 1); T3, T5 smooth with sparse punctation, lateral T4 sometimes with weak crenulae along anterior margin, otherwise smooth (Fig. 197); basal node on mandible large (Fig. 196)	Trichoteleia parvipennis Talamas & Masner, sp. n.
26	Medial frons rugulose (Fig. 279); horn on T1 absent (Fig. 275); posterior vertex coarsely punctate rugose throughout (Fig. 278); forewing with two transverse infuscate bands (Fig. 280)	Trichoteleia tigris Talamas, sp. n.
–	Medial frons smooth (Figs 89, 95); horn on T1 present or absent; posterior vertex variable, but not coarsely punctate rugose throughout; color of forewing variable	27
27	Forewing patterned with distinct bands or contrasting infuscate and hyaline areas (Figs 33, 96); postacetabular sulcus present as a smooth furrow (as in Fig. 16); T6 smooth with fine setigerous punctures throughout or at lateral margin (Fig. 303)	28
–	Forewing hyaline to infuscate but without banding; postacetabular sulcus variable; sculpture and punctation of T6 variable	31
28	A7 with basiconic sensillum (Fig. 1); T2–T3 with microsculpture throughout (Fig. 96); width of T1 along posterior margin greater than 1.6 times length (Fig. 96)	Trichoteleia delilah Talamas, sp. n.
–	A7 without basiconic sensillum (as in Fig. 2); T2–T3 without microsculpture (Figs 90, 209, 301); width of T1 along posterior margin less than 1.2 times length (Figs 90, 209, 301)	29
29	Frons below median ocellus dorsoventrally strigose (Fig. 89)	Trichoteleia cincta Talamas & Masner, sp. n.
–	Frons below median ocellus punctate or punctate rugulose (Figs 208, 304)	30
30	Posterior half of medial mesoscutum longitudinally rugulose (Fig. 207)	Trichoteleia picturata Talamas, sp. n.
–	Posterior half of medial mesoscutum moderately punctate, otherwise smooth (Fig. 302)	Trichoteleia zuparkoi Talamas & Masner, sp. n.
31	Horn on T1 hooked, pointing posteriorly at apex (Fig. 109); metascutellar spines stout and diverging distally (Fig. 111)	Trichoteleia fisheri Talamas & Masner, sp. n.
–	Horn on T1, if present, smooth or with longitudinal carina at apex, but not curved or pointed posteriorly (Fig. 240); metascutellar spines variable	32
32	R1 (postmarginal vein) equal in length to r (stigmal vein) (Fig. 36); lateral propodeal area with small apical flange (Figs 25, 26); horn on T1 absent (Figs 180, 262)	33
–	R1 (postmarginal vein) distinctly longer than r (stigmal vein) (Figs 31–33, 35); lateral propodeal area without apical flange (Figs 27–28); horn on T1 usually present	34
33	Lateral pronotum with coarse striae extending nearly to cervical pronotal area (Fig. 15); A7 without basiconic sensillum (as in Fig. 2); postacetabular sulcus comprised of large cells (Fig. 15); T4 punctate except along midline (Fig. 185)	Trichoteleia oculea Talamas, sp. n.
–	Lateral pronotum with short irregular striae or rugulae posteriorly (Fig. 16); A7 with basiconic sensillum (as in Fig. 1); postacetabular sulcus present as a smooth furrow (Fig. 16); T4 devoid of punctation and sculpture except along lateral margins (Fig. 262)	Trichoteleia tahotra Talamas & Masner, sp. n.
34	T3 transversely strigose (Fig. 191); frons below median ocellus dorsoventrally striate (Fig. 190); lateral pronotum with pronounced longitudinal striae (Fig. 187); interantennal process enlarged dorsally (Fig. 187); body entirely black	Trichoteleia orona Talamas & Masner, sp. n.
–	T3 smooth or with longitudinally oriented lines of sculpture; other characters variable but not in the combination above	35
35	Posterior margin of metapleuron with posterolaterally projecting triangular spine near intersection with metapleural sulus (Fig. 29); setal patch on lateral T1 bordered by flange dorsally (Fig. 29); hind wing with density of microtrichae posterior to Sc+R (submarginal vein) reduced relative to remainder of wing membrane (Fig. 34)	Trichoteleia nify Talamas & Masner, sp. n.
–	Posterior margin of metapleuron smooth or bluntly angulate near intersection with metapleural sulcus (Figs 139, 252); setal patch on lateral T1 with carina dorsally but without flange; hind wing with density of microtrichae uniform throughout (Figs 31–33)	36
36	Lateral T4 smooth or with small patch of faint strigae (Figs 143, 256); T5 with sparse punctation, otherwise smooth (Figs 143, 256)	37
–	Lateral T4–T5 with percurrent longitudinal strigae or rugulae (Figs 238, 244, 292); T5 with moderately dense, large punctures laterally (Figs 238, 244, 292)	38
37	Mandibular node present, notch present just distad of node (Fig. 142); gena with dorsoventral rugae (Fig. 139); central keel on frons extending onto dorsal surface of interantennal process (Fig. 6)	Trichoteleia janus Talamas, sp. n.
–	Mandibular node and notch absent (Fig. 255); gena with fine dorsoventral striae (Fig. 252); central keel on frons ventrally attenuating or diverging laterally around interantennal process (Fig. 255)	Trichoteleia subtilis Talamas & Masner, sp. n.
38	Frons below median ocellus evenly dorsoventrally strigose throughout (Fig. 291)	Trichoteleia warreni Talamas & Masner, sp. n.
–	Frons below median ocellus smooth or with dorsoventral strigae that attenuate medially (Figs 243, 237)	39
39	Rugae laterad of median ocellus prominent (Fig. 237); mesoscutum densely punctate, punctures moderately coarse (Fig. 236); cells of occipital rim large (Fig. 236); body dark brown to black	Trichoteleia rugifrons Talamas & Masner, sp. n.
–	Rugae laterad of median ocellus faint or absent (Fig. 243); mesoscutum with moderately dense punctation, punctures small (Fig. 242); cells of occipital rim small (Fig. 242); body yellow throughout	Trichoteleia solocis Talamas, sp. n.

##### Males

(unknown for Trichoteleia cincta, Trichoteleia delilah, Trichoteleia echinata, Trichoteleia fisheri, Trichoteleia funesta, Trichoteleia halterata, Trichoteleia ketrona, Trichoteleia longiventris, Trichoteleia oculea, Trichoteleia minima, Trichoteleia parvipennis, Trichoteleia picturata, Trichoteleia prima, Trichoteleia prolixa, Trichoteleia ravaka, Trichoteleia solocis, Trichoteleia sphaerica, Trichoteleia warreni, Trichoteleia xantrox and Trichoteleia zuparkoi)

**Table:** 

1	Posterior vertex covered by coarse, concentric, semicircular rugae (Figs 64, 135)	2
–	Posterior vertex punctate to rugose, if rugae present then fine, irregular, or broadly interrupted medially (Figs 189, 266, 278)	3
2	Striae of pronotum reaching mesoscutal suprahumeral sulcus anteriorly (Fig. 62)	Trichoteleia albidipes Kieffer
–	Striae of pronotum separated from mesoscutal suprahumeral sulcus by smooth area (Fig. 133)	Trichoteleia irwini Talamas & Masner, sp. n.
3	Striae of pronotal shoulder forming uniform and continuous band with striae of posterior pronotal sulcus (Figs 12, 157, 282, as in 11, 211); posterior vertex with fine punctation, punctation sparse to moderately dense, without rugae (Figs 159, 284); metapleural triangle smooth or with fine sparse punctures (Figs 157, 282)	4
–	Posterior margin of pronotum with different patterns of sculpture above and below dorsal epomial carina (Figs 222, 246), or posterior vertex rugose (Figs 266, 278); metapleural triangle usually coarsely punctate or rugulose	5
4	Axillular carina nearly straight and perpendicular to longitudinal axis of body (Fig. 12); mesosoma dark red to yellow (Figs 12, 157, 305)	Trichoteleia levii Talamas & Johnson, sp. n.
–	Axillular carina rounded posteriorly (as in Fig. 11); mesosoma dark brown throughout (Fig. 282)	Trichoteleia tonsa Talamas, sp. n.
5	S2 coarsely punctate throughout with longitudinal median carina (Fig. 45); metasoma dark brown to black, elongate (Figs 81, 225)	6
–	Sculpture of S2 variable, if coarsely punctate then without longitudinal median carina; color and length of metasoma variable	7
6	T2 with prominent sublateral carina (. 84); posterior margin of transverse sulcus on T2 straight between sublateral carinae (Fig. 84); T2 1.5 times as long as wide (Fig. 84); wings slightly infuscate (Fig. 81)	Trichoteleia carinata Talamas & Johnson, sp. n.
–	T2 with longitudinal strigae of equal height throughout (Fig. 225); posterior margin of transverse sulcus on T2 convex (Fig. 225); T2 less than 1.2 times as long as wide (Fig. 225); wings darkly infuscate throughout (Fig. 225)	Trichoteleia quazii Talamas, sp. n.
7	Notaulus incomplete or reaching mesoscutal suprahumeral sulcus as a row of punctures (Figs 9, 10); frons below median ocellus evenly punctate throughout (Figs 130, 202, 273), rarely with dorsoventral rugulae laterally; S2 punctate, punctation usually coarse and uniform throughout (Fig. 43)	8
–	Notaulus complete and reaching mesoscutal suprahumeral sulcus as a smooth furrow (Fig. 7, 8); sculpture of frons below median ocellus variable; S2 punctate, smooth, or longitudinally striate	14
8	M+Cu and Rs+M in forewing spectral, i.e., not pigmented (Fig. 35)	9
–	M+Cu and Rs+M in forewing nebulous, i.e., pigmented (Figs 31–34, 36, 231)	13
9	Mandible bidentate (Fig. 77); lateral propodeal area punctate rugose (Fig. 27)	Trichoteleia bidentata Talamas, sp. n.
–	Mandible tridentate; sculpture of lateral propodeal area variable	10
10	T3 longitudinally strigose (Fig. 274)	Trichoteleia tezitra Talamas & Masner, sp. n.
–	T3 reticulate to reticulate rugose (Figs 60, 149)	11
11	T2–T4 without interstitial microsculpture (Fig. 41); forewing with infuscate band medially (Fig. 35); width of posterior margin of T1 more than 2 times length of T1 (Fig. 41)	Trichoteleia afo Talamas & Masner, sp. n.
–	T2–T4 with interstitial microsculpture present (Fig. 42); forewing hyaline throughout (Fig. 42); width of posterior margin of T1 less than 2 times length of T1 (Fig. 42)	12
12	Dorsal metapleural area with smooth glabrous strip (Fig. 52); head and mesosoma entirely black (Figs 51, 52)	Trichoteleia bicolor Talamas, sp. n.
–	Dorsal metapleural area entirely punctate rugose (Fig. 50); head and mesosoma orange to pale brown becoming darker dorsally (Figs 49, 50)	Trichoteleia jiro Talamas & Masner, sp. n.
13	Central of keel of frons absent (Fig. 4), if faintly present then not extending onto interantennal process (Fig. 202); metapleural triangle mostly smooth, with very sparse fine punctures along anteroventral perimeter (Fig. 199)	Trichoteleia pauliani (Risbec)
–	Central keel of frons present and extending onto interantennal process (Figs 3, 130); metapleural triangle rugulose with fine to coarse punctation (Fig. 127) or coarsely punctate rugose	Trichoteleia hemlyae Talamas & Masner, sp. n.
14	R1 (postmarginal vein) equal in length to r (stigmal vein) (Fig. 36); postacetabular sulcus present as a smooth furrow (Fig. 16); posterior propodeal projection present as small apical flange (Fig. 25)	Trichoteleia tahotra Talamas & Masner, sp. n.
–	R1 (postmarginal vein) longer than r (stigmal vein) (as in Figs 31–32); postacetabular sulcus comprised of cells, cells of variable size (Figs 139, 187, 234, as in Fig. 15); posterior propodeal projection absent (Fig. 28, as in Fig. 27)	15
15	Lateral propodeal area large, mostly smooth (Figs 28, 53); T2–T3 densely microsculptured (Fig. 54); smooth area on frons small; metascutellar spines short, blunt (Figs 28, 53); posterior vertex coarsely punctate rugose (Fig. 53); occipital rim comprised of large cells (Fig. 53); frons below median ocellus densely punctate, without dorsoventral strigae (Fig. 279)	Trichoteleia tigris Talamas, sp. n.
–	Lateral propodeal area small to moderate sized, if large then coarsely rugose; T2–T3 microsculpture absent or very shallowly impressed; smooth area on medial frons large; other characters variable but without the combination above	16
16	Posterior margin of metapleuron with small posterolaterally projecting spine near intersection with metapleural sulcus (Fig. 29); density of microtrichae on hind wing reduced posterior to Sc+R (as in Fig. 34); T3 smooth or with very faint traces of longitudinal sculpture (Fig. 37)	Trichoteleia nify Talamas & Masner, sp. n.
–	Metapleural spine absent, sometimes a blunt angular projection present on posterior margin (Fig. 139); density of microtrichae uniform throughout hind wing (Figs 31–33); sculpture of T3 variable	17
17	T5 densely punctate throughout (Fig. 40); interantennal process with large dorsal flange (Fig. 187); frons below median ocellus dorsoventrally strigose (Fig. 190)	Trichoteleia orona Talamas & Masner, sp. n.
–	T5 sparsely to moderately punctate (Fig. 265), punctation usually reduced medially, crenulae sometimes present laterally; interantennal process simple or with dorsal carina, rarely with small dorsal flange; frons below median ocellus variable	18
18	Area between antero-admedian lines with parallel transverse rugae (Fig. 7); pronotal cervical sulcus present as a smooth furrow; body black (Fig. 263)	Trichoteleia takariva Talamas, sp. n.
–	Area between antero-admedian lines punctate, very rarely with 1–2 rugae (Fig. 8); other characters variable but without the combination above	19
19	T5 punctate rugulose (Fig. 238); lateral T4 with longitudinal strigae or reticulations (Fig. 238)	Trichoteleia rugifrons Talamas & Masner, sp. n.
–	T5 smooth between punctures (Figs 143, 256); lateral T4 smooth or with small patch of very faint striae or crenulae (Figs 143, 256)	20
20	Central keel on frons ventrally bifurcating around interantennal process (Figs 5, 255); gena finely striate or punctate rugulose (Fig. 252)	Trichoteleia subtilis Talamas & Masner, sp. n.
–	Central keel on frons extending onto dorsal surface of interantennal process (Figs 6, 139); gena coarsely strigose (Fig. 139)	Trichoteleia janus Talamas, sp. n.

### Trichoteleia afo

Talamas sp. n.

urn:lsid:zoobank.org:act:FD95381D-74B9-483F-AC17-89E6F4539047

urn:lsid.biosci.ohio-state.edu:osuc_concepts: 241291

Figures 24 35 41 43 55–60 Morphbank ^7^ 

#### Description.

##### Female body length:

2.67–3.06 mm (n=20). Male body length: 2.56–2.80 mm (n=21). Color of head: dark orange, becoming brown at vertex; orange throughout. Central keel of frons: present, ending above interantennal process. Sculpture of medial frons in female: smooth. Sculpture of medial frons in male: smooth. Number of mandibular teeth: three. Basal node on mandible: present. Sculpture of frons below median ocellus: punctate rugulose throughout; punctate rugulose laterally, smooth medially. Sculpture of posterior vertex: densely punctate; moderately punctate, rugose posterior to eyes and posterior ocellus. Occipital rim: comprised of small to miniscule cells. Sculpture of gena: punctate rugose; dorsoventrally strigose. Basiconic sensillum on A7: absent.

##### Color of mesosoma in female:

orange throughout. Color of mesosoma in male: variably red to black. Sculpture along posterior pronotal sulcus: rugulose. Notaulus: smooth furrow incomplete, reaching suprahumeral sulcus as row of punctures. Sculpture of medial mesoscutum: smooth and sparsely punctate posteriorly, densely punctate and transverely rugulose anteriorly. Sculpture of mesoscutellum: smooth with sparse fine punctures throughout. Postacetabular sulcus: comprised of large cells; comprised of small cells. Mesopleural carina: present. Sculpture along ventral half of prespecular sulcus: coarsely punctate. Sculpture of posterolateral mesepisternum: coarsely punctate. Sculpture of ventral surface of mesepisternum: moderately punctate. Setation of ventral metapleural area: absent. Setation of metapleural triangle: moderately dense. Sculpture of metapleural triangle: punctate rugose. Posterior margin of metapleuron below propodeal spiracle: straight to moderately convex. Color of legs: basal tibia and tarsomeres white, otherwise brown.

##### Color of metasoma in female:

black. Color of metasoma in male: dark brown to black throughout. Posterior margin of transverse sulcus on T2: straight. Sublateral tergal carina on T2: absent. Microsculpture on T2: absent. Microsculpture on T3: absent. Microsculpture on T4: absent. Horn on T1 in female: absent. Macrosculpture of T2 in female: reticulate rugose throughout. Macrosculpture of medial T3 in female: reticulate rugose. Macrosculpture of lateral T3 in female: reticulate rugose. Macrosculpture of medial T4 in female: punctate rugose; reticulate. Macrosculpture of lateral T4 in female: punctate rugulose. Punctation of T4 in female: moderately dense throughout. Macrosculpture of T5 in female: absent. Punctation of T5 in female: sparse along midline, otherwise dense. Shape of T5 in female: width of posterior margin greater than or equal to length. Microscupture on T6 in female: absent. Sculpture of T6 in female: smooth with sparse moderate sized punctures. Macrosculpture of T2 in male: reticulate rugose throughout. Macrosculpture of medial T3 in male: reticulate rugose. Macrosculpture of lateral T3 in male: reticulate rugose. Macrosculpture of T4 in male: rugulose throughout. Punctation of T4 in male: moderately dense throughout. Macrosculpture of T5 in male: absent. Punctation of T5 in male: dense throughout. Sculpture of S2: coarsely punctate throughout. Prominent longitudinal median carina on S2: absent.

##### Wings:

macropterous, apex or forewing extending beyond posterior margin of T3. Color of forewing in female: hyaline with transverse infuscate band medially. Color of forewing in male: hyaline with transverse infuscate band medially. Color of hind wing: hyaline throughout. Density of setation in fore wing: uniform throughout. Density of setation in hind wing: uniform throughout. Length of R1: more than 1.5 times as long as r. M+Cu and RS+M in forewing: spectral.

#### Diagnosis.

This species is most similar to Trichoteleia jiro in color pattern, size, and habitus. It may be distinguished by the coarse reticulate rugose sculpture of T2–T3 (Figs 41, 60) and the single transverse band in the forewing (Fig. 35).

#### Etymology.

This species is given the name *afo*, meaning “fire” in Malagasy, for the bright red color of the head and mesosoma in females. The epithet is used as a noun in apposition.

#### Link to Distribution Map.

[http://hol.osu.edu/map-large.html?id=241291]

#### Material Examined.

Holotype, female: **MADAGASCAR:** Antsiranana Auto. Prov., brushy area, MA-01-04A-02, 1km W Sakalava Beach, 12°15'59"S, 49°23'42"E, 30m, 23.I–27.I.2001, malaise trap, M. E. Irwin, E. I. Schlinger & R. Harin’Hala, OSUC 143346 (deposited in CASC). Paratypes: **MADAGASCAR:** 21 females, 30 males, CASENT 2042214, 2042915–2042916, 2043326–2043327, 2043339, 2043391, 2043512–2043513, 2043775–2043781, 2043783, 2133948, 2134576, 2134804, 2134839, 2136213–2136217, 2136577, 2136579, 2136582, 2136589, 2136598–2136599, 2136603, 2137670, OSUC 143327 (CASC); OSUC 143344, 186062 (CNCI); CASENT 2042728, 2137960, OSUC 143325, OSUC 143326, OSUC 143328, OSUC 143330, OSUC 143331, OSUC 143332, OSUC 143342, OSUC 143343, OSUC 143345, OSUC 186063, OSUC 186064, OSUC 186065 (OSUC).

### Trichoteleia albidipes

Kieffer

urn:lsid:zoobank.org:act:1335620B-6439-47E8-AE58-D1292D033DE1

urn:lsid.biosci.ohio-state.edu:osuc_concepts:5507

Figures 61–66 Morphbank ^8^

Trichoteleia albidipes Kieffer 1910: 531 (original description); Kieffer 1926: 402 (description); Risbec 1956: 252 (description of male).

#### Notes.

In the introduction of the paper in which this species was described, Kieffer (1910) stated that the specimens would be deposited in the Königliche Zoologische Museum in Berlin. However, the curator of Hymenoptera reports that the type species of the genera of Scelioninae described by Kieffer are not present. We conclude that this material was probably lost as a consequence of damage to that institution in World War II. We believe that the establishment of a neotype for Trichoteleia albidipes is highly desirable in order to stabilize interpretation of the genus Trichoteleia. Kieffer’s original description contained three characters that, in combination, we believe enable us to identify this species among the examined specimens. These are: cheeks with striae meeting medially at vertex, T2–T3 finely longitudinally striate, and mesopleuron horizontally striate. The collection locality of the neotype is less than 50 miles from that of the original holotype specimen. No host data are known for Trichoteleia, therefore it is impossible to be certain that the neotype parasitizes the same host as the lost holotype. In accordance with article 75 of the Code, specimen CASENT 2132453 (deposited in CASC) is designated as the neotype of Trichoteleia albidipes.

#### Description.

##### Female body length:

2.57–3.25 mm (n=19). Male body length: 2.36–2.94 mm (n=6). Color of head: dark brown to black. Central keel of frons: present, extending onto interantennal process. Sculpture of medial frons in female: smooth. Sculpture of medial frons in male: smooth. Number of mandibular teeth: three. Basal node on mandible: present. Sculpture of frons below median ocellus: punctate throughout with a groove parallel to orbital carina. Sculpture of posterior vertex: concentrically rugose. Sculpture of gena: coarsely striate. Basiconic sensillum on A7: absent.

##### Color of mesosoma in female:

dark brown to black. Color of mesosoma in male: dark brown to black. Sculpture along posterior pronotal sulcus: striate, striae reaching dorsal pronotum. Notaulus: percurrent, reaching suprahumeral sulcus as a smooth furrow. Sculpture of medial mesoscutum: moderately punctate in posterior half, becoming denser anteriorly. Sculpture of mesoscutellum: smooth with sparse fine punctures throughout. Postacetabular sulcus: comprised of small cells. Mesopleural carina: present; present only in anterior half. Sculpture along ventral half of prespecular sulcus: longitudinally striate. Sculpture of posterolateral mesepisternum: smooth; smooth except for 1 or 2 striae parallel to mesopleural carina. Sculpture of ventral surface of mesepisternum: smooth. Setation of ventral metapleural area: absent. Setation of metapleural triangle: sparse. Sculpture of metapleural triangle: smooth; finely punctate. Posterior margin of metapleuron below propodeal spiracle: with blunt kink near intersection with metapleural sulcus. Color of legs: coxae and trochanters yellow, otherwise pale brown, hindlegs the darkest.

##### Color of metasoma in female:

Color of metasoma in female: dark brown to black throughout. Color of metasoma in male: dark brown to black throughout. Posterior margin of transverse sulcus on T2: strongly convex. Sublateral tergal carina on T2: absent. Microsculpture on T2: present. Microsculpture on T3: present. Microsculpture on T4: absent. Horn on T1 in female: present as a large, apically rounded protuberance. Macrosculpture of T2 in female: longitudinally striate throughout; weakly longitudinally striate throughout. Macrosculpture of medial T3 in female: weakly longitudinally striate; longitudinally striate. Macrosculpture of lateral T3 in female: longitudinally striate; weakly longitudinally striate. Macrosculpture of medial T4 in female: absent. Macrosculpture of lateral T4 in female: weakly longitudinally strigose; absent. Punctation of T4 in female: absent in medial third, sparse laterally; sparse throughout. Macrosculpture of T5 in female: absent. Punctation of T5 in female: moderately dense throughout; dense throughout; absent along midline, otherwise dense. Shape of T5 in female: width of posterior margin greater than or equal to length. Microscupture on T6 in female: absent. Sculpture of T6 in female: smooth with sparse fine setigerous punctures throughout. Macrosculpture of T2 in male: longitudinally striate throughout. Macrosculpture of medial T3 in male: weakly longitudinally striate. Macrosculpture of lateral T3 in male: longitudinally striate. Macrosculpture of T4 in male: longitudinally strigose laterally; absent. Punctation of T4 in male: sparse throughout. Macrosculpture of T5 in male: absent. Punctation of T5 in male: moderately dense throughout. Sculpture of S2: longitudinally striate anteriorly, smooth posteriorly. Prominent longitudinal median carina on S2: absent.

##### Wings:

Wings: macropterous, apex or forewing extending beyond posterior margin of T3. Color of forewing in female: slightly infuscate throughout. Color of forewing in male: slightly infuscate throughout. Color of hind wing: slightly infuscate throughout. Density of setation in fore wing: uniform throughout. Density of setation in hind wing: uniform throughout. Length of R1: more than 1.5 times as long as r. M+Cu and RS+M in forewing: nebulous.

#### Diagnosis.

The striae that extend from the posterior margin of the lateral pronotum to the mesoscutal suprahumeral sulcus (Fig. 62) distinguish this species of Trichoteleia from all others. It is similar to Trichoteleia irwini and to Trichoteleia ketrona in the concentric rugosity of the posterior vertex and gena.

#### Link to Distribution Map.

[http://hol.osu.edu/map-large.html?id=5507]

#### Material Examined.

Neotype, female (present designation): **MADAGASCAR:** Toamasina Auto. Prov., parcel 3, littoral forest, BLF10730, Tampolo, 17°17'00"S, 49°26'00"E, 10m, 14.IV.2004, yellow pan trap, CASENT 2132453 (deposited in CASC). Other material: **MADAGASCAR:** 21 females, 7 males, CASENT 2043341, 2132454–2132457, 2132459–2132462, 2132464, 2132475, 2132484, 2132490, 2133925, 2135944, 2136863, 2137948, OSUC 215603 (CASC); OSUC 181020 (CNCI); CASENT 2042241, 2042287, 2132458, 2132463, 2132465, 2132501, 2134750 (OSUC); OSUC 181018–181019 (SCAU).

### Trichoteleia bicolor

Talamas sp. n.

urn:lsid:zoobank.org:act:76FBEDE5-0295-4558-93B3-E1AABAC80343

urn:lsid.biosci.ohio-state.edu:osuc_concepts: 243408

Figures 51–52 67–72 307–308 Morphbank ^9^ 

#### Description.

##### Female body length:

2.74 mm (n=1). Male body length: 2.35–2.80 mm (n=2). Color of head: dark brown to black; reddish brown. Central keel of frons: present, ending above interantennal process. Sculpture of medial frons in female: smooth. Sculpture of medial frons in male: smooth. Number of mandibular teeth: three. Basal node on mandible: present. Sculpture of frons below median ocellus: dorsoventrally strigose throughout with coarsely punctate interstices. Sculpture of posterior vertex: punctate rugulose. Occipital rim: comprised of medium to large sized cells. Sculpture of gena: punctate rugose. Basiconic sensillum on A7: absent.

##### Color of mesosoma in female:

reddish brown throughout. Color of mesosoma in male: dark brown to black. Sculpture along posterior pronotal sulcus: rugulose. Notaulus: smooth furrow incomplete, reaching suprahumeral sulcus as row of punctures. Sculpture of medial mesoscutum: smooth and sparsely punctate posteriorly, densely punctate and transverely rugulose anteriorly. Sculpture of mesoscutellum: smooth with sparse fine punctures throughout. Postacetabular sulcus: comprised of small cells. Mesopleural carina: present. Sculpture along ventral half of prespecular sulcus: coarsely punctate. Sculpture of posterolateral mesepisternum: coarsely punctate. Sculpture of ventral surface of mesepisternum: moderately punctate. Setation of ventral metapleural area: absent. Setation of metapleural triangle: sparse. Sculpture of metapleural triangle: punctate rugose. Posterior margin of metapleuron below propodeal spiracle: with blunt kink near intersection with metapleural sulcus. Color of legs: brown throughout.

##### Color of metasoma in female:

reddish brown. Color of metasoma in male: dark brown to black throughout. Posterior margin of transverse sulcus on T2: straight. Sublateral tergal carina on T2: absent. Microsculpture on T2: present. Microsculpture on T3: present. Microsculpture on T4: present. Horn on T1 in female: present as a large, apically rounded protuberance. Macrosculpture of T2 in female: reticulate rugose medially, longitudinally strigose laterally. Macrosculpture of medial T3 in female: reticulate rugose. Macrosculpture of lateral T3 in female: longitudinally strigose. Macrosculpture of medial T4 in female: longitudinally strigose. Macrosculpture of lateral T4 in female: longitudinally strigose. Punctation of T4 in female: sparse throughout. Macrosculpture of T5 in female: weakly rugulose laterally. Punctation of T5 in female: moderately dense throughout. Shape of T5 in female: width of posterior margin less than length. Microscupture on T6 in female: absent. Sculpture of T6 in female: finely punctate throughout. Macrosculpture of T2 in male: reticulate rugose medially, longitudinally strigose laterally. Macrosculpture of medial T3 in male: reticulate. Macrosculpture of lateral T3 in male: longitudinally strigose. Macrosculpture of T4 in male: longitudinally strigose throughout. Punctation of T4 in male: sparse throughout. Macrosculpture of T5 in male: weakly rugulose laterally. Punctation of T5 in male: moderately dense throughout. Sculpture of S2: coarsely punctate throughout. Prominent longitudinal median carina on S2: absent.

##### Wings:

macropterous, apex or forewing extending beyond posterior margin of T3. Color of forewing in female: hyaline with transverse infuscate bands medially and apically. Color of forewing in male: hyaline throughout. Color of hind wing: hyaline throughout. Density of setation in fore wing: reduced posterior to Sc+R in basal half. Density of setation in hind wing: uniform throughout. Length of R1: more than 1.5 times as long as r. M+Cu and RS+M in forewing: spectral.

#### Diagnosis.

Females of Trichoteleia bicolor are most similar to Trichoteleia sphaerica. The present species may be distinguished from Trichoteleia sphaerica in having two transverse bands of infuscation in the forewing (Fig. 72).

#### Etymology.

This species is named for the color dimorphism in the wings and body color between males and females. The epithet is a noun in apposition.

#### Link to Distribution Map.

[http://hol.osu.edu/map-large.html?id=243408]

#### Material Examined.

Holotype, female: **MADAGASCAR:** Fianarantsoa Auto. Prov., E of Interpretive Center, stream / open area, MA-02-11A-21, nr. Isalo National Park, 22°37.60'S 45°21.49'E, 750m, 24.III–30.III.2002, malaise trap, R. Harin’Hala, CASENT 2135889 (deposited in CASC). Paratypes: **MADAGASCAR:** 2 males, CASENT 2135801, 2136190 (CASC).

### Trichoteleia bidentata

Talamas sp. n.

urn:lsid:zoobank.org:act:BA80C498-AA3C-4AEF-9C94-B99356B4B178

urn:lsid.biosci.ohio-state.edu:osuc_concepts: 241275

Figures 20 27 73–78 Morphbank ^10^ 

#### Description.

##### Female body length:

4.64 mm (n=1). Male body length: 3.53 mm (n=1). Color of head: dark brown to black. Central keel of frons: present, ending above interantennal process. Sculpture of medial frons in female: punctate with irregularly shaped smooth area. Sculpture of medial frons in male: smooth. Number of mandibular teeth: two. Basal node on mandible: present. Sculpture of frons below median ocellus: densely punctate throughout. Sculpture of posterior vertex: densely punctate. Sculpture of gena: punctate rugose. Basiconic sensillum on A7: absent.

##### Color of mesosoma in female:

dark brown to black. Color of mesosoma in male: dark brown to black. Sculpture along posterior pronotal sulcus: rugulose. Notaulus: smooth furrow incomplete, reaching suprahumeral sulcus as row of punctures. Sculpture of medial mesoscutum: smooth and sparsely punctate posteriorly, densely punctate and transverely rugulose anteriorly. Sculpture of mesoscutellum: smooth with sparse fine punctures throughout. Postacetabular sulcus: comprised of small cells. Mesopleural carina: present. Sculpture along ventral half of prespecular sulcus: coarsely punctate. Sculpture of posterolateral mesepisternum: coarsely punctate. Sculpture of ventral surface of mesepisternum: moderately punctate. Setation of ventral metapleural area: absent. Setation of metapleural triangle: sparse. Sculpture of metapleural triangle: punctate rugose. Posterior margin of metapleuron below propodeal spiracle: straight to moderately convex. Color of legs: femora and metacoxa brown, otherwise yellow.

##### Color of metasoma in female:

black. Color of metasoma in male: dark brown to black throughout. Posterior margin of transverse sulcus on T2: strongly convex. Sublateral tergal carina on T2: absent. Microsculpture on T2: present. Microsculpture on T3: present. Microsculpture on T4: absent. Horn on T1 in female: present as a large, apically rounded protuberance. Macrosculpture of T2 in female: reticulate rugose throughout. Macrosculpture of medial T3 in female: rugulose. Macrosculpture of lateral T3 in female: reticulate rugose. Macrosculpture of medial T4 in female: absent. Macrosculpture of lateral T4 in female: longitudinally strigose. Punctation of T4 in female: sparse along midline, otherwise dense. Macrosculpture of T5 in female: longitudinally strigose laterally. Punctation of T5 in female: absent along midline, otherwise dense. Shape of T5 in female: width of posterior margin less than length. Microscupture on T6 in female: absent. Sculpture of T6 in female: smooth along midline, densely and finely punctate laterally. Macrosculpture of T2 in male: reticulate rugose throughout. Macrosculpture of medial T3 in male: weakly rugulose. Macrosculpture of lateral T3 in male: punctate rugose. Macrosculpture of T4 in male: punctate rugulose laterally. Punctation of T4 in male: sparse along midline, otherwise dense. Macrosculpture of T5 in male: weakly rugulose laterally. Punctation of T5 in male: sparse along midline, otherwise dense throughout. Sculpture of S2: coarsely punctate throughout. Prominent longitudinal median carina on S2: absent.

##### Wings:

macropterous, apex or forewing extending beyond posterior margin of T3. Color of forewing in female: hyaline with transverse infuscate band medially. Color of forewing in male: hyaline with transverse infuscate band medially. Color of hind wing: hyaline throughout. Density of setation in fore wing: uniform throughout. Density of setation in hind wing: uniform throughout. Length of R1: more than 1.5 times as long as r. M+Cu and RS+M in forewing: spectral.

**Figures 25–30.**
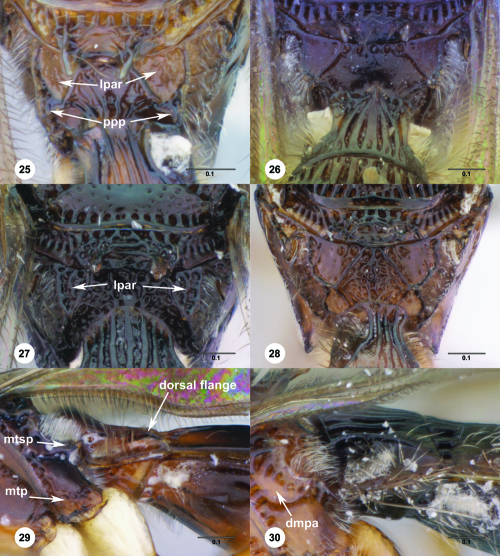
^72^ **25** Trichoteleia tahotra sp. n., Propodeum, dorsal view, female (CASENT 2043325) **26** Trichoteleia oculea sp. n., Propodeum, posterodorsal view, female (CASENT 2135907) **27** Trichoteleia bidentata sp. n., Propodeum, posterodorsal view, male (CASENT 2132802) **28** Trichoteleia tigris sp. n., Propodeum, posterodorsal view, male (CASENT 2135854) **29** Trichoteleia nify sp. n., Metapleuron and T1, lateral view, female holotype (CASENT 2135909) **30** Trichoteleia levii sp. n., Metapleuron and T1, lateral view, female (CASENT 2043789). Scale bars in millimeters.

**Figures 31–36.**
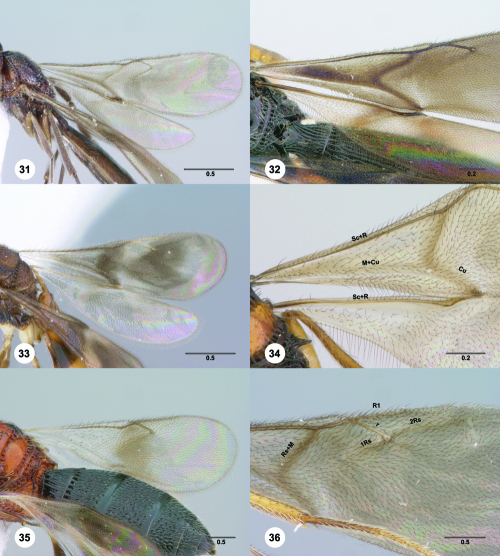
^73^ **31** Trichoteleia hemlyae sp. n., Fore wing and hind wing, dorsal view, male (CASENT 2132758) **32** Trichoteleia quazii sp. n., Fore wing and hind wing, dorsal view, female holotype (CASENT 2132026) **33** Trichoteleia picturata sp. n., Fore wing and hind wing, dorsal view, female holotype (CASENT 2135904) **34** Trichoteleia nify sp. n., Fore wing and hind wing, dorsal view, male (CASENT 2135884) **35** Trichoteleia afo sp. n., Fore wing, dorsal view, female (CASENT 2043326) **36** Trichoteleia tahotra sp. n., Fore wing, dorsal view, male (CASENT 2134186). Scale bars in millimeters.

**Figures 37–42.**
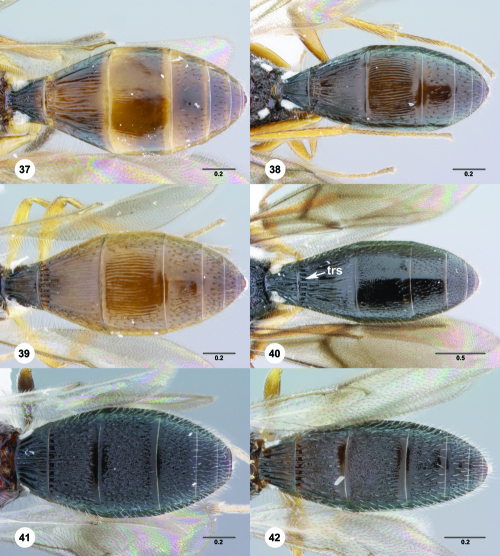
^74^ **37** Trichoteleia tigris sp. n., Metasoma, dorsal view, male (CASENT 2134160) **38** Trichoteleia janus sp. n., Metasoma, dorsal view, male (CASENT 2043786) **39** Trichoteleia rugifrons sp. n., Metasoma, dorsal view, male (CASENT 2135938) **40** Trichoteleia orona sp. n., Metasoma, dorsal view, male (CASENT 2134153) **41** Trichoteleia afo sp. n., Metasoma, dorsal view, male (CASENT 2134153) **42** Trichoteleia jiro sp. n., Metasoma, dorsal view, male (OSUC 143329). Scale bars in millimeters.

#### Diagnosis.

Bidentate mandibles are present in two known species of Trichoteleia, Trichoteleia bidentata and Trichoteleia prolixa. Trichoteleia bidentata may be separated from Trichoteleia prolixa by the coarsely sculptured, relatively narrow gena and the less extreme length of the metasoma.

#### Etymology.

The adjectival epithet *bidentata* refers to the number of mandibular teeth in this species.

#### Link to Distribution Map.

[http://hol.osu.edu/map-large.html?id=241275]

#### Material Examined.

Holotype, female: **MADAGASCAR:** Toliara Auto. Prov., 19.8km (84°) E Sakaraha, leaf mold / rotten wood / tropical dry forest, BLF7510, Zombitse-Vohibasia National Park, 22°50'36"S, 44°42'36"E, 770m, 5.II–9.II.2003, sifted litter, Fisher, Griswold et al., CASENT 2136543 (deposited in CASC). Paratype: **MADAGASCAR:** 1 male, CASENT 2132802 (CASC).

### Trichoteleia carinata

Talamas & Johnson sp. n.

urn:lsid:zoobank.org:act:D153451D-A119-4583-A406-5DFFF0FF9EFF

urn:lsid.biosci.ohio-state.edu:osuc_concepts:249011

Figures 45 79–84 Morphbank ^11^ 

#### Description.

##### Male body length:

3.35–4.05 mm (n=2). Color of head: dark brown to black. Central keel of frons: present, extending onto interantennal process; present, bifurcating ventrally around interantennal process. Sculpture of medial frons in male: smooth. Number of mandibular teeth: three. Basal node on mandible: absent. Sculpture of frons below median ocellus: moderately punctate throughout. Sculpture of posterior vertex: moderately punctate. Occipital rim: comprised of medium to large sized cells. Sculpture of gena: punctate rugose; dorsoventrally strigose.

##### Color of mesosoma in female:

dark brown to black. Sculpture along posterior pronotal sulcus: striate, striae well defined. Notaulus: percurrent, reaching suprahumeral sulcus as a smooth furrow; smooth furrow incomplete, reaching suprahumeral sulcus as row of punctures. Sculpture of medial mesoscutum: moderately punctate in posterior half, becoming denser anteriorly. Sculpture of mesoscutellum: smooth with sparse fine punctures throughout. Postacetabular sulcus: comprised of small cells. Mesopleural carina: absent. Sculpture along ventral half of prespecular sulcus: punctate. Sculpture of posterolateral mesepisternum: punctate. Sculpture of ventral surface of mesepisternum: finely punctate. Setation of ventral metapleural area: absent. Setation of metapleural triangle: sparse. Sculpture of metapleural triangle: punctate rugose. Posterior margin of metapleuron below propodeal spiracle: straight to moderately convex. Color of legs: fore and mid legs yellow to pale brown, hind legs dark brown.

##### Color of metasoma in female:

dark brown to black throughout. Posterior margin of transverse sulcus on T2: straight. Sublateral tergal carina on T2: present. Microsculpture on T2: present. Microsculpture on T3: present. Microsculpture on T4: absent. Macrosculpture of T2 in male: longitudinally striate throughout. Macrosculpture of medial T3 in male: longitudinally strigose. Macrosculpture of lateral T3 in male: longitudinally strigose. Macrosculpture of T4 in male: absent. Punctation of T4 in male: sparse in medial third, moderately dense laterally. Macrosculpture of T5 in male: absent. Punctation of T5 in male: dense throughout; sparse in medial third, dense laterally. Sculpture of S2: densely punctate, punctures of moderate size. Prominent longitudinal median carina on S2: present.

##### Wings:

Wings: macropterous, apex or forewing extending beyond posterior margin of T3. Color of forewing in male: slightly infuscate throughout. Color of hind wing: slightly infuscate throughout. Density of setation in fore wing: uniform throughout. Density of setation in hind wing: uniform throughout. Length of R1: more than 1.5 times as long as r. M+Cu and RS+M in forewing: nebulous.

**Figures 43–48.**
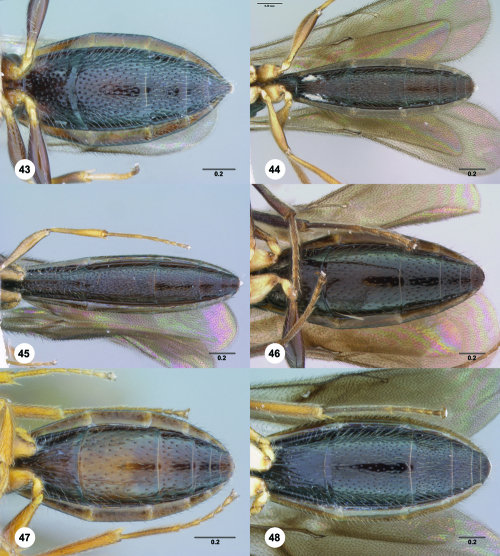
^75^ **43** Trichoteleia afo sp. n., Metasoma, ventral view, female (CASENT 2042915) **44** Trichoteleia hemlyae sp. n., Metasoma, ventral view, female (CASENT 2135808) **45** Trichoteleia carinata sp. n., Metasoma, ventral view, male (OSUC 181062) **46** Trichoteleia takariva sp. n., Metasoma, ventral view, male holotype (CASENT 2043217) **47** Trichoteleia janus sp. n., Metasoma, ventral view, male (CASENT 2043789) **48** Trichoteleia orona sp. n., Metasoma, ventral view, male (CASENT 2134153). Scale bars in millimeters.

**Figures 49–54.**
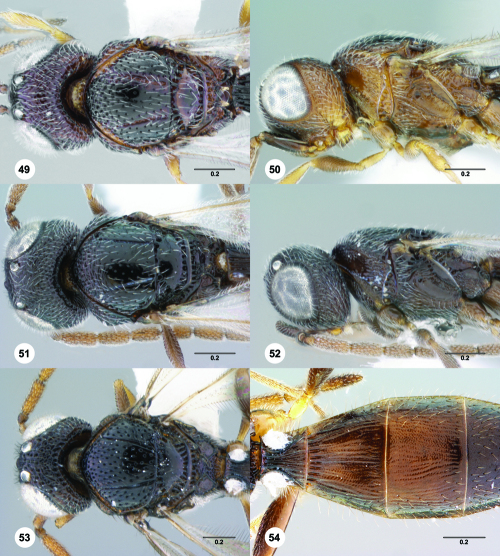
^76^ **49** Trichoteleia jiro sp. n., Head and mesosoma, dorsal view, male (OSUC 143329) **50** Trichoteleia jiro sp. n., Head and mesosoma, lateral view, male (OSUC 143329) **51** Trichoteleia bicolor sp. n., Head and mesosoma, dorsal view, male (CASENT 2135801) **52** Trichoteleia bicolor sp. n., Head and mesosoma, lateral view, male (CASENT 2135801) **53** Trichoteleia tigris sp. n., Head and mesosoma, dorsal view, male (CASENT 2135969) **54** Trichoteleia tigris sp. n., Metasoma, dorsal view, male (CASENT 2135966). Scale bars in millimeters.

**Figures 55–60.**
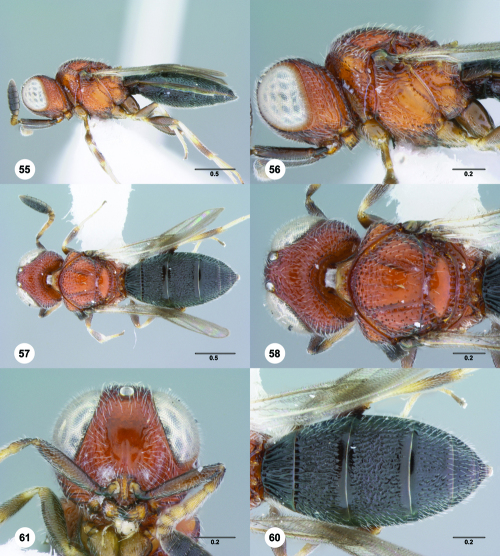
^77^ Trichoteleia afo sp. n. **55** Lateral habitus, female (OSUC 143344) **56** Head and mesosoma, lateral view, female (OSUC 143344) **57** Dorsal habitus, female (CASENT 2042728) **58** Head and mesosoma, dorsal view, female (CASENT 2042728) **59** Head, anterior view, female (CASENT 2042728) **60** Metasoma, dorsal view, female (CASENT 2042728). Scale bars in millimeters.

**Figures 61–66.**
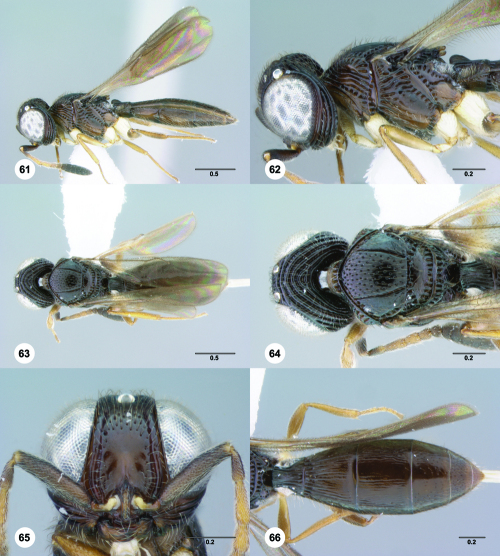
^78^ Trichoteleia albidipes Kieffer. **61** Lateral habitus, female (CASENT 2132453) **62** Head and mesosoma, lateral view, female (CASENT 2132453) **63** Dorsal habitus, female (CASENT 2132457) **64** Head and mesosoma, dorsal view, female (CASENT 2132457) **65** Head, anterior view, female (CASENT 2132453) **66** Metasoma, dorsal view, female (CASENT 2132456). Scale bars in millimeters.

**Figures 67–72.**
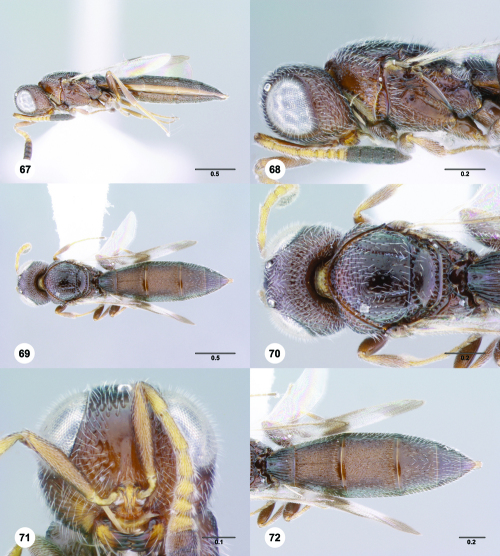
^79^Trichoteleia bicolor sp. n., female holotype (CASENT 2135889). **67** Lateral habitus **68** Head and mesosoma, lateral view **69** Dorsal habitus **70** Head and mesosoma, dorsal view **71** Head, anterior view **72** Metasoma, dorsal view. Scale bars in millimeters.

**Figures 73–78.**
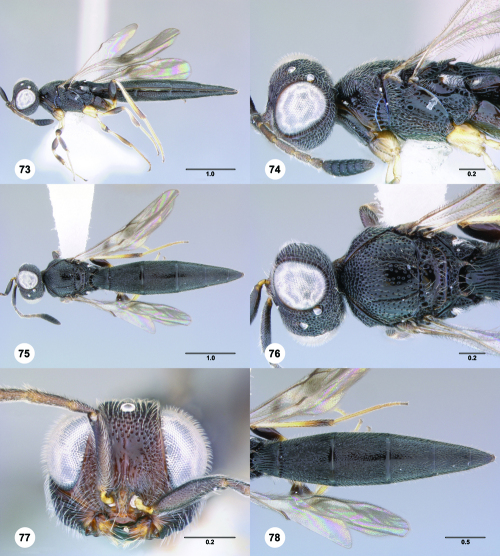
^80^Trichoteleia bidentata sp. n., female holotype (CASENT 2136543). **73** Lateral habitus **74** Head and mesosoma, lateral view **75** Dorsal habitus **76** Head and mesosoma, dorsal view **77** Head, anterior view **78** Metasoma, dorsal view. Scale bars in millimeters.

**Figures 79–84.**
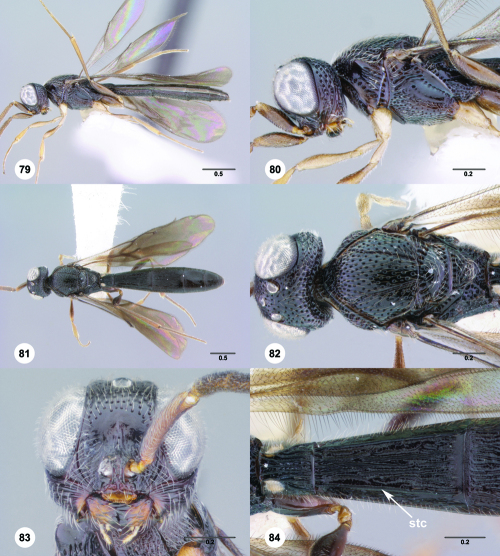
^81^Trichoteleia carinata sp. n. **79** Lateral habitus, male (OSUC 181062) **80** Head and mesosoma, lateral view, male (OSUC 181062) **81** Dorsal habitus, male holotype (OSUC 181015) **82** Head and mesosoma, dorsal view, male holotype (OSUC 181015) **83** Head, anterior view, male holotype (OSUC 181015) **84** Metasoma, dorsal view, male holotype (OSUC 181015). Scale bars in millimeters.

#### Diagnosis.

Trichoteleia carinata has a distinctly elongate metasoma similar to Trichoteleia longiventris, and it has a longitudinal median carina on S2, as does Trichoteleia quazii (Fig. 45). It can be separated from both of these species by the presence of a prominent longitudinal carina on the lateral part of T2 (Fig. 84).

#### Etymology.

This species is named for the diagnostic carinae on lateral T2 and medial S2. The epithet is an adjective.

#### Link to Distribution Map.

[http://hol.osu.edu/map-large.html?id=249011]

#### Material Examined.

Holotype, male: **MADAGASCAR:** Fianarantsoa Auto. Prov., Ranomafana National Park, 21°15'05"S, 47°24'43"E, 1190m, 21.I–31.I.2002, malaise trap, R. Harin’Hala & M. Irwin, OSUC 181015 (deposited in CASC). Other material: **MADAGASCAR:** 1 male, OSUC 181062 (CNCI).

### Trichoteleia cincta

Talamas and Masner sp. n.

urn:lsid:zoobank.org:act:EBCA57A2-BFB9-438D-B524-9BF79B608A05

urn:lsid:biosci.ohio-state.edu:osuc_concepts:238230

**Figures 85–90.**
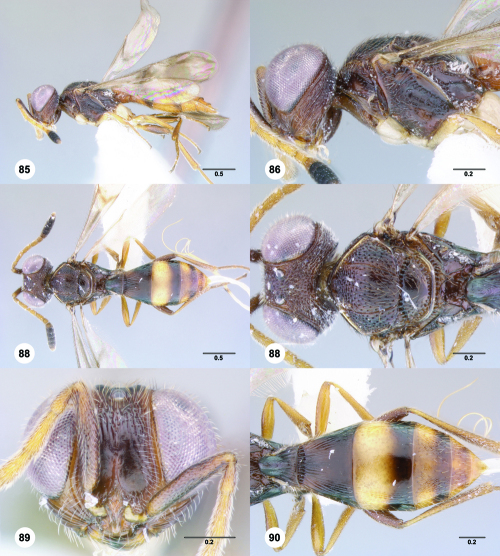
^82^Trichoteleia cincta sp. n. **85** Lateral habitus, female holotype (OSUC 181007) **86** Head and mesosoma, lateral view, female holotype (OSUC 181007) **87** Dorsal habitus, female (OSUC 181006) **88** Head and mesosoma, dorsal view, female (OSUC 181006) **89** Head, anterior view, female holotype (OSUC 81007) **90** Metasoma, dorsal view, female (OSUC 181006). Scale bars in millimeters.

Figures 85–90 Morphbank ^12^ 

#### Description.

##### Female body length:

2.93–3.01 mm (n=4). Color of head: reddish brown; yellow throughout. Central keel of frons: present, extending onto interantennal process. Sculpture of medial frons in female: smooth. Number of mandibular teeth: three. Basal node on mandible: present. Sculpture of frons below median ocellus: dorsoventrally strigose throughout. Sculpture of posterior vertex: rugulose with faint concentric tendency. Occipital rim: comprised of small to miniscule cells. Sculpture of gena: irregularly rugulose; dorsoventrally strigose. Basiconic sensillum on A7: absent.

##### Color of mesosoma in female:

reddish brown throughout; yellow throughout. Sculpture along posterior pronotal sulcus: striate, striae well defined; striate, striae short and poorly defined. Notaulus: percurrent, reaching suprahumeral sulcus as a smooth furrow. Sculpture of medial mesoscutum: longitudinally rugulose posteriorly, transversely rugulose anteriorly; moderately punctate in posterior half, becoming denser and transversely rugulose anteriorly. Sculpture of mesoscutellum: smooth medially, coarsely punctate laterally; smooth medially, densely and finely punctate laterally. Postacetabular sulcus: present as a smooth furrow. Mesopleural carina: present. Sculpture along ventral half of prespecular sulcus: punctate rugose. Sculpture of posterolateral mesepisternum: smooth. Sculpture of ventral surface of mesepisternum: smooth. Setation of ventral metapleural area: absent. Setation of metapleural triangle: sparse. Sculpture of metapleural triangle: rugulose; punctate rugose. Posterior margin of metapleuron below propodeal spiracle: with blunt kink near intersection with metapleural sulcus. Color of legs: yellow throughout; coxae and trochanters yellow, otherwise brown, becoming paler distally.

##### Color of metasoma in female:

yellow; variably patterned in alternating orange and brown. Posterior margin of transverse sulcus on T2: strongly convex. Sublateral tergal carina on T2: absent. Microsculpture on T2: absent. Microsculpture on T3: absent. Microsculpture on T4: absent. Horn on T1 in female: present as a large, apically rounded protuberance. Macrosculpture of T2 in female: longitudinally striate throughout. Macrosculpture of medial T3 in female: weakly longitudinally striate; absent. Macrosculpture of lateral T3 in female: longitudinally striate. Macrosculpture of medial T4 in female: absent. Macrosculpture of lateral T4 in female: obliquely strigose; rugulose. Punctation of T4 in female: absent along midline, otherwise moderately dense. Macrosculpture of T5 in female: longitudinally strigose laterally; weakly rugulose laterally. Punctation of T5 in female: absent along midline, otherwise moderately dense. Shape of T5 in female: width of posterior margin greater than or equal to length. Microscupture on T6 in female: absent. Sculpture of T6 in female: smooth with fine setigerous punctures along lateral margin. Sculpture of S2: longitudinally striate anteromedially, otherwise smooth. Prominent longitudinal median carina on S2: absent.

##### Wings:

macropterous, apex or forewing extending beyond posterior margin of T3. Color of forewing in female: infuscate apical to R with white spot near apex of wing. Color of hind wing: hyaline with faint infuscate patches posterior to R and along anterior margin apical to R. Density of setation in fore wing: uniform throughout. Density of setation in hind wing: uniform throughout. Length of R1: more than 1.5 times as long as r. M+Cu and RS+M in forewing: nebulous.

#### Diagnosis.

This species shares the color pattern of the wings with Trichoteleia delilah, Trichoteleia picturata, and Trichoteleia zuparkoi. It may be separated from Trichoteleia delilah by the lack of a basiconic sensillum on A7, and from Trichoteleia picturata and Trichoteleia zuparkoi by the dorsoventrally strigose sculpture below the median ocellus.

#### Etymology.

The adjectival species epithet refers to color pattern of the metasoma.

#### Link to Distribution Map.

[http://hol.osu.edu/map-large.html?id=238230]

#### Material Examined.

Holotype, female: **MADAGASCAR:** Antsiranana Auto. Prov., Marojejy National Park, 14°26.2'S 49°44.5'E, 1250m, 25.X–3.XI.1996, malaise trap, E. Quinter, OSUC 181007 (deposited in CASC). Paratypes: **MADAGASCAR:** 4 females, OSUC 181006, 181037–181038 (AMNH); OSUC 266090 (CASC).

#### Comments.

The sculpture of the medial mesoscutum and the color of the body is extremely variable in Trichoteleia cincta. The anterior half of the medial mesoscutum varies from smooth to transversely rugose and the posterior half varies from smooth to longitudinally strigose. The two specimens with more pronounced sculpture have a brown head and mesosoma with a banded metasoma (Fig. 90). Those with smoother sculpture are entirely yellow.

### Trichoteleia delilah

Talamas sp. n.

urn:lsid:zoobank.org:act:5E189039-DE79-4CAA-BB72-283863D03D6A

urn:lsid.biosci.ohio-state.edu:osuc_concepts:243413

Figures 1 17 91–96 Morphbank ^13^ 

#### Description.

Female body length: 3.21 mm (n=1). Color of head: reddish brown. Central keel of frons: present, bifurcating ventrally around interantennal process. Sculpture of medial frons in female: smooth. Number of mandibular teeth: three. Basal node on mandible: present. Sculpture of frons below median ocellus: punctate rugulose throughout. Sculpture of posterior vertex: punctate rugulose. Occipital rim: comprised of small to miniscule cells. Sculpture of gena: dorsoventrally strigose. Basiconic sensillum on A7: present.

Color of mesosoma in female: reddish brown throughout. Sculpture along posterior pronotal sulcus: striate, striae well defined. Notaulus: percurrent, reaching suprahumeral sulcus as a smooth furrow. Sculpture of medial mesoscutum: longitudinally rugulose posteriorly, transversely rugulose anteriorly. Sculpture of mesoscutellum: smooth medially, coarsely punctate laterally. Postacetabular sulcus: present as a smooth furrow. Mesopleural carina: present. Sculpture along ventral half of prespecular sulcus: punctate rugose. Sculpture of posterolateral mesepisternum: smooth except for 1 or 2 striae parallel to mesopleural carina. Sculpture of ventral surface of mesepisternum: finely punctate. Setation of ventral metapleural area: absent. Setation of metapleural triangle: sparse. Sculpture of metapleural triangle: punctate rugose. Posterior margin of metapleuron below propodeal spiracle: with blunt kink near intersection with metapleural sulcus. Color of legs: yellow throughout.

Color of metasoma in female: dark brown to black throughout. Posterior margin of transverse sulcus on T2: weakly convex. Sublateral tergal carina on T2: absent. Microsculpture on T2: present. Microsculpture on T3: present. Microsculpture on T4: present. Horn on T1 in female: present as a large, apically rounded protuberance. Macrosculpture of T2 in female: longitudinally striate throughout. Macrosculpture of medial T3 in female: weakly longitudinally strigose. Macrosculpture of lateral T3 in female: longitudinally strigose. Macrosculpture of medial T4 in female: absent. Macrosculpture of lateral T4 in female: punctate rugulose. Punctation of T4 in female: absent along midline, otherwise moderately dense. Macrosculpture of T5 in female: punctate rugulose laterally. Punctation of T5 in female: moderately dense throughout. Shape of T5 in female: width of posterior margin greater than or equal to length. Microscupture on T6 in female: absent. Sculpture of T6 in female: smooth with fine setigerous punctures along lateral margin. Sculpture of S2: longitudinally striate throughout. Prominent longitudinal median carina on S2: absent.

Wings: macropterous, apex or forewing extending beyond posterior margin of T3. Color of forewing in female: infuscate in apical two-thirds, white spot near apex, two white patches medially at anterior and posterior margins. Color of hind wing: slightly infuscate throughout. Density of setation in fore wing: uniform throughout. Density of setation in hind wing: uniform throughout. Length of R1: less than 1.5 times as long as rs. M+Cu and RS+M in forewing: nebulous.

**Figures 91–96.**
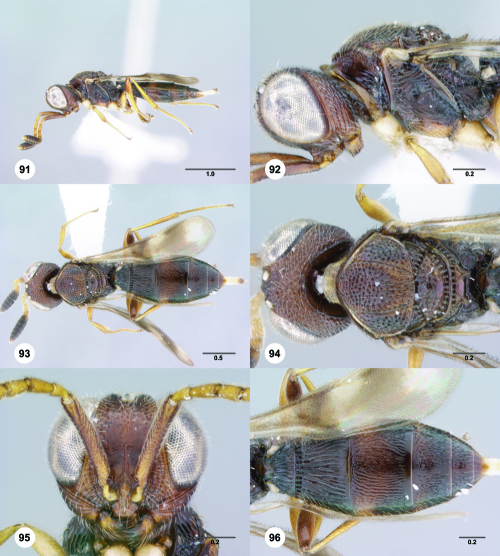
^83^Trichoteleia delilah sp. n., female holotype (CASENT 2135905). **91** Lateral habitus **92** Head and mesosoma, lateral view **93** Dorsal habitus **94** Head and mesosoma, dorsal view **95** Head, anterior view **96** Metasoma, dorsal view. Scale bars in millimeters.

#### Diagnosis.

Trichoteleia delilah is similar to Trichoteleia cincta, Trichoteleia picturata and Trichoteleia zuparokoi in the banded coloration of the wings and to Trichoteleia cincta and Trichoteleia picturata in the sculpture of the medial mesoscutum, which is transversely rugulose anteriorly and longitudinally rugulose posteriorly. It is readily separated from these three species by the presence of a basiconic sensillum on A7 (Fig. 1) and rugulose sculpture in the marginal depression of S3 (Fig. 17).

#### Etymology.

This aesthetically pleasing species is named for the Delilah of the Old Testament. The species epithet is a noun placed in apposition.

#### Link to Distribution Map.

[http://hol.osu.edu/map-large.html?id=243413]

#### Material Examined.

Holotype, female: **MADAGASCAR:** Antsiranana Auto. Prov., 20.4km (219°) SW Antanambao, leaf mold / rotten wood / rainforest, #1990, Manongarivo Special Reserve, 14°02.8'S 48°24.1'E, 1860m, 3.XI.1998, sifted litter, B. L. Fisher, Griswold et al., CASENT 2135905 (deposited in CASC).

### Trichoteleia eburata

Talamas sp. n.

urn:lsid:zoobank.org:act:81B73727-DDE9-41C2-853A-1E31B9C285A4

urn:lsid:biosci.ohio-state.edu:osuc_concepts:264100

**Figures 97–101.**
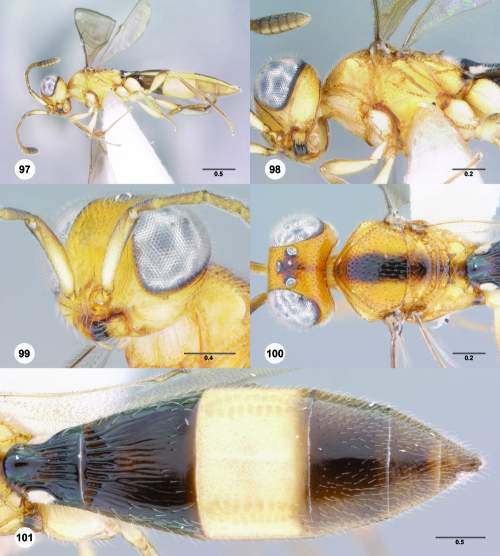
^84^Trichoteleia eburata sp. n., female holotype (CASENT 2043861). **97** Lateral habitus **98** Head and mesosoma, lateral view **99** Head, anterolateral view **100** Head and mesosoma, dorsal view **101** Metasoma, dorsal view. Scale bars in millimeters.

Figures 97–101 Morphbank ^14^ 

#### Description.

##### Color of head:

yellow throughout. Central keel of frons: present, extending onto interantennal process. Sculpture of medial frons in female: smooth. Number of mandibular teeth: three. Basal node on mandible: absent. Sculpture of frons below median ocellus: densely punctate throughout. Sculpture of posterior vertex: moderately punctate. Occipital rim: comprised of small to miniscule cells. Sculpture of gena: dorsoventrally strigose. Basiconic sensillum on A7: absent.

##### Color of mesosoma in female:

yellow with longitudinal, median brown stripe dorsally. Sculpture along posterior pronotal sulcus: striate, striae short and poorly defined. Notaulus: smooth furrow incomplete, reaching suprahumeral sulcus as row of punctures. Sculpture of medial mesoscutum: sparsely punctate, becoming denser anteriorly. Sculpture of mesoscutellum: smooth medially, sparsely punctate laterally. Mesopleural carina: present. Sculpture along ventral half of prespecular sulcus: punctate. Sculpture of posterolateral mesepisternum: punctate. Setation of ventral metapleural area: absent. Setation of metapleural triangle: moderately dense. Sculpture of metapleural triangle: densely punctate. Posterior margin of metapleuron below propodeal spiracle: straight to moderately convex. Color of legs: coxae and trochanters white, otherwise yellow to pale brown.

##### Color of metasoma in female:

T1–T2 and S1–S2 dark brown, T3 and S3 white, T4–T6 and S4–S6 light brown. Posterior margin of transverse sulcus on T2: strongly convex. Sublateral tergal carina on T2: absent. Microsculpture on T2: absent. Microsculpture on T3: absent. Microsculpture on T4: absent. Horn on T1 in female: present as a large, apically rounded protuberance. Macrosculpture of T2 in female: longitudinally strigose throughout. Macrosculpture of medial T3 in female: absent. Macrosculpture of lateral T3 in female: absent. Macrosculpture of medial T4 in female: absent. Macrosculpture of lateral T4 in female: absent. Punctation of T4 in female: sparse in medial third, moderately dense laterally. Macrosculpture of T5 in female: absent. Punctation of T5 in female: sparse along midline, otherwise dense. Microscupture on T6 in female: absent. Sculpture of T6 in female: smooth along midline, densely and finely punctate laterally. Sculpture of S2: crenulate along anterior margin, otherwise smooth with small punctures throughout. Prominent longitudinal median carina on S2: absent.

##### Wings:

macropterous, apex or forewing extending beyond posterior margin of T3. Color of forewing in female: slightly infuscate throughout. Color of hind wing: slightly infuscate throughout. Density of setation in fore wing: uniform throughout. Density of setation in hind wing: uniform throughout. Length of R1: more than 1.5 times as long as r. M+Cu and RS+M in forewing: nebulous.

#### Diagnosis.

Trichoteleia eburata is most similar to Trichoteleia hemlyae, Trichoteleia ravaka and Trichoteleia pauliani. The absence of microsculpture on the metasoma of Trichoteleia eburata differentiates it.

#### Etymology.

The adjectival epithet *eburata*, meaning “inlaid with ivory” in Latin, refers to the pale color of T3 and S3 on the metasoma of this species.

#### Link to Distribution Map.

[http://hol.osu.edu/map-large.html?id=264100]

#### Material Examined.

Holotype, female: **MAYOTTE:** litter / rainforest, BLF19060, Dapani, 12°57'46"S, 45°09'01"E, 135m, 2.XII–4.XII.2007, sifted litter, B. L. Fisher et al., CASENT 2043861 (deposited in CASC).

### Trichoteleia echinata

Talamas sp. n.

urn:lsid:zoobank.org:act:0CD0973C-4446-4698-BE73-DB6181470070

urn:lsid:biosci.ohio-state.edu:osuc_concepts:253616

**Figures 102–107.**
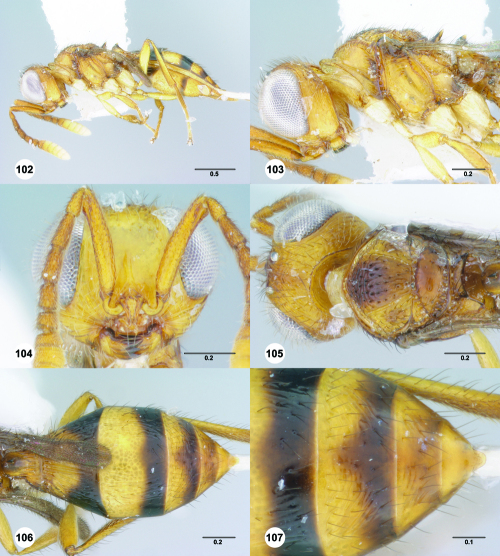
^85^Trichoteleia echinata sp. n., female holotype (CASENT 2040887). **102** Lateral habitus **103** Head and mesosoma, lateral view **104** Head, anterior view **105** Head and mesosoma, dorsal view **106** Metasoma, dorsal view **107** T4–T6, dorsal view. Scale bars in millimeters.

Figures 102–107 Morphbank ^15^ 

#### Description.

##### Color of head:

yellow, becoming darker dorsally. Central keel of frons: present, extending onto interantennal process. Sculpture of medial frons in female: smooth. Number of mandibular teeth: three. Basal node on mandible: present. Sculpture of frons below median ocellus: finely punctate throughout, dorsoventrally strigose laterally. Sculpture of posterior vertex: rugulose with faint concentric tendency. Occipital rim: comprised of small to miniscule cells. Sculpture of gena: dorsoventrally strigose. Basiconic sensillum on A7: absent.

##### Color of mesosoma in female:

variably yellow to brown. Sculpture along posterior pronotal sulcus: striate, striae well defined. Notaulus: percurrent, reaching suprahumeral sulcus as a smooth furrow. Sculpture of medial mesoscutum: longitudinally rugulose posteriorly, transversely rugulose anteriorly. Sculpture of mesoscutellum: smooth medially, sparsely punctate laterally. Postacetabular sulcus: present as a smooth furrow. Mesopleural carina: present. Sculpture along ventral half of prespecular sulcus: weakly rugulose. Sculpture of posterolateral mesepisternum: smooth. Sculpture of ventral surface of mesepisternum: smooth. Setation of ventral metapleural area: absent. Setation of metapleural triangle: sparse. Sculpture of metapleural triangle: rugulose. Posterior margin of metapleuron below propodeal spiracle: with blunt kink near intersection with metapleural sulcus. Color of legs: yellow throughout.

##### Color of metasoma in female:

variably patterned in alternating orange and brown. Posterior margin of transverse sulcus on T2: weakly convex. Sublateral tergal carina on T2: absent. Microsculpture on T2: absent. Microsculpture on T3: absent. Microsculpture on T4: absent. Horn on T1 in female: present as a large protuberance, curved posteriorly at apex. Macrosculpture of medial T3 in female: absent. Macrosculpture of lateral T3 in female: longitudinally striate. Macrosculpture of medial T4 in female: transversely strigose anteriorly, smooth posteriorly. Macrosculpture of lateral T4 in female: obliquely strigose. Punctation of T4 in female: sparse throughout. Macrosculpture of T5 in female: obliquely strigose laterally. Punctation of T5 in female: sparse throughout. Microscupture on T6 in female: absent. Sculpture of T6 in female: smooth with fine setigerous punctures along lateral margin. Sculpture of S2: longitudinally striate anteriorly, smooth posteriorly. Prominent longitudinal median carina on S2: absent.

##### Wings:

brachypterous, apex of forewing ending before midpoint of T3. Color of forewing in female: infuscate throughout. Color of hind wing: infuscate throughout. Density of setation in fore wing: uniform throughout. Density of setation in hind wing: uniform throughout. Length of R1: more than 1.5 times as long as r.

#### Diagnosis.

Trichoteleia echinata is most easily separated from the other brachypterous species, Trichoteleia parvipennis and Trichoteleia halterata, by the apically pointed horn on T1.

#### Etymology.

Trichoteleia echinata is named for the large metascutellar spines and pointed apex of the horn on T1. The epithet is used as an adjective.

#### Link to Distribution Map.

[http://hol.osu.edu/map-large.html?id=253616]

#### Material Examined.

Holotype, female: **MADAGASCAR:** Toamasina Auto. Prov., Ambanizana River, montane rainforest, BLF8649, Masoala National Park, 15°34'18"S, 50°00'22"E, 800–897m, 26.II–2.III.2003, pitfall trap, D. Andriamalala & D. Silva, CASENT 2040887 (deposited in CASC).

### Trichoteleia fisheri

Talamas & Masner sp. n.

urn:lsid:zoobank.org:act:833E1B15-2374-4DC4-81D5-CAFD62AF6309

urn:lsid.biosci.ohio-state.edu:osuc_concepts:238231

**Figures 108–113.**
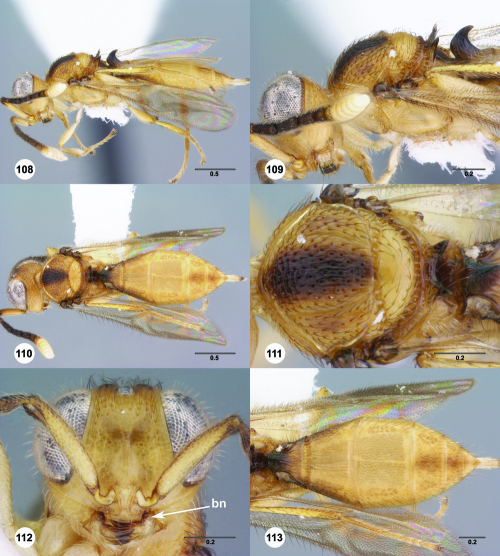
^86^Trichoteleia fisheri sp. n., female holotype (OSUC 181008). **108** Lateral habitus **109** Head and mesosoma, lateral view **110** Dorsal habitus **111** Mesosoma, dorsal view **112** Head, anterior view **113** Metasoma, dorsal view. Scale bars in millimeters.

Figures 108–113 Morphbank ^16^ 

#### Description.

##### Female body length:

2.81 mm (n=1). Color of head: yellow throughout. Central keel of frons: present, ending above interantennal process. Sculpture of medial frons in female: smooth. Number of mandibular teeth: three. Basal node on mandible: present. Sculpture of frons below median ocellus: punctate rugulose throughout. Sculpture of posterior vertex: rugulose with faint concentric tendency. Occipital rim: comprised of small to miniscule cells. Sculpture of gena: dorsoventrally strigose. Basiconic sensillum on A7: absent.

##### Color of mesosoma in female:

variably yellow to brown. Sculpture along posterior pronotal sulcus: rugulose. Notaulus: percurrent, reaching suprahumeral sulcus as a smooth furrow. Sculpture of medial mesoscutum: longitudinally rugulose posteriorly, transversely rugulose anteriorly. Sculpture of mesoscutellum: smooth medially, moderately punctate laterally. Postacetabular sulcus: comprised of small cells. Mesopleural carina: present. Sculpture along ventral half of prespecular sulcus: punctate rugose. Sculpture of posterolateral mesepisternum: smooth. Setation of ventral metapleural area: absent. Setation of metapleural triangle: sparse. Sculpture of metapleural triangle: rugulose. Posterior margin of metapleuron below propodeal spiracle: with blunt kink near intersection with metapleural sulcus. Color of legs: yellow throughout.

##### Color of metasoma in female:

horn on T1 brown, otherwise yellow. Posterior margin of transverse sulcus on T2: weakly convex. Sublateral tergal carina on T2: absent. Microsculpture on T2: present. Microsculpture on T3: present. Microsculpture on T4: present. Horn on T1 in female: present as a large protuberance, curved posteriorly at apex. Macrosculpture of T2 in female: longitudinally strigose medially, reticulate rugose laterally. Macrosculpture of medial T3 in female: rugulose. Macrosculpture of lateral T3 in female: longitudinally strigose. Macrosculpture of medial T4 in female: obliquely strigose. Macrosculpture of lateral T4 in female: obliquely strigose. Punctation of T4 in female: sparse in medial third, moderately dense laterally. Macrosculpture of T5 in female: absent along midline, otherwise obliquely strigose throughout. Punctation of T5 in female: moderately dense throughout. Shape of T5 in female: width of posterior margin greater than or equal to length. Microscupture on T6 in female: absent. Sculpture of T6 in female: smooth with fine setigerous punctures along lateral margin. Sculpture of S2: longitudinally striate throughout. Prominent longitudinal median carina on S2: absent.

##### Wings:

macropterous, apex or forewing extending beyond posterior margin of T3. Color of forewing in female: slightly infuscate throughout. Color of hind wing: slightly infuscate throughout. Density of setation in fore wing: uniform throughout. Density of setation in hind wing: uniform throughout. Length of R1: less than 1.5 times as long as rs. M+Cu and RS+M in forewing: nebulous.

#### Diagnosis.

The female of this species bears a posteriorly curving horn on T1, forming a stout hook in lateral view (Fig. 108–109). This character is shared only with Trichoteleia echinata among known species of Trichoteleia.

#### Etymology.

This species is named in honor of Brian Fisher of the California Academy of Sciences for organizing and conducting extensive insect collection in Madagascar. Most of the species treated in this revision would not be available for study without the efforts of Dr. Fisher and his colleagues.

#### Link to Distribution Map.

[http://hol.osu.edu/map-large.html?id=238231]

#### Material Examined.

Holotype, female: **MADAGASCAR:** Antsiranana Auto. Prov., #1938, Manongarivo Special Reserve, 13°59.9'S 48°25.7'E, 1175m, 20.X.1998, B. L. Fisher, OSUC 181008 (deposited in CASC).

### Trichoteleia funesta

Talamas sp. n.

urn:lsid:zoobank.org:act:8AA5288C-4A85-404A-B578-64D5C27E0766

urn:lsid:biosci.ohio-state.edu:osuc_concepts:253565

**Figures 114–119.**
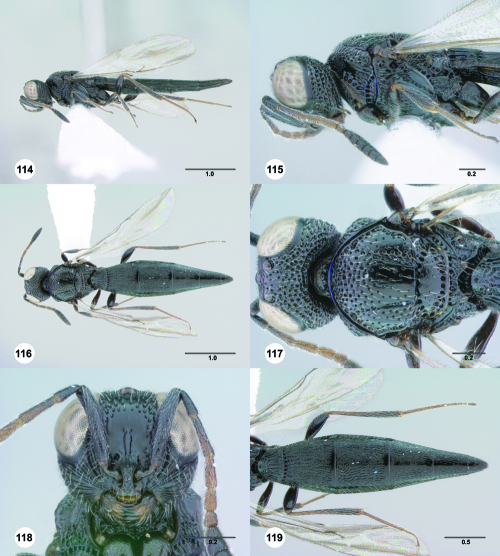
^119^ Trichoteleia funesta sp. n., female holotype (OSUC 254608). **114** Lateral habitus **115** Head and mesosoma, lateral view **116** Dorsal habitus **117** Head and mesosoma, dorsal view **118** Head, anterior view **119** Metasoma, dorsal view. Scale bars in millimeters

Figures 114–119 Morphbank ^17^ 

#### Description.

##### Color of head:

black throughout. Central keel of frons: present, bifurcating ventrally around interantennal process. Sculpture of medial frons in female: smooth. Number of mandibular teeth: three. Basal node on mandible: present. Sculpture of frons below median ocellus: punctate rugulose laterally, smooth medially. Sculpture of posterior vertex: punctate rugose. Occipital rim: comprised of medium to large sized cells. Sculpture of gena: punctate rugose. Basiconic sensillum on A7: absent.

##### Color of mesosoma in female:

dark brown to black. Sculpture along posterior pronotal sulcus: striate, striae well defined. Notaulus: smooth furrow incomplete, reaching suprahumeral sulcus as row of punctures. Sculpture of medial mesoscutum: smooth and sparsely punctate posteriorly, densely punctate and transverely rugulose anteriorly. Sculpture of mesoscutellum: smooth with few very fine punctures laterally. Mesopleural carina: present. Sculpture along ventral half of prespecular sulcus: coarsely punctate. Sculpture of posterolateral mesepisternum: coarsely punctate. Setation of ventral metapleural area: absent. Setation of metapleural triangle: sparse. Sculpture of metapleural triangle: punctate rugose. Posterior margin of metapleuron below propodeal spiracle: straight to moderately convex. Color of legs: brown throughout.

##### Color of metasoma in female:

black. Posterior margin of transverse sulcus on T2: weakly convex. Sublateral tergal carina on T2: absent. Microsculpture on T2: present. Microsculpture on T3: present. Microsculpture on T4: present. Horn on T1 in female: present as a large, apically rounded protuberance. Macrosculpture of T2 in female: longitudinally strigose throughout. Macrosculpture of medial T3 in female: weakly longitudinally strigose. Macrosculpture of lateral T3 in female: longitudinally strigose. Macrosculpture of medial T4 in female: absent. Macrosculpture of lateral T4 in female: longitudinally strigose. Punctation of T4 in female: sparse in medial third, moderately dense laterally. Macrosculpture of T5 in female: longitudinally strigose laterally. Punctation of T5 in female: moderately dense throughout. Shape of T5 in female: width of posterior margin less than length. Microscupture on T6 in female: absent. Sculpture of T6 in female: smooth with sparse fine setigerous punctures throughout. Sculpture of S2: coarsely punctate throughout. Prominent longitudinal median carina on S2: absent.

##### Wings:

macropterous, apex or forewing extending beyond posterior margin of T3. Color of forewing in female: hyaline throughout. Color of hind wing: hyaline throughout. Density of setation in fore wing: uniform throughout. Density of setation in hind wing: uniform throughout. Length of R1: more than 1.5 times as long as r. M+Cu and RS+M in forewing: spectral.

#### Diagnosis.

Trichoteleia funesta is most similar to Trichoteleia tezitra in both habitus and characters. The longitudinal strigae on T4–T5 of Trichoteleia funesta serve to separate Trichoteleia funesta.

#### Etymology.

The adjectival epithet *funesta*, meaning “mournful”, refers to the black color of this species.

#### Link to Distribution Map.

[http://hol.osu.edu/map-large.html?id=253565]

#### Material Examined.

Holotype, female: **MADAGASCAR:** Fianarantsoa Auto. Prov., 35km SSE Antsirabe, Uapacca forest, MA-24-32, Italaviana, 20°10.40'S 47°05.16'E, 1360m, 16.X–26.X.2003, malaise trap, R. Harin’Hala, OSUC 254608 (deposited in CASC).

### Trichoteleia halterata

Talamas & Masner sp. n.

urn:lsid:zoobank.org:act:8387E913-6686-4C22-8A4B-43FD20D63096

urn:lsid.biosci.ohio-state.edu:osuc_concepts:238233

**Figures 120–125.**
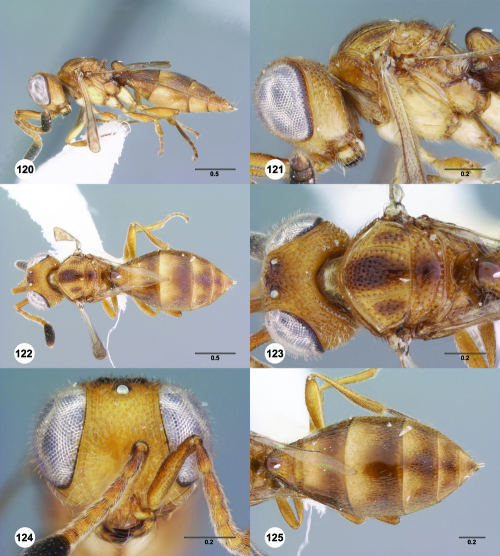
^87^Trichoteleia halterata sp. n., female holotype (OSUC 181012). **120** Lateral habitus **121** Head and mesosoma, lateral view **122** Dorsal habitus **123** Head and mesosoma, dorsal view **124** Head, anterior view **125** Metasoma, dorsal view. Scale bars in millimeters.

Figures 120–125 Morphbank ^18^ 

#### Description.

##### Female body length:

2.76 mm (n=1). Color of head: yellow, becoming darker dorsally. Central keel of frons: present, extending onto interantennal process. Sculpture of medial frons in female: smooth. Number of mandibular teeth: three. Basal node on mandible: absent. Sculpture of frons below median ocellus: punctate rugulose throughout. Sculpture of posterior vertex: rugulose with faint concentric tendency. Occipital rim: comprised of small to miniscule cells. Sculpture of gena: smooth ventrally, rugulose dorsally. Basiconic sensillum on A7: absent.

##### Color of mesosoma in female:

variably yellow to brown. Sculpture along posterior pronotal sulcus: striate, striae well defined. Notaulus: percurrent, reaching suprahumeral sulcus as a smooth furrow. Sculpture of medial mesoscutum: densely punctate throughout. Sculpture of mesoscutellum: smooth medially, moderately punctate laterally. Postacetabular sulcus: present as a smooth furrow. Mesopleural carina: present. Sculpture along ventral half of prespecular sulcus: weakly rugulose. Sculpture of posterolateral mesepisternum: smooth except for 1 or 2 striae parallel to mesopleural carina. Sculpture of ventral surface of mesepisternum: smooth. Setation of ventral metapleural area: absent. Setation of metapleural triangle: sparse. Sculpture of metapleural triangle: rugulose. Posterior margin of metapleuron below propodeal spiracle: with blunt kink near intersection with metapleural sulcus. Color of legs: coxae and trochanters yellow, otherwise pale brown, hindlegs the darkest.

##### Color of metasoma in female:

patterned in alternating yellow and pale brown. Posterior margin of transverse sulcus on T2: strongly convex. Sublateral tergal carina on T2: absent. Microsculpture on T2: absent. Microsculpture on T3: absent. Microsculpture on T4: absent. Horn on T1 in female: present as a large, apically rounded protuberance. Macrosculpture of T2 in female: longitudinally striate, medial striae not reaching posterior margin. Macrosculpture of medial T3 in female: absent. Macrosculpture of lateral T3 in female: longitudinally strigose. Macrosculpture of medial T4 in female: obliquely strigose. Macrosculpture of lateral T4 in female: obliquely strigose. Punctation of T4 in female: sparse along midline, otherwise dense. Macrosculpture of T5 in female: absent along midline, otherwise obliquely strigose throughout. Punctation of T5 in female: moderately dense throughout. Shape of T5 in female: width of posterior margin greater than or equal to length. Microscupture on T6 in female: absent. Sculpture of T6 in female: smooth with fine setigerous punctures laterally. Sculpture of S2: longitudinally striate anteromedially, otherwise smooth. Prominent longitudinal median carina on S2: absent.

##### Wings:

brachypterous, apex of forewing ending before midpoint of T3. Color of forewing in female: slightly infuscate throughout. Color of hind wing: slightly infuscate throughout. Density of setation in fore wing: uniform throughout. Density of setation in hind wing: uniform throughout. Length of R1: more than 1.5 times as long as r.

#### Diagnosis.

Among the species of Trichoteleia only Trichoteleia halterata, Trichoteleia echinata and Trichoteleia parvipennis are known to be brachypterous. Trichoteleia halterata lacks a basiconic sensillum on A7 and has a simple horn on T1. A basiconic sensillum is present on A7 in Trichoteleia parvipennis, and Trichoteleia echinata has a posterodorsally pointing spine on the horn of T1.

#### Etymology.

The adjectival epithet refers to the vaguely dumbbell shaped forewings.

#### Link to Distribution Map.

[http://hol.osu.edu/map-large.html?id=238233]

#### Material Examined.

Holotype, female: **MADAGASCAR:** Fianarantsoa Auto. Prov., rainforest, 36km S Ambalavao, 22°12'S 46°58'E, 23.X.1993, Berlese funnel, B. L. Fisher, OSUC 181012 (deposited in CASC).

### Trichoteleia hemlyae

Talamas & Masner sp. n.

urn:lsid:zoobank.org:act:A8E68218-7683-45D8-A71B-4E0662F53BC9

urn:lsid.biosci.ohio-state.edu:osuc_concepts:241293

Figures 3 9 31 44 126–131 309–310 Morphbank ^19^ 

#### Description.

##### Female body length:

2.66–3.47 mm (n=12). Male body length: 2.24–3.11 mm (n=22). Color of head: dark orange, becoming brown at vertex; orange throughout; dark brown to black; yellow throughout. Central keel of frons: present, extending onto interantennal process. Sculpture of medial frons in female: smooth. Sculpture of medial frons in male: smooth. Number of mandibular teeth: three. Basal node on mandible: absent. Sculpture of frons below median ocellus: densely punctate throughout. Sculpture of posterior vertex: moderately punctate; densely punctate. Occipital rim: comprised of small to miniscule cells. Sculpture of gena: punctate rugose; dorsoventrally strigose. Basiconic sensillum on A7: absent.

##### Color of mesosoma in female:

dark brown to black; variably orange to brown. Color of mesosoma in male: dark brown to black. Sculpture along posterior pronotal sulcus: striate, striae well defined. Notaulus: smooth furrow incomplete, reaching suprahumeral sulcus as row of punctures. Sculpture of medial mesoscutum: moderately punctate in posterior half, becoming denser anteriorly; moderately punctate in posterior half, becoming denser and transversely rugulose anteriorly. Sculpture of mesoscutellum: smooth with sparse fine punctures throughout; smooth medially, sparsely punctate laterally. Postacetabular sulcus: comprised of small cells. Mesopleural carina: present. Sculpture along ventral half of prespecular sulcus: punctate. Sculpture of posterolateral mesepisternum: punctate; sparsely punctate. Sculpture of ventral surface of mesepisternum: finely punctate. Setation of ventral metapleural area: absent. Setation of metapleural triangle: sparse; moderately dense. Sculpture of metapleural triangle: punctate rugose; densely punctate. Posterior margin of metapleuron below propodeal spiracle: straight to moderately convex; with blunt kink near intersection with metapleural sulcus. Color of legs: pale brown throughout; brown throughout; fore coxa and trochanter yellow, otherwise brown.

##### Color of metasoma in female:

dark brown to black throughout. Color of metasoma in male: pale to dark brown throughout. Posterior margin of transverse sulcus on T2: strongly convex. Sublateral tergal carina on T2: absent. Microsculpture on T2: present. Microsculpture on T3: present. Microsculpture on T4: present. Horn on T1 in female: present as a large, apically rounded protuberance. Macrosculpture of T2 in female: weakly longitudinally striate throughout; striate anteriorly, with few striae reaching T3. Macrosculpture of medial T3 in female: absent; weakly longitudinally strigose. Macrosculpture of lateral T3 in female: absent; longitudinally strigose. Macrosculpture of medial T4 in female: absent. Macrosculpture of lateral T4 in female: absent. Punctation of T4 in female: sparse in medial third, moderately dense laterally; absent in medial third, sparse laterally; sparse throughout. Macrosculpture of T5 in female: absent. Punctation of T5 in female: moderately dense throughout; sparse along midline, otherwise dense. Shape of T5 in female: width of posterior margin less than length. Microscupture on T6 in female: absent. Sculpture of T6 in female: smooth along midline, densely and finely punctate laterally. Macrosculpture of T2 in male: striate anteriorly, with few striae reaching T3; weakly longitudinally striate throughout. Macrosculpture of medial T3 in male: absent. Macrosculpture of lateral T3 in male: absent. Macrosculpture of T4 in male: absent. Punctation of T4 in male: sparse throughout; absent in medial third, sparse laterally. Macrosculpture of T5 in male: absent. Punctation of T5 in male: sparse along midline, otherwise dense throughout; sparse in medial third, moderately dense laterally. Sculpture of S2: longitudinally striate medially, punctate interstitially. Prominent longitudinal median carina on S2: absent.

##### Wings:

macropterous, apex of forewing extending beyond posterior margin of T3. Color of forewing in female: slightly infuscate with longitudinal dark streak. Color of forewing in male: slightly infuscate throughout; slightly infuscate with longitudinal dark streak. Color of hind wing: slightly infuscate throughout; hyaline with faint infuscate patches posterior to R and along anterior margin apical to R. Density of setation in fore wing: uniform throughout. Density of setation in hind wing: uniform throughout. Length of R1: more than 1.5 times as long as r. M+Cu and RS+M in forewing: nebulous.

**Figures 126–131.**
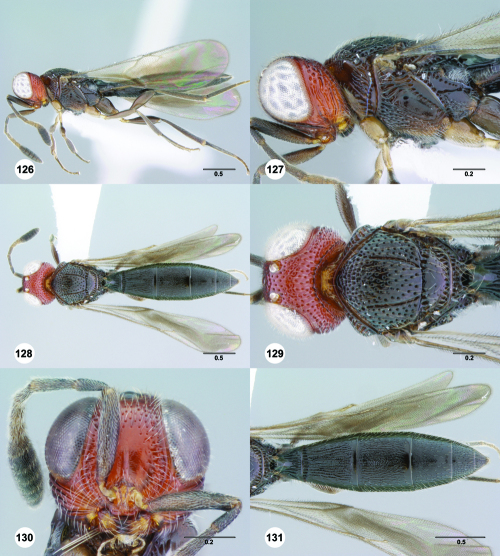
^88^Trichoteleia hemlyae sp. n. **126** Lateral habitus, female holotype (CASENT 2132833) **127** Head and mesosoma, lateral view, female holotype (CASENT 2132833) **128** Dorsal habitus, female holotype (CASENT 2132833) **129** Head and mesosoma, dorsal view, female holotype (CASENT 2132833) **130** Head, anterior view, female (CASENT 2043942) **131** Metasoma, dorsal view, female holotype (CASENT 2132833). Scale bars in millimeters.

#### Diagnosis.

Trichoteleia hemlyae is most similar to Trichoteleia pauliani and Trichoteleia ravaka. The central keel on the frons and coarsely sculptured metapleural triangle will serve to separate this species from Trichoteleia pauliani. The primary difference between Trichoteleia hemlyae and Trichoteleia ravaka is the color pattern in the forewing. Trichoteleia hemlyae has forewings that are faintly infuscate throughout with a nebulous darker streak along the longitudinal midline (Figs 31, 126, 128, 131). The forewings of Trichoteleia ravaka have infuscation in patch near the apex of the wing and in a transverse band intersecting R (marginal vein) (Fig. 231).

#### Etymology.

This species is named to honor the work and pleasant demeanor of Sara Hemly (OSU, Columbus), whose meticulous processing of specimens greatly contributed to this revision.

#### Link to Distribution Map.

[http://hol.osu.edu/map-large.html?id=241293]

#### Material Examined.

Holotype, female: **MADAGASCAR:** Fianarantsoa Auto. Prov., radio tower, forest edge / mixed tropical forest, MA-02-09B-54, Ranomafana National Park, 21°15.05'S 47°24.43'E, 1130m, 27.II–9.III.2003, malaise trap, R. Harin’Hala, CASENT 2132833 (deposited in CASC). Paratypes: **MADAGASCAR:** 18 females, 68 males, CASENT 2008625, 2008631, 2042872, 2043435, 2043487–2043488, 2043528, 2043942, 2118406, 2118409, 2118415–2118416, 2118423, 2132655, 2132725–2132726, 2132757, 2132834, 2132843, 2133932–2133933, 2134084–2134085, 2134089–2134090, 2134092, 2134162, 2134174, 2134177, 2134182, 2135732, 2135791, 2135808, 2135828, 2135832, 2135835, 2135841, 2135867–2135868, 2135881, 2135888, 2135891–2135892, 2135894–2135896, 2135899–2135900, 2135959, 2135961–2135962, 2135971, 2136001, 2136017, 2136019, 2136166, 2136179, 2136274, 2138045, 2138092, OSUC 181024, OSUC 181025, OSUC 181026, OSUC 266087 (CASC); CASENT 2043529, OSUC 181027 (CNCI); CASENT 2008627, 2042964–2042965, 2043525, 2118407, 2118414, 2132758, 2134100, 2134163, 2135810, 2135829, 2135846, 2135857, 2135875, 2135893, 2136020, 2136156, 2136158, 2136188, 2136194 (OSUC). Other material: **MADAGASCAR:** 2 females, OSUC 334219–334220 (OSUC).

### Trichoteleia irwini

Talamas & Masner sp. n.

urn:lsid:zoobank.org:act:5F1676DB-47EC-422A-9898-F32F84A1AAC7

urn:lsid.biosci.ohio-state.edu:osuc_concepts:238234

**Figures 132–137.**
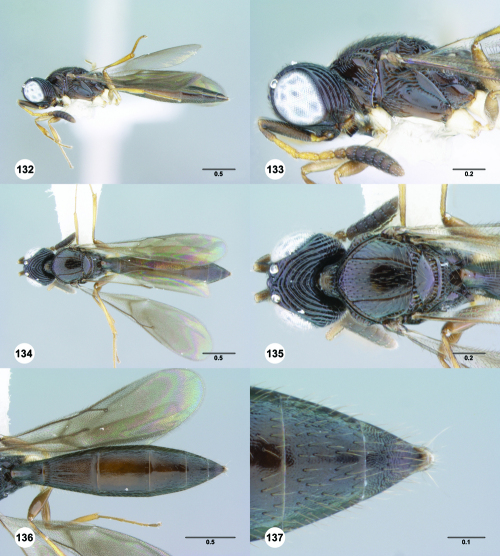
^89^Trichoteleia irwini sp. n., female holotype (CASENT 2134796). **132** Lateral habitus **133** Head and mesosoma, lateral view **134** Dorsal habitus **135** Head and mesosoma, dorsal view **136** Metasoma, dorsal view **137** T5–T6, dorsal view. Scale bars in millimeters.

Figures 132–137 Morphbank ^20^ 

#### Description.

##### Female body length:

3.31–3.90 mm (n=2). Male body length: 3.20–3.20 mm (n=2). Color of head: dark brown to black. Central keel of frons: present, ending above interantennal process. Sculpture of medial frons in female: smooth. Sculpture of medial frons in male: smooth. Number of mandibular teeth: three. Basal node on mandible: present. Sculpture of frons below median ocellus: punctate throughout with a groove parallel to orbital carina; densely punctate throughout. Sculpture of posterior vertex: concentrically rugose. Sculpture of gena: coarsely striate. Basiconic sensillum on A7: present.

##### Color of mesosoma in female:

dark brown to black. Color of mesosoma in male: dark brown to black. Sculpture along posterior pronotal sulcus: striate, striae well defined. Notaulus: percurrent, reaching suprahumeral sulcus as a smooth furrow. Sculpture of medial mesoscutum: moderately punctate in posterior half, becoming denser anteriorly. Sculpture of mesoscutellum: smooth with sparse fine punctures throughout; smooth medially, sparsely punctate laterally. Postacetabular sulcus: comprised of small cells. Mesopleural carina: present; present only in anterior half. Sculpture along ventral half of prespecular sulcus: punctate. Sculpture of posterolateral mesepisternum: smooth. Sculpture of ventral surface of mesepisternum: smooth. Setation of ventral metapleural area: absent. Setation of metapleural triangle: sparse. Sculpture of metapleural triangle: rugulose; finely punctate. Posterior margin of metapleuron below propodeal spiracle: with blunt kink near intersection with metapleural sulcus. Color of legs: coxae and trochanters yellow, otherwise pale brown, hindlegs the darkest.

##### Color of metasoma in female:

dark brown to black throughout. Color of metasoma in male: dark brown to black throughout. Posterior margin of transverse sulcus on T2: strongly convex. Sublateral tergal carina on T2: absent. Microsculpture on T2: present. Microsculpture on T3: present. Microsculpture on T4: present. Horn on T1 in female: present as a large, apically rounded protuberance. Macrosculpture of T2 in female: striate anteriorly, with few striae reaching T3. Macrosculpture of medial T3 in female: absent. Macrosculpture of lateral T3 in female: present as 1 or 2 strigae along junction of dorsal and lateral surfaces. Macrosculpture of medial T4 in female: absent. Macrosculpture of lateral T4 in female: present as 1 or 2 strigae along junction of dorsal and lateral surfaces. Punctation of T4 in female: absent along midline, otherwise sparse. Macrosculpture of T5 in female: weakly rugulose laterally. Punctation of T5 in female: sparse throughout. Shape of T5 in female: width of posterior margin less than length. Microscupture on T6 in female: present throughout. Sculpture of T6 in female: finely punctate throughout. Macrosculpture of T2 in male: striate anteriorly, with few striae reaching T3. Macrosculpture of medial T3 in male: absent. Macrosculpture of lateral T3 in male: present as 1 or 2 strigae along junction of dorsal and lateral surfaces. Macrosculpture of T4 in male: absent. Punctation of T4 in male: sparse throughout; absent in medial third, sparse laterally. Macrosculpture of T5 in male: absent. Punctation of T5 in male: sparsely present throughout. Sculpture of S2: longitudinally striate anteriorly, smooth posteriorly. Prominent longitudinal median carina on S2: absent.

##### Wings:

macropterous, apex or forewing extending beyond posterior margin of T3. Color of forewing in female: slightly infuscate throughout. Color of forewing in male: slightly infuscate throughout. Color of hind wing: hyaline, slightly infuscate at tip. Density of setation in fore wing: uniform throughout. Density of setation in hind wing: uniform throughout. Length of R1: more than 1.5 times as long as r. M+Cu and RS+M in forewing: nebulous.

#### Diagnosis.

Trichoteleia irwini shares with Trichoteleia albidipes and Trichoteleia ketrona the presence of large concentric rugae on the posterior vertex that are not interrupted by coarse punctures (Figs 64, 135, 153). Trichoteleia albidipes has striae that reach from the posterior margin of the lateral pronotum anteriorly to the mesoscutal suprahumeral sulcus (Fig. 62), while the striae of the pronotum in Trichoteleia irwini are clearly separated from the mesoscutal suprahumeral sulcus by a smooth area (Fig. 133). Trichoteleia irwini is separated from Trichoteleia ketrona by the presence of dense microsculpture through the metasoma and the weakly striate T2 (Fig. 136). Additionally, females of Trichoteleia irwini have a basiconic sensillum on A7 which is not found in the other two species.

#### Etymology.

Trichoteleia irwini is named after Dr. Michael Irwin for his excellent field work in Madagascar.

#### Link to Distribution Map.

[http://hol.osu.edu/map-large.html?id=238234]

#### Material Examined.

Holotype, female: **MADAGASCAR:** Fianarantsoa Auto. Prov., Belle Vue trail, Ranomafana National Park, 21°15.99'S 47°25.21'E, 1150m, 8.XII–20.XII.1999, M. E. Irwin, OSUC 181013 (deposited in CASC). Paratypes: **MADAGASCAR:** 1 female, 3 males, CASENT 2136023–2136024, 8106455 (CASC); CASENT 2134796 (OSUC).

### Trichoteleia janus

Talamas & Masner sp. n.

urn:lsid:zoobank.org:act:ADDC7428-2F9A-4DC7-B4CF-BA18BA9AA4B9

urn:lsid.biosci.ohio-state.edu:osuc_concepts:241287

Figures 6 8 14 23 38 47 138–143 Morphbank ^21^ 

#### Description.

##### Female body length:

2.26–2.69 mm (n=4). Male body length: 2.41–2.73 mm (n=4). Color of head: dark brown to black; reddish brown. Central keel of frons: present, extending onto interantennal process. Sculpture of medial frons in female: smooth. Sculpture of medial frons in male: smooth. Number of mandibular teeth: three. Basal node on mandible: present. Sculpture of frons below median ocellus: finely punctate throughout, dorsoventrally strigose laterally; finely punctate throughout, surface uneven. Sculpture of posterior vertex: moderately punctate, rugose posterior to eyes and posterior ocellus. Occipital rim: comprised of medium to large sized cells. Sculpture of gena: dorsoventrally strigose. Basiconic sensillum on A7: absent.

##### Color of mesosoma in female:

variably red to black. Color of mesosoma in male: variably red to black. Sculpture along posterior pronotal sulcus: striate, striae short and poorly defined; rugulose. Notaulus: percurrent, reaching suprahumeral sulcus as a smooth furrow. Sculpture of medial mesoscutum: moderately punctate in posterior half, becoming denser anteriorly. Sculpture of mesoscutellum: smooth medially, sparsely punctate laterally. Postacetabular sulcus: comprised of small cells. Mesopleural carina: present. Sculpture along ventral half of prespecular sulcus: punctate rugose. Sculpture of posterolateral mesepisternum: smooth. Sculpture of ventral surface of mesepisternum: smooth. Setation of ventral metapleural area: absent. Setation of metapleural triangle: sparse. Sculpture of metapleural triangle: rugulose. Posterior margin of metapleuron below propodeal spiracle: straight to moderately convex; with blunt kink near intersection with metapleural sulcus. Color of legs: pale brown throughout; brown throughout.

##### Color of metasoma in female:

dark brown to black throughout. Color of metasoma in male: dark brown to black throughout. Posterior margin of transverse sulcus on T2: strongly convex. Sublateral tergal carina on T2: absent. Microsculpture on T2: present. Microsculpture on T3: absent; present. Microsculpture on T4: absent. Horn on T1 in female: absent; present as a large, apically rounded protuberance. Macrosculpture of T2 in female: longitudinally striate throughout. Macrosculpture of medial T3 in female: weakly longitudinally striate; longitudinally striate. Macrosculpture of lateral T3 in female: longitudinally striate. Macrosculpture of medial T4 in female: absent. Macrosculpture of lateral T4 in female: weakly longitudinally strigose. Punctation of T4 in female: sparse throughout; absent along midline, otherwise sparse; absent along midline, otherwise moderately dense. Macrosculpture of T5 in female: absent. Punctation of T5 in female: moderately dense throughout; sparse throughout. Shape of T5 in female: width of posterior margin greater than or equal to length. Microscupture on T6 in female: absent. Sculpture of T6 in female: smooth with fine setigerous punctures laterally. Macrosculpture of T2 in male: longitudinally striate throughout. Macrosculpture of medial T3 in male: longitudinally striate. Macrosculpture of lateral T3 in male: longitudinally striate. Macrosculpture of T4 in male: longitudinally strigose laterally. Punctation of T4 in male: sparse throughout; absent along midline, otherwise sparse. Macrosculpture of T5 in male: absent. Punctation of T5 in male: moderately dense throughout. Sculpture of S2: longitudinally striate throughout; longitudinally striate anteriorly, smooth posteriorly. Prominent longitudinal median carina on S2: absent.

##### Wings:

macropterous, apex or forewing extending beyond posterior margin of T3. Color of forewing in female: infuscate throughout; slightly infuscate throughout. Color of forewing in male: slightly infuscate throughout. Color of hind wing: infuscate throughout; slightly infuscate throughout. Density of setation in fore wing: uniform throughout. Density of setation in hind wing: reduced posterior to Sc+R. Length of R1: more than 1.5 times as long as r. M+Cu and RS+M in forewing: nebulous.

**Figures 138–143.**
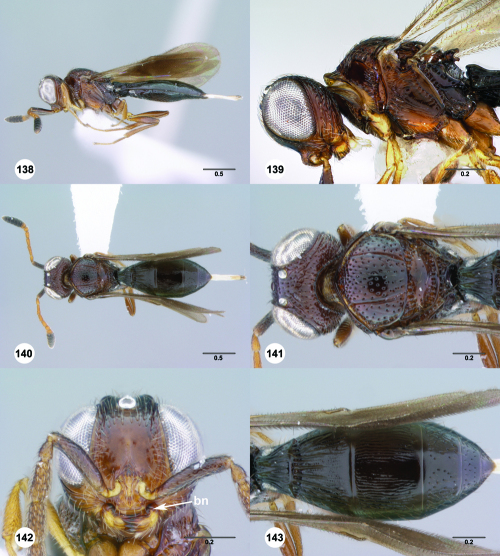
90138, Trichoteleia janus sp. n. Lateral habitus, female (CASENT 2042242) **139** Head and mesosoma, lateral view, female holotype (CASENT2135979) **140** Dorsal habitus, female (CASENT 2042242) **141** Head and mesosoma, dorsal view, female (CASENT 2042242) **142** Head, anterior view, female (CASENT 2043786) **143** Metasoma, dorsal view, female (CASENT 2042242). Scale bars in millimeters.

#### Diagnosis.

This species shares its characters with many others in Trichoteleia and is identified more by its unique combination of states than by a single distinct character. The abrupt transition from punctate rugose to smooth sculpture along the anterior mesepisternum, a smooth T6, the mostly smooth sculpture of T4–T5, and mandibular node help to identify this species.

#### Etymology.

The epithet *janus*, referring to the Roman god of gates and doors, is an allusion to the juxtaposition of sculptural types on the anterior mesepisternum. This is a noun in apposition.

#### Link to Distribution Map.

[http://hol.osu.edu/map-large.html?id=241287]

#### Material Examined.

Holotype, female: **MADAGASCAR:** Antsiranana Auto. Prov., MA-01-01A-07, Montagne d’Ambre National Park, 12°30'52"S, 49°10'53"E, 960m, 12.II–4.III.2001, malaise trap, R. Harin’Hala, CASENT 2135979 (deposited in CASC). Paratypes: **MADAGASCAR:** 4 females, 4 males, CASENT 2042242, 2043773, 2043786, 2043794, 2136576 (CASC); CASENT 2135787, 2136176, OSUC 215576 (OSUC).

### Trichoteleia jiro

Talamas, sp. n.

urn:lsid:zoobank.org:act:3DADAE94-4FAD-4A53-B00B-9C153FAD9E8A

urn:lsid.biosci.ohio-state.edu:osuc_concepts: 241279

Figures 42 49–50 144–149 306 Morphbank ^22^ 

#### Description.

##### Female body length:

2.35–2.49 mm (n=3). Male body length: 2.32–2.44 mm (n=7). Color of head: orange throughout; pale brown throughout. Central keel of frons: present, ending above interantennal process. Sculpture of medial frons in female: smooth. Sculpture of medial frons in male: smooth. Number of mandibular teeth: three. Basal node on mandible: absent. Sculpture of frons below median ocellus: punctate rugulose throughout; densely punctate throughout. Sculpture of posterior vertex: densely punctate; punctate rugose. Occipital rim: comprised of small to miniscule cells. Sculpture of gena: punctate rugose. Basiconic sensillum on A7: absent.

##### Color of mesosoma in female:

orange throughout. Color of mesosoma in male: pale brown throughout. Sculpture along posterior pronotal sulcus: rugulose. Notaulus: smooth furrow incomplete, reaching suprahumeral sulcus as row of punctures. Sculpture of medial mesoscutum: smooth and sparsely punctate posteriorly, densely punctate and transverely rugulose anteriorly; moderately punctate in posterior half, becoming denser anteriorly. Sculpture of mesoscutellum: smooth with sparse fine punctures throughout; smooth medially, sparsely punctate laterally. Postacetabular sulcus: comprised of small cells. Mesopleural carina: present. Sculpture along ventral half of prespecular sulcus: punctate; coarsely punctate. Sculpture of posterolateral mesepisternum: punctate; coarsely punctate. Sculpture of ventral surface of mesepisternum: finely punctate; smooth. Setation of ventral metapleural area: absent. Setation of metapleural triangle: dense. Sculpture of metapleural triangle: punctate rugose. Posterior margin of metapleuron below propodeal spiracle: with blunt kink near intersection with metapleural sulcus. Color of legs: yellow throughout; pale brown throughout.

##### Color of metasoma in female:

dark brown to black throughout. Color of metasoma in male: dark brown to black throughout. Posterior margin of transverse sulcus on T2: straight. Sublateral tergal carina on T2: absent. Microsculpture on T2: present. Microsculpture on T3: present. Microsculpture on T4: present. Horn on T1 in female: absent. Macrosculpture of T2 in female: longitudinally strigose throughout. Macrosculpture of medial T3 in female: reticulate. Macrosculpture of lateral T3 in female: longitudinally strigose. Macrosculpture of medial T4 in female: longitudinally strigose; weakly rugulose. Macrosculpture of lateral T4 in female: longitudinally strigose. Punctation of T4 in female: sparse throughout. Macrosculpture of T5 in female: absent. Punctation of T5 in female: moderately dense throughout. Shape of T5 in female: width of posterior margin greater than or equal to length. Microscupture on T6 in female: absent. Sculpture of T6 in female: moderately punctate throughout. Macrosculpture of T2 in male: longitudinally strigose. Macrosculpture of medial T3 in male: reticulate. Macrosculpture of lateral T3 in male: longitudinally strigose. Macrosculpture of T4 in male: longitudinally strigose throughout. Punctation of T4 in male: sparse throughout. Macrosculpture of T5 in male: weakly rugulose laterally. Punctation of T5 in male: moderately dense throughout. Sculpture of S2: coarsely punctate throughout. Prominent longitudinal median carina on S2: absent.

##### Wings:

macropterous, apex or forewing extending beyond posterior margin of T3. Color of forewing in female: hyaline throughout. Color of forewing in male: hyaline throughout. Color of hind wing: hyaline throughout. Density of setation in fore wing: reduced posterior to Sc+R in basal half. Density of setation in hind wing: reduced posterior to Sc+R. Length of R1: more than 1.5 times as long as r. M+Cu and RS+M in forewing: spectral.

**Figures 144–149.**
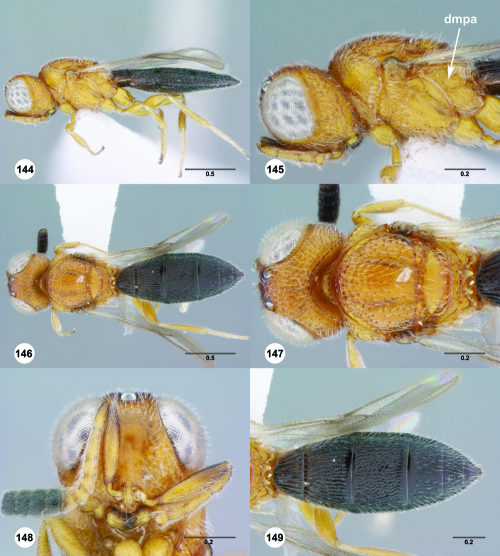
91143, Trichoteleia jiro sp. n., female holotype (CASENT 2043784). **144** Lateral habitus **145** Head and mesosoma, lateral view **146** Dorsal habitus **147** Head and mesosoma, dorsal view **148** Head, anterior view **149** Metasoma, dorsal view. Scale bars in millimeters.

#### Diagnosis.

Trichoteleia jiro is similar in habitus to a number of species that have a subspherical head, coarse punctation on the gena and vertex, and spectral veins in the forewing: Trichoteleia afo, Trichoteleia bicolor, Trichoteleia sphaerica and Trichoteleia minima. It may be may separated from them by the combination of a hyaline forewing, absence of horn on T1 in females, and dorsal metapleural area that is entirely to nearly entirely punctate rugose. Females of Trichoteleia jiro may also be recognized by the color pattern of bright orange head and mesosoma and black metasoma. However, females of Trichoteleia afo usually have a similar pattern but with a red head and mesosoma and black metasoma. Thus color should be used only as a supporting character for identification. Males of Trichoteleia jiro have a brown head and mesosoma and dark brown to black metasoma (Fig. 306), serving to distinguish it from the similarly shaped Trichoteleia afo.

#### Etymology.

The species name *jiro*, to be treated as a noun in apposition, means “torch” in Malagasy. This epithet refers to the color pattern of females in this species.

#### Link to Distribution Map.

[http://hol.osu.edu/map-large.html?id=241279]

#### Material Examined.

Holotype, female: **MADAGASCAR:** Toamasina Auto. Prov., botanical garden nr. entrance, tropical forest, MA-01-08B-20, Mantadia (Andasibe) National Park, 18°55.58'S 48°24.47'E, 1025m, 16.XI–23.XI.2001, malaise trap, R. Harin’Hala, CASENT 2043784 (deposited in CASC). Paratypes: **MADAGASCAR:** 4 females, 8 males, CASENT 2043516, 2043774, 2043785, 2133946–2133947, 2134005, 2136417, 2136587, 2136605 (CASC); OSUC 143329 (CNCI); CASENT 2043393, 2136590 (OSUC).

### Trichoteleia ketrona

Talamas sp. n.

urn:lsid:zoobank.org:act:D836A36A-71A5-4E77-B80B-D4FE8516BE66

urn:lsid.biosci.ohio-state.edu:osuc_concepts:241290

**Figures 150–155.**
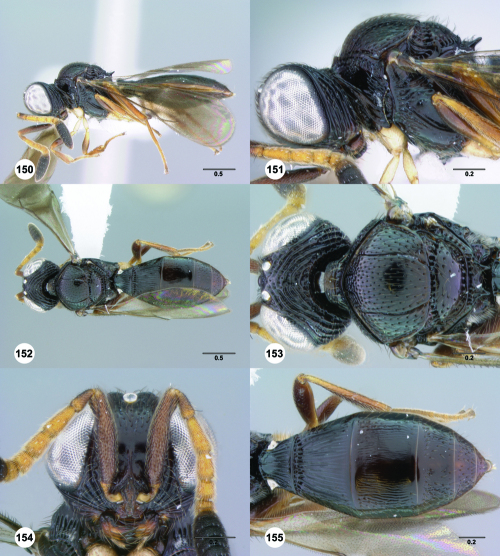
^92^Trichoteleia ketrona sp. n., female holotype (CASENT 2042842). **150** Lateral habitus **151** Head and mesosoma, lateral view **152** Dorsal habitus **153** Head and mesosoma, dorsal view **154** Head, anterior view **155** Metasoma, dorsal view. Scale bars in millimeters.

Figures 150–155 Morphbank ^23^ 

#### Description.

##### Female body length:

3.31 mm (n=1). Color of head: dark brown to black. Central keel of frons: present, extending onto interantennal process. Sculpture of medial frons in female: smooth. Number of mandibular teeth: three. Basal node on mandible: present. Sculpture of frons below median ocellus: finely punctate throughout, dorsoventrally strigose laterally. Sculpture of posterior vertex: concentrically rugose. Occipital rim: comprised of medium to large sized cells. Sculpture of gena: coarsely striate. Basiconic sensillum on A7: absent.

##### Color of mesosoma in female:

dark brown to black. Sculpture along posterior pronotal sulcus: striate, striae well defined. Notaulus: percurrent, reaching suprahumeral sulcus as a smooth furrow. Sculpture of medial mesoscutum: moderately punctate in posterior half, becoming denser anteriorly. Sculpture of mesoscutellum: smooth medially, sparsely punctate laterally. Postacetabular sulcus: comprised of small cells. Mesopleural carina: present. Sculpture along ventral half of prespecular sulcus: punctate rugose. Sculpture of posterolateral mesepisternum: smooth. Sculpture of ventral surface of mesepisternum: smooth. Setation of ventral metapleural area: absent. Setation of metapleural triangle: sparse. Sculpture of metapleural triangle: rugulose. Posterior margin of metapleuron below propodeal spiracle: straight to moderately convex. Color of legs: coxae and trochanters yellow, otherwise brown, becoming paler distally.

##### Color of metasoma in female:

dark brown to black throughout. Posterior margin of transverse sulcus on T2: strongly convex. Sublateral tergal carina on T2: absent. Microsculpture on T2: absent. Microsculpture on T3: absent. Microsculpture on T4: absent. Horn on T1 in female: present as a large, apically rounded protuberance. Macrosculpture of T2 in female: longitudinally striate throughout. Macrosculpture of medial T3 in female: weakly longitudinally striate. Macrosculpture of lateral T3 in female: longitudinally striate. Macrosculpture of medial T4 in female: absent. Macrosculpture of lateral T4 in female: punctate rugulose. Punctation of T4 in female: absent along midline, otherwise sparse. Macrosculpture of T5 in female: punctate rugulose laterally. Punctation of T5 in female: sparse in medial third, moderately dense laterally. Shape of T5 in female: width of posterior margin greater than or equal to length. Microscupture on T6 in female: absent. Sculpture of T6 in female: smooth with sparse moderate sized punctures. Sculpture of S2: longitudinally striate anteromedially, otherwise smooth. Prominent longitudinal median carina on S2: absent.

##### Wings:

macropterous, apex or forewing extending beyond posterior margin of T3. Color of forewing in female: slightly infuscate throughout. Color of hind wing: slightly infuscate throughout. Density of setation in fore wing: uniform throughout. Density of setation in hind wing: uniform throughout. Length of R1: more than 1.5 times as long as r. M+Cu and RS+M in forewing: nebulous.

#### Diagnosis.

This species has large concentric rugae along the posterior vertex similar to Trichoteleia irwini and Trichoteleia albidipes. It differs from them in that the rugae are less uniform and the head is transverse in dorsal view. Trichoteleia ketrona also does not have microsculpture on the metasoma (Fig. 155), and the anterior mesoscutum is strongly arched in lateral view (Fig. 151).

#### Etymology.

The word *ketrona*, meaning “wrinkled” in Malagasy, refers to the rugae on the posterior vertex in this species. The epithet is to be treated as a noun in apposition.

#### Link to Distribution Map.

[http://hol.osu.edu/map-large.html?id=241290]

#### Material Examined.

Holotype, female: **MADAGASCAR:** Fianarantsoa Auto. Prov., Talatakely, Bellevue, secondary tropical forest, MA-02-09C-60, Ranomafana National Park, 21°15.99'S 47°25.21'E, 1020m, 4.V–16.V.2003, malaise trap, R. Harin’Hala, CASENT 2042842 (deposited in CASC).

### Trichoteleia levii

Talamas & Johnson sp. n.

urn:lsid:zoobank.org:act:10011783-3277-4732-8899-4B99316BB1D9

urn:lsid.biosci.ohio-state.edu:osuc_concepts:243405

Figures 12 30 156–161 305 Morphbank ^24^ 

#### Description.

##### Female body length:

3.22–3.55 mm (n=6). Male body length: 3.01–3.45 mm (n=4). Color of head: dark orange, becoming brown at vertex; dark red, becoming darker dorsally; yellow throughout. Central keel of frons: present, extending onto interantennal process. Sculpture of medial frons in female: smooth. Sculpture of medial frons in male: smooth. Number of mandibular teeth: three. Basal node on mandible: absent. Sculpture of frons below median ocellus: moderately punctate throughout; finely punctate throughout. Sculpture of posterior vertex: moderately punctate; finely punctate. Occipital rim: comprised of medium to large sized cells; comprised of small to miniscule cells. Sculpture of gena: dorsoventrally strigose. Basiconic sensillum on A7: absent.

##### Color of mesosoma in female:

variably red to black; yellow throughout. Color of mesosoma in male: variably red to black; yellow. Sculpture along posterior pronotal sulcus: striate, striae well defined. Notaulus: percurrent, reaching suprahumeral sulcus as a smooth furrow. Sculpture of medial mesoscutum: moderately punctate in posterior half, becoming denser and finer anteriorly. Sculpture of mesoscutellum: smooth with sparse fine punctures throughout. Postacetabular sulcus: comprised of small cells. Mesopleural carina: present. Sculpture along ventral half of prespecular sulcus: longitudinally striate; finely punctate. Sculpture of posterolateral mesepisternum: smooth; smooth except for 1 or 2 striae parallel to mesopleural carina. Sculpture of ventral surface of mesepisternum: smooth. Setation of ventral metapleural area: absent. Setation of metapleural triangle: absent; sparse. Sculpture of metapleural triangle: smooth. Posterior margin of metapleuron below propodeal spiracle: straight to moderately convex; with blunt kink near intersection with metapleural sulcus. Color of legs: yellow throughout.

##### Color of metasoma in female:

dark brown to black throughout; yellow. Color of metasoma in male: dark brown to black throughout; variably yellow to brown throughout. Posterior margin of transverse sulcus on T2: strongly convex. Sublateral tergal carina on T2: absent. Microsculpture on T2: present. Microsculpture on T3: present. Microsculpture on T4: absent. Horn on T1 in female: present as a large, apically rounded protuberance. Macrosculpture of T2 in female: longitudinally striate throughout. Macrosculpture of medial T3 in female: weakly longitudinally striate; weakly longitudinally strigose; longitudinally striate. Macrosculpture of lateral T3 in female: longitudinally striate; longitudinally strigose. Macrosculpture of medial T4 in female: absent. Macrosculpture of lateral T4 in female: weakly longitudinally strigose; longitudinally striate. Punctation of T4 in female: absent in medial third, sparse laterally; sparse in medial third, dense laterally; absent along midline, otherwise sparse. Macrosculpture of T5 in female: absent; longitudinally strigose laterally. Punctation of T5 in female: moderately dense throughout; sparse in medial third, moderately dense laterally. Shape of T5 in female: width of posterior margin greater than or equal to length. Microscupture on T6 in female: present along anterior margin. Sculpture of T6 in female: finely punctate throughout. Macrosculpture of T2 in male: longitudinally striate throughout. Macrosculpture of medial T3 in male: weakly longitudinally striate; weakly longitudinally strigose. Macrosculpture of lateral T3 in male: longitudinally striate; longitudinally strigose. Macrosculpture of T4 in male: longitudinally strigose laterally; punctate rugulose laterally. Punctation of T4 in male: sparse throughout; sparse in medial third, dense laterally; absent along midline, otherwise sparse. Macrosculpture of T5 in male: absent. Punctation of T5 in male: dense throughout; moderately dense throughout. Sculpture of S2: longitudinally striate anteriorly, smooth posteriorly. Prominent longitudinal median carina on S2: absent.

##### Wings:

macropterous, apex or forewing extending beyond posterior margin of T3. Color of forewing in female: slightly infuscate throughout; slightly infuscate with longitudinal dark streak. Color of forewing in male: slightly infuscate throughout. Color of hind wing: slightly infuscate throughout. Density of setation in fore wing: uniform throughout. Density of setation in hind wing: uniform throughout. Length of R1: more than 1.5 times as long as r. M+Cu and RS+M in forewing: nebulous.

**Figures 156–161.**
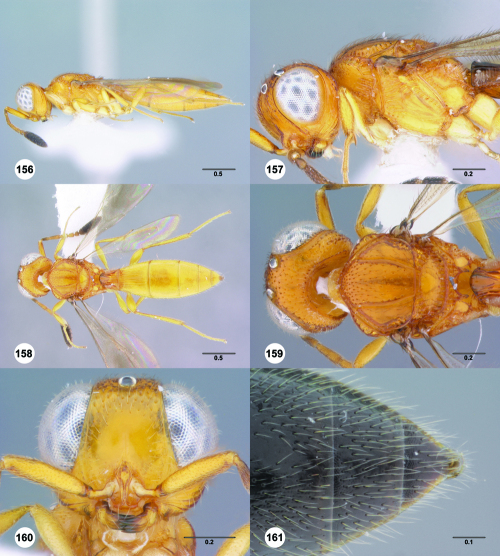
^93^Trichoteleia levii sp. n. **156** Lateral habitus, female (CASENT 2135725) **157** Head and mesosoma, lateral view, female (CASENT 2135726) **158** Dorsal habitus, female (CASENT 2135727) **159** Head and mesosoma, dorsal view, female (CASENT 2135727) **160** Head, anterior view, female (CASENT 2135726) **161** T5**–**T6, dorsal view, female (CASENT 2043789). Scale bars in millimeters.

#### Diagnosis.

Trichoteleia levii belongs to the cluster of species, including Trichoteleia prima, and Trichoteleia tonsa, in which the short striae along the pronotal shoulder and those of the lateral pronotum form a continuous band. Trichoteleia levii can be separated from Trichoteleia tonsa and Trichoteleia prima by the antero-posteriorly compressed axillular carina (Fig. 12) and by the combination of a smooth metapleural triangle and the absence of microsculpture on the posterior half of T6.

#### Etymology.

Trichoteleia levii is named for T. Levi Blankenship (USA), a friend of the first author, in commemoration of his marriage to Mirjana Milosavljevic-Cook (USA).

#### Link to Distribution Map.

[http://hol.osu.edu/map-large.html?id=243405]

#### Material Examined.

Holotype, female: **MADAGASCAR:** Antsiranana Auto. Prov., 9.1km (233°) SW Daraina, rainforest, BLF9657, Binara Forest, 13°15'48’’S 49°36'12’’E, 650–800m, 3.XII.2003, yellow pan trap, B. L. Fisher, CASENT 2135724 (deposited in CASC). Paratypes: **MADAGASCAR:** 5 females, 4 males, CASENT 2043788, 2043790, 2135725–2135726, 2135729–2135730 (CASC); CASENT 2043789, 2135727–2135728 (OSUC).

#### Comments:

Two color patterns, corresponding with different collecting locations, exist in this species. The specimens found near the northern tip of Antsiranana were collected in dwarf forest along Kakalava beach and have a red head and mesosoma and a black metasoma (161, 305). Specimens collected farther south in rainforest habitat are slightly smaller and almost entirely yellow with finer punctation on the head and mesosoma (Figs 156–160).

### Trichoteleia longiventris

Talamas & Masner sp. n.

urn:lsid:zoobank.org:act:4050C511-3E5C-4A80-8A28-CC895E3BCD3C

urn:lsid.biosci.ohio-state.edu:osuc_concepts: 238235

**Figures 162–167.**
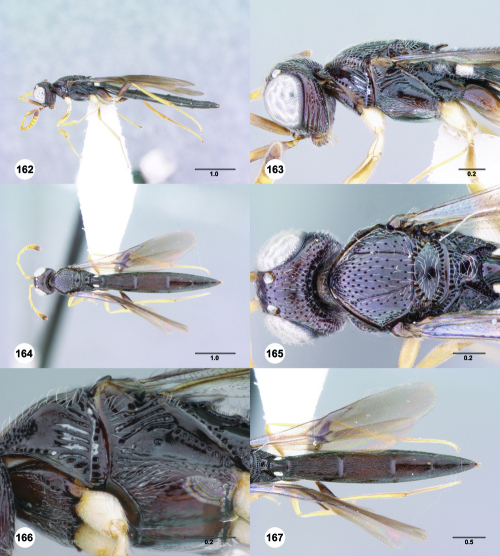
^94^Trichoteleia longiventris sp. n., female holotype (OSUC 181014). **162** Lateral habitus **163** Head and mesosoma, lateral view **164** Dorsal habitus **165** Head and mesosoma, dorsal view **166** Head, anterior view **167** Metasoma, dorsal view. Scale bars in millimeters.

Figures 162–167 Morphbank ^25^ 

#### Description.

##### Female body length:

4.80 mm (n=1). Color of head: reddish brown. Central keel of frons: present, extending onto interantennal process. Sculpture of medial frons in female: smooth. Number of mandibular teeth: three. Basal node on mandible: absent. Sculpture of frons below median ocellus: finely punctate throughout. Sculpture of posterior vertex: finely punctate. Occipital rim: comprised of medium to large sized cells. Sculpture of gena: dorsoventrally strigose. Basiconic sensillum on A7: absent.

##### Color of mesosoma in female:

reddish brown throughout. Sculpture along posterior pronotal sulcus: striate, striae well defined. Notaulus: percurrent, reaching suprahumeral sulcus as a smooth furrow. Sculpture of medial mesoscutum: moderately punctate in posterior half, becoming denser anteriorly. Sculpture of mesoscutellum: smooth with sparse fine punctures throughout. Postacetabular sulcus: present as a smooth furrow. Mesopleural carina: present only in anterior half. Sculpture along ventral half of prespecular sulcus: punctate rugose. Sculpture of posterolateral mesepisternum: smooth except for 1 or 2 rows of fine punctures parallel to mesopleural carina. Sculpture of ventral surface of mesepisternum: smooth. Setation of ventral metapleural area: absent. Setation of metapleural triangle: sparse. Sculpture of metapleural triangle: densely punctate. Posterior margin of metapleuron below propodeal spiracle: with blunt kink near intersection with metapleural sulcus. Color of legs: fore and mid coxa white, hind coxa and femur brown, trochanters white, legs otherwise yellow.

##### Color of metasoma in female:

dark brown to black throughout. Posterior margin of transverse sulcus on T2: strongly convex. Sublateral tergal carina on T2: absent. Microsculpture on T2: present. Microsculpture on T3: present. Microsculpture on T4: present. Horn on T1 in female: present as a large, apically rounded protuberance. Macrosculpture of T2 in female: longitudinally strigose throughout. Macrosculpture of medial T3 in female: rugulose. Macrosculpture of lateral T3 in female: longitudinally strigose. Macrosculpture of medial T4 in female: absent. Macrosculpture of lateral T4 in female: absent. Punctation of T4 in female: sparse along midline, otherwise dense. Macrosculpture of T5 in female: absent. Punctation of T5 in female: dense throughout. Shape of T5 in female: width of posterior margin less than length. Sculpture of T6 in female: densely and finely punctate. Sculpture of S2: densely punctate, punctures of moderate size. Prominent longitudinal median carina on S2: absent.

##### Wings:

macropterous, apex or forewing extending beyond posterior margin of T3. Color of forewing in female: slightly infuscate throughout with metallic purple sheen. Color of hind wing: slightly infuscate throughout. Density of setation in fore wing: uniform throughout. Density of setation in hind wing: uniform throughout. Length of R1: more than 1.5 times as long as r. M+Cu and RS+M in forewing: nebulous.

#### Diagnosis.

Trichoteleia longiventris, Trichoteleia quazii and Trichoteleia carinata are elongate species that have dark to moderate infuscation throughout the forewing and darkly pigmented Rs+M and M+Cu veins. Trichoteleia quazii and Trichoteleia carinata differ from Trichoteleia longiventris by having a longitudinal median carina on S2 (Fig. 45).

#### Etymology.

Trichoteleia longiventris is named for its extremely long metasoma. The epithet is a noun in apposition.

#### Link to Distribution Map.

[http://hol.osu.edu/map-large.html?id=238235]

#### Material Examined.

Holotype, female: **MADAGASCAR:** Antsiranana Auto. Prov., 12.2km WSW Befingotra, #1271, Anjanaharibe-Sud Special Reserve, 14°45'S 49°26'E, 1960m, 24.XI–29.XI.1994, B. L. Fisher, OSUC 181014 (deposited in UCDC).

### Trichoteleia minima

Talamas sp. n.

urn:lsid:zoobank.org:act:D098AF79-F72D-4D9F-A46E-AE8BC20E5F15

urn:lsid.biosci.ohio-state.edu:osuc_concepts: 243407

Figures 10 21 168–173 Morphbank ^26^ 

#### Description.

##### Female body length:

2.87–2.91 mm (n=2). Color of head: reddish brown. Central keel of frons: present, ending above interantennal process. Sculpture of medial frons in female: smooth. Number of mandibular teeth: three. Basal node on mandible: absent. Sculpture of frons below median ocellus: finely punctate throughout. Sculpture of posterior vertex: moderately punctate. Occipital rim: comprised of medium to large sized cells. Sculpture of gena: punctate rugose. Basiconic sensillum on A7: absent.

##### Color of mesosoma in female:

reddish brown throughout. Sculpture along posterior pronotal sulcus: striate, striae short and poorly defined. Notaulus: smooth furrow incomplete, reaching suprahumeral sulcus as row of punctures. Sculpture of medial mesoscutum: smooth and sparsely punctate posteriorly, densely punctate and transverely rugulose anteriorly. Sculpture of mesoscutellum: smooth with few very fine punctures laterally. Postacetabular sulcus: comprised of large cells. Mesopleural carina: present. Sculpture along ventral half of prespecular sulcus: longitudinally striate; coarsely punctate. Sculpture of posterolateral mesepisternum: coarsely punctate in longitudinal rows. Sculpture of ventral surface of mesepisternum: finely punctate. Setation of ventral metapleural area: absent. Setation of metapleural triangle: dense. Sculpture of metapleural triangle: punctate rugose. Posterior margin of metapleuron below propodeal spiracle: with triangular point near intersection with metapleural sulcus; with blunt kink near intersection with metapleural sulcus. Color of legs: brown throughout.

##### Color of metasoma in female:

S1–S2 orange, otherwise reddish brown. Posterior margin of transverse sulcus on T2: weakly convex. Sublateral tergal carina on T2: absent. Microsculpture on T2: present. Microsculpture on T3: present. Microsculpture on T4: present. Horn on T1 in female: absent. Macrosculpture of T2 in female: longitudinally strigose throughout. Macrosculpture of medial T3 in female: longitudinally strigose; rugulose. Macrosculpture of lateral T3 in female: longitudinally strigose. Macrosculpture of medial T4 in female: absent. Macrosculpture of lateral T4 in female: longitudinally strigose. Punctation of T4 in female: absent along midline, otherwise sparse. Macrosculpture of T5 in female: absent. Punctation of T5 in female: absent along midline, otherwise moderately dense. Shape of T5 in female: width of posterior margin greater than or equal to length. Microscupture on T6 in female: absent. Sculpture of T6 in female: finely punctate throughout. Sculpture of S2: coarsely punctate throughout, with longitudinal rugae laterally. Prominent longitudinal median carina on S2: absent.

##### Wings:

macropterous, apex or forewing extending beyond posterior margin of T3. Color of forewing in female: hyaline with transverse infuscate bands medially and apically. Color of hind wing: hyaline throughout. Density of setation in fore wing: reduced posterior to Sc+R in basal half. Density of setation in hind wing: reduced posterior to Sc+R. Length of R1: more than 1.5 times as long as r. M+Cu and RS+M in forewing: spectral.

**Figures 168–173.**
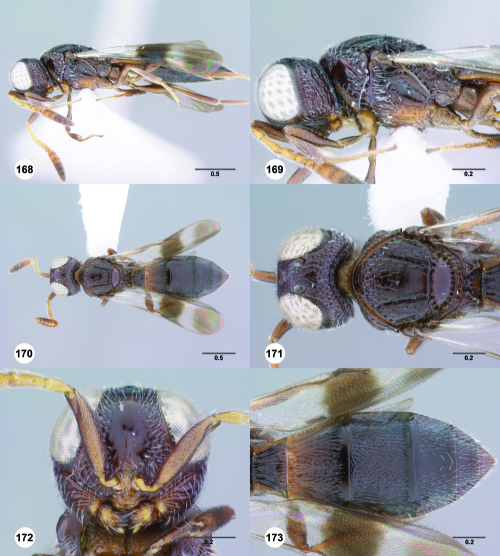
^95^Trichoteleia minima sp. n. **168** Lateral habitus, female (CASENT 2132759) **169** Head and mesosoma, lateral view, female (CASENT 2135729) **170** Dorsal habitus, female holotype (CASENT 2043543) **171** Head and mesosoma, dorsal view, female holotype (CASENT 2043543) **172** Head, anterior view, female holotype (CASENT 2043543) **173** Metasoma, dorsal view, female holotype (CASENT 2043543). Scale bars in millimeters.

#### Diagnosis.

Trichoteleia minima is similar to Trichoteleia afo and Trichoteleia jiro with which it shares the absence of a prominent orbital carina and the lack of a horn on T1 in females. It is easily separated from them by the very small metascutellar points, darkly banded forewing, and longitudinally strigose sculpture on T3.

#### Etymology.

The adjectival epithet *minima*, meaning “smallest”, refers to the size of the metascutellar points in this species.

#### Link to Distribution Map.

[http://hol.osu.edu/map-large.html?id=243407]

#### Material Examined.

Holotype, female: **MADAGASCAR:** Fianarantsoa Auto. Prov., Vohiparara, broken bridge, high altitude rainforest, MA-02-09A-12, Ranomafana National Park, 21°13.57'S 47°22.19'E, 1100m, 14.I–21.I.2002, malaise trap, R. Harin’Hala, CASENT 2043543 (deposited in CASC). Paratype: **MADAGASCAR:** 1 female, CASENT 2132759 (CASC).

### Trichoteleia nify

Talamas & Masner sp. n.

urn:lsid:zoobank.org:act:5C03417F-D16B-4882-BFB9-98BA380A6050

urn:lsid.biosci.ohio-state.edu:osuc_concepts:241272

Figures 29 34 37 174–179 Morphbank ^27^ 

#### Description.

##### Female body length:

2.14–2.70 mm (n=9). Male body length: 2.22–2.90 mm (n=20). Color of head: dark brown to black; yellow, becoming darker dorsally; yellow throughout. Central keel of frons: present, extending onto interantennal process. Sculpture of medial frons in female: smooth. Sculpture of medial frons in male: smooth. Number of mandibular teeth: three. Basal node on mandible: present. Sculpture of frons below median ocellus: punctate rugulose throughout; finely punctate throughout, dorsoventrally strigose laterally; moderately punctate throughout. Sculpture of posterior vertex: moderately punctate; moderately punctate, rugose posterior to eyes and posterior ocellus; finely punctate. Occipital rim: comprised of medium to large sized cells. Sculpture of gena: punctate rugose; dorsoventrally strigose. Basiconic sensillum on A7: absent.

##### Color of mesosoma in female:

variably red to black; variably yellow to brown. Color of mesosoma in male: dark brown to black; variably yellow to brown. Sculpture along posterior pronotal sulcus: striate, striae well defined. Notaulus: percurrent, reaching suprahumeral sulcus as a smooth furrow. Sculpture of medial mesoscutum: moderately punctate in posterior half, becoming denser anteriorly. Sculpture of mesoscutellum: smooth medially, sparsely punctate laterally. Postacetabular sulcus: comprised of small cells. Mesopleural carina: present. Sculpture along ventral half of prespecular sulcus: punctate rugose. Sculpture of posterolateral mesepisternum: smooth. Sculpture of ventral surface of mesepisternum: smooth. Setation of ventral metapleural area: absent. Setation of metapleural triangle: sparse. Sculpture of metapleural triangle: punctate rugose. Posterior margin of metapleuron below propodeal spiracle: with posterolaterally projecting spine near intersection with metapleural sulcus. Color of legs: yellow throughout; brown throughout.

##### Color of metasoma in female:

yellow to pale brown, posterolateral corners of T2–T3 dark brown. Color of metasoma in male: variably yellow to brown throughout. Posterior margin of transverse sulcus on T2: strongly convex. Sublateral tergal carina on T2: absent. Microsculpture on T2: absent. Microsculpture on T3: absent. Microsculpture on T4: absent. Horn on T1 in female: present as a large, apically rounded protuberance; present as a small bulge. Macrosculpture of T2 in female: longitudinally striate throughout. Macrosculpture of medial T3 in female: weakly longitudinally striate; longitudinally striate. Macrosculpture of lateral T3 in female: longitudinally striate. Macrosculpture of medial T4 in female: absent. Macrosculpture of lateral T4 in female: obliquely strigose; weakly obliquely rugulose. Punctation of T4 in female: sparse in medial third, moderately dense laterally. Macrosculpture of T5 in female: absent. Punctation of T5 in female: moderately dense throughout. Shape of T5 in female: width of posterior margin greater than or equal to length. Microscupture on T6 in female: absent. Sculpture of T6 in female: smooth with fine setigerous punctures along lateral margin. Macrosculpture of T2 in male: striate anteriorly, with few striae reaching T3. Macrosculpture of medial T3 in male: absent. Macrosculpture of lateral T3 in male: absent; weakly longitudinally striate. Macrosculpture of T4 in male: absent. Punctation of T4 in male: sparse in medial third, moderately dense laterally; sparse throughout; absent in medial third, sparse laterally. Macrosculpture of T5 in male: absent. Punctation of T5 in male: moderately dense throughout. Sculpture of S2: longitudinally striate anteriorly, smooth posteriorly. Prominent longitudinal median carina on S2: absent.

##### Wings:

macropterous, apex or forewing extending beyond posterior margin of T3. Color of forewing in female: slightly infuscate throughout. Color of forewing in male: slightly infuscate throughout. Color of hind wing: slightly infuscate throughout. Density of setation in fore wing: uniform throughout. Density of setation in hind wing: reduced posterior to Sc+R. Length of R1: more than 1.5 times as long as r. M+Cu and RS+M in forewing: nebulous.

**Figures 174–179.**
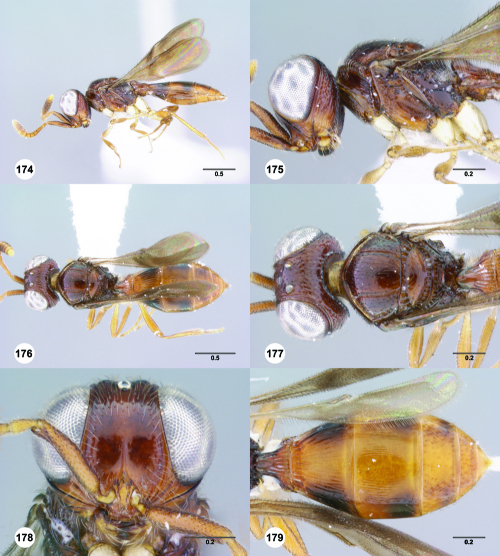
^96^Trichoteleia nify sp. n. **174** Lateral habitus, female holotype (CASENT 2135909) **175** Head and mesosoma, lateral view, female holotype (CASENT 2135909) **176** Dorsal habitus, female holotype (CASENT 2135909) **177** Head and mesosoma, dorsal view, female holotype (CASENT 2135909) **178** Head, anterior view, female (CASENT 2135910) **179** Metasoma, dorsal view, female (CASENT 2135910). Scale bars in millimeters.

#### Diagnosis.

Trichoteleia nify may be identified by the presence of a spine along the posterior margin of the metapleuron near the intersection of the margin with the metapleural sulcus (Fig. 29) and the presence of a flange dorsal to the lateral setal patch on T1 (Fig. 29).

#### Etymology.

This species is given the name *nify*, a noun in apposition, meaning “tooth” in Malagasy, in reference to its metapleural spine.

#### Link to Distribution Map.

[http://hol.osu.edu/map-large.html?id=241272]

#### Material Examined.

Holotype, female: **MADAGASCAR:** Fianarantsoa Auto. Prov., 7.5km ENE Ivohibe, #1745, Pic d’Ivohibe Special Reserve, 22°28.2'S 46°57.6'E, 900m, 7.X–12.X.1997, B. L. Fisher, CASENT 2135909 (deposited in CASC). Paratypes: **MADAGASCAR:** 12 females, 80 males, CASENT 2118402, 2118405, 2134160, 2134166, 2134170, 2135782–2135786, 2135790, 2135792–2135793, 2135797, 2135799–2135800, 2135804–2135805, 2135813, 2135824, 2135831, 2135834, 2135837–2135840, 2135847–2135849, 2135873, 2135882–2135887, 2135901, 2136000, 2136002, 2136004, 2136006–2136008, 2136011, 2136026, 2136122, 2136124–2136125, 2136136, 2136145, 2136150, 2136152, 2136155, 2136160, 2136163, 8106458, 8106462, OSUC 181028, OSUC 266089, OSUC 266094 (CASC); CASENT 2134088, 2134101 (CNCI); CASENT 2135806, 2135823, 2135872, 2135910, 2135968, 2136010, 2136014, 2136016, 2136018, 2136021, 2136069, 2136126, 2136137–2136138, 2136140–2136141, 2136144, 2136146, 2136153–2136154, 2136159, 2136167 (OSUC); OSUC 181029–181036 (USNM).

### Trichoteleia oculea

Talamas & Masner sp. n.

urn:lsid:zoobank.org:act:4F9FCD1E-B5ED-4AFA-8AB7-7959C60A1A6F

urn:lsid.biosci.ohio-state.edu:osuc_concepts: 243415

Figures 15 26 180–185 Morphbank ^28^ 

#### Description.

##### Female body length:

2.94–3.36 mm (n=3). Color of head: reddish brown. Central keel of frons: present, extending onto interantennal process. Sculpture of medial frons in female: smooth. Number of mandibular teeth: three. Basal node on mandible: present. Sculpture of frons below median ocellus: moderately punctate throughout; punctate laterally, mostly smooth medially. Sculpture of posterior vertex: moderately punctate medially, densely punctate laterally. Occipital rim: comprised of medium to large sized cells. Sculpture of gena: dorsoventrally strigose. Basiconic sensillum on A7: absent.

##### Color of mesosoma in female:

reddish brown throughout. Sculpture along posterior pronotal sulcus: striate, striae well defined. Notaulus: percurrent, reaching suprahumeral sulcus as a smooth furrow. Sculpture of medial mesoscutum: sparsely punctate, becoming denser anteriorly. Sculpture of mesoscutellum: smooth with sparse fine punctures throughout. Postacetabular sulcus: comprised of large cells. Mesopleural carina: present. Sculpture along ventral half of prespecular sulcus: finely punctate. Sculpture of posterolateral mesepisternum: smooth. Sculpture of ventral surface of mesepisternum: smooth. Setation of ventral metapleural area: absent. Setation of metapleural triangle: sparse. Sculpture of metapleural triangle: rugulose; punctate rugose. Posterior margin of metapleuron below propodeal spiracle: with blunt kink near intersection with metapleural sulcus. Color of legs: pale brown throughout.

##### Color of metasoma in female:

reddish brown. Posterior margin of transverse sulcus on T2: strongly convex. Sublateral tergal carina on T2: absent. Microsculpture on T2: present. Microsculpture on T3: present. Microsculpture on T4: present. Horn on T1 in female: absent. Macrosculpture of T2 in female: longitudinally striate throughout. Macrosculpture of medial T3 in female: absent; weakly longitudinally strigose. Macrosculpture of lateral T3 in female: longitudinally strigose. Macrosculpture of medial T4 in female: absent. Macrosculpture of lateral T4 in female: punctate rugulose; longitudinally strigose. Punctation of T4 in female: absent along midline, otherwise moderately dense. Macrosculpture of T5 in female: absent. Punctation of T5 in female: moderately dense throughout. Shape of T5 in female: width of posterior margin greater than or equal to length. Microscupture on T6 in female: absent. Sculpture of T6 in female: smooth with sparse fine setigerous punctures throughout. Sculpture of S2: longitudinally strigose throughout, punctate interstitially. Prominent longitudinal median carina on S2: absent.

##### Wings:

macropterous, apex or forewing extending beyond posterior margin of T3. Color of forewing in female: infuscate throughout. Color of hind wing: infuscate throughout. Density of setation in fore wing: uniform throughout. Density of setation in hind wing: uniform throughout. Length of R1: less than 1.5 times as long as rs. M+Cu and RS+M in forewing: nebulous.

**Figures 180–185.**
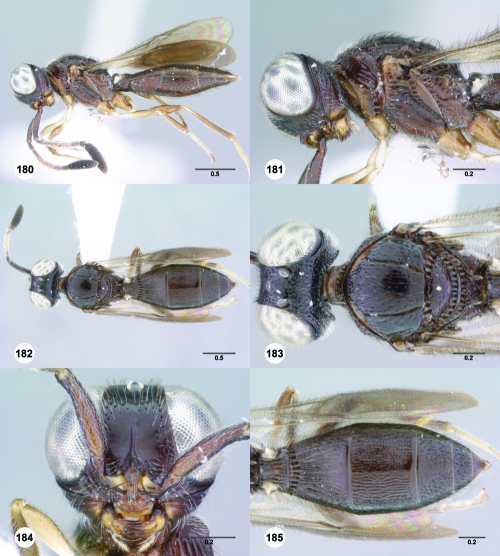
^97^Trichoteleia oculea sp. n. **180** Lateral habitus, female (CASENT 2135908) **181** Head and mesosoma, lateral view, female (CASENT 2135908) **182** Dorsal habitus, female holotype (CASENT 2135906) **183** Head and mesosoma, dorsal view, female holotype (CASENT 2135906) **184** Head, anterior view, female holotype (CASENT 2135906) **185** Metasoma, dorsal view, female holotype (CASENT 2135906). Scale bars in millimeters.

#### Diagnosis.

Trichoteleia oculea is most similar to Trichoteleia tahotra with which it shares an apical projection on the lateral propodeal area and R1 (postmarginal vein) approximately equal in length to r (stigmal vein) in the forewing. These species are easily separated by the prominent striation of the lateral pronotum (Fig. 15) and lack of a basiconic sensillum on A7 in Trichoteleia oculea (as in Fig. 2).

#### Etymology.

The adjectival epithet *oculea*, meaning “having many eyes,” refers to the large size of the eyes in this species.

#### Link to Distribution Map.

[http://hol.osu.edu/map-large.html?id=243415]

#### Material Examined.

Holotype, female: **MADAGASCAR:** Antsiranana Auto. Prov., 10.8km (229°) SW Antanambao, leaf mold / rotten wood / rainforest, #1996, Manongarivo Special Reserve, 13°57.7'S 48°26.0'E, 400m, 8.XI.1998, sifted litter, B. L. Fisher, CASENT 2135906 (deposited in CASC). Paratypes: **MADAGASCAR:** 2 females, CASENT 2135907–2135908 (CASC).

### Trichoteleia orona

Talamas & Masner sp. n.

urn:lsid:zoobank.org:act:1B4F6F9B-BEE9-430E-B95E-970F77175F95

urn:lsid.biosci.ohio-state.edu:osuc_concepts:241278

Figures 2 40 48 186–191 Morphbank ^29^ 

#### Description.

##### Female body length:

4.15–4.50 mm (n=6). Male body length: 3.24–4.07 mm (n=6). Color of head: dark brown to black. Central keel of frons: present, extending onto interantennal process. Sculpture of medial frons in female: smooth. Sculpture of medial frons in male: smooth. Number of mandibular teeth: three. Basal node on mandible: present. Sculpture of frons below median ocellus: dorsoventrally strigose throughout; dorsoventrally strigose throughout with coarsely punctate interstices. Sculpture of posterior vertex: concentrically rugose; moderately punctate, rugose posterior to eyes and posterior ocellus. Occipital rim: comprised of medium to large sized cells. Sculpture of gena: dorsoventrally strigose. Basiconic sensillum on A7: absent.

##### Color of mesosoma in female:

dark brown to black. Color of mesosoma in male: dark brown to black. Sculpture along posterior pronotal sulcus: striate, striae well defined. Notaulus: percurrent, reaching suprahumeral sulcus as a smooth furrow. Sculpture of medial mesoscutum: moderately punctate in posterior half, becoming denser anteriorly. Sculpture of mesoscutellum: smooth medially, sparsely punctate laterally. Postacetabular sulcus: comprised of small cells. Mesopleural carina: present. Sculpture along ventral half of prespecular sulcus: areolate rugose; coarsely punctate. Sculpture of posterolateral mesepisternum: smooth; punctate; smooth except for 1 or 2 rows of fine punctures parallel to mesopleural carina. Sculpture of ventral surface of mesepisternum: finely punctate; smooth. Setation of ventral metapleural area: absent. Setation of metapleural triangle: sparse. Sculpture of metapleural triangle: rugulose; punctate rugose. Posterior margin of metapleuron below propodeal spiracle: with blunt kink near intersection with metapleural sulcus. Color of legs: coxae and trochanters yellow, otherwise brown, becoming paler distally.

##### Color of metasoma in female:

dark brown to black throughout. Color of metasoma in male: dark brown to black throughout. Posterior margin of transverse sulcus on T2: strongly convex. Sublateral tergal carina on T2: absent. Microsculpture on T2: absent. Microsculpture on T3: absent. Microsculpture on T4: absent. Horn on T1 in female: present as a large, apically rounded protuberance. Macrosculpture of T2 in female: longitudinally striate throughout. Macrosculpture of medial T3 in female: transversely striate. Macrosculpture of lateral T3 in female: longitudinally strigose; rugulose. Macrosculpture of medial T4 in female: absent. Macrosculpture of lateral T4 in female: weakly longitudinally strigose. Punctation of T4 in female: sparse in medial third, moderately dense laterally; absent along midline, otherwise moderately dense. Macrosculpture of T5 in female: weakly rugulose laterally. Punctation of T5 in female: moderately dense throughout; dense throughout; sparse along midline, otherwise dense. Shape of T5 in female: width of posterior margin greater than or equal to length. Microscupture on T6 in female: present along anterior margin. Sculpture of T6 in female: moderately punctate throughout. Macrosculpture of T2 in male: longitudinally striate throughout. Macrosculpture of medial T3 in male: longitudinally striate; transversely striate; weakly rugulose. Macrosculpture of lateral T3 in male: longitudinally strigose. Macrosculpture of T4 in male: absent; weakly rugulose laterally. Punctation of T4 in male: sparse along midline, otherwise dense. Macrosculpture of T5 in male: absent. Punctation of T5 in male: dense throughout. Sculpture of S2: longitudinally striate anteriorly, smooth posteriorly; longitudinally striate medially, punctate interstitially. Prominent longitudinal median carina on S2: absent.

##### Wings:

macropterous, apex or forewing extending beyond posterior margin of T3. Color of forewing in female: infuscate throughout. Color of forewing in male: infuscate throughout. Color of hind wing: infuscate throughout. Density of setation in fore wing: uniform throughout. Density of setation in hind wing: uniform throughout. Length of R1: more than 1.5 times as long as r. M+Cu and RS+M in forewing: nebulous.

**Figures 186–191.**
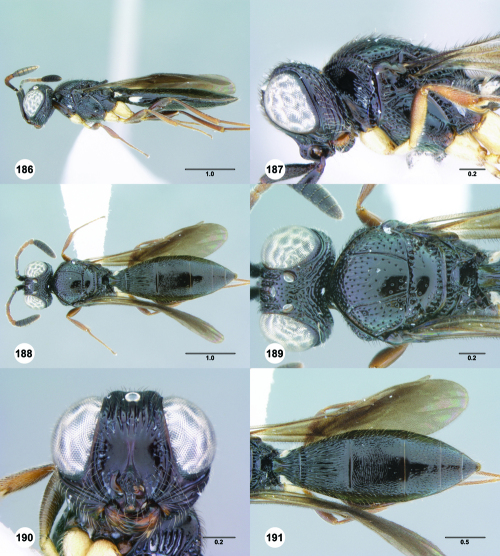
^98^Trichoteleia orona sp. n. **186** Lateral habitus, female holotype (CASENT 2043194) **187** Head and mesosoma, lateral view, female (CASENT 2043432) **188** Dorsal habitus, female holotype (CASENT 2043194) **189** Head and mesosoma, dorsal view, female holotype (CASENT 2043194) **190** Head, anterior view, female (CASENT 2136251) **191** Metasoma, dorsal view, female holotype (CASENT 2043194). Scale bars in millimeters.

#### Diagnosis.

Trichoteleia orona is a large and distinctive species. The females can be identified by the transverse striation of medial T3 alone (Fig. 191). Males are very similar to Trichoteleia takariva and can be reliably separated by the presence of a dorsal flange on the interantennal process (Fig. 187) and a densely punctate T5 (Fig. 40).

#### Etymology.

The name *orona*, meaning “nose” in Malagasy, is given to this species, as a noun in apposition, for the dorsal flange on the interantennal process.

#### Link to Distribution Map.

[http://hol.osu.edu/map-large.html?id=241278]

#### Material Examined.

Holotype, female: **MADAGASCAR:** Fianarantsoa Auto. Prov., Talatakely, Bellevue, secondary tropical forest, MA-02-09C-03, Ranomafana National Park, 21°15.99'S 47°25.21'E, 1020m, 15.XI–22.XI.2001, malaise trap, R. Harin’Hala, CASENT 2043194 (deposited in CASC). Paratypes: **MADAGASCAR:** 7 females, 6 males, CASENT 2008633, 2043795, 2134153, 2134171, 2135967, 2136251, OSUC 181003 (CASC); CASENT 2134181, OSUC 181005 (CNCI); CASENT 2043432, 2136612, OSUC 254611 (OSUC); OSUC 181004 (USNM).

### Trichoteleia parvipennis

Talamas & Masner sp. n.

urn:lsid:zoobank.org:act:A862CAA6-67ED-4F68-8ADA-9EB5F980728C

urn:lsid.biosci.ohio-state.edu:osuc_concepts:243850

**Figures 192–197.**
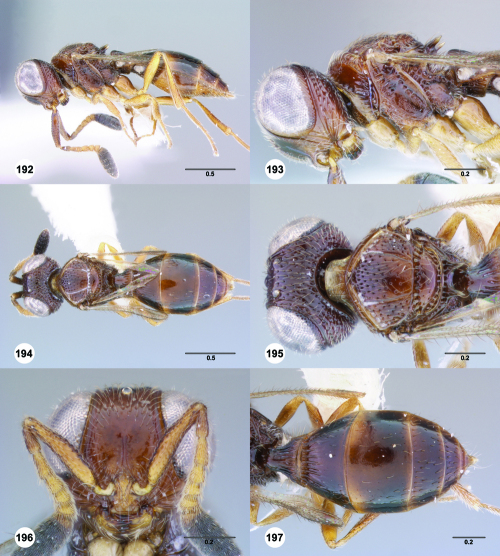
^99^Trichoteleia parvipennis sp. n. **192** Lateral habitus, female holotype (OSUC 181021) **193** Head and mesosoma, lateral view, female holotype (OSUC 181021) **194** Dorsal habitus, female (OSUC 181022) **195** Head and mesosoma, dorsal view, female holotype (OSUC 181021) **196** Head, anterior view, female (OSUC 181023) **197** Metasoma, dorsal view, female holotype (OSUC 181021). Scale bars in millimeters.

Figures 192–197 Morphbank ^30^ 

#### Description.

##### Female body length:

2.17–2.34 mm (n=4). Color of head: reddish brown. Central keel of frons: present, extending onto interantennal process. Sculpture of medial frons in female: smooth. Number of mandibular teeth: three. Basal node on mandible: present. Sculpture of frons below median ocellus: finely punctate throughout, dorsoventrally strigose laterally; moderately punctate throughout. Sculpture of posterior vertex: moderately punctate; moderately punctate, rugose posterior to eyes and posterior ocellus. Occipital rim: comprised of small to miniscule cells. Sculpture of gena: punctate rugulose; dorsoventrally strigose. Basiconic sensillum on A7: present.

##### Color of mesosoma in female:

variably yellow to brown. Sculpture along posterior pronotal sulcus: striate, striae well defined; striate, striae short and poorly defined. Notaulus: percurrent, reaching suprahumeral sulcus as a smooth furrow. Sculpture of medial mesoscutum: sparsely punctate, becoming denser anteriorly. Sculpture of mesoscutellum: smooth with sparse fine punctures throughout. Postacetabular sulcus: comprised of small cells. Mesopleural carina: present. Sculpture along ventral half of prespecular sulcus: areolate rugose. Sculpture of posterolateral mesepisternum: areolate rugose. Sculpture of ventral surface of mesepisternum: smooth. Setation of ventral metapleural area: absent. Setation of metapleural triangle: sparse. Sculpture of metapleural triangle: rugulose. Posterior margin of metapleuron below propodeal spiracle: with triangular point near intersection with metapleural sulcus. Color of legs: yellow throughout; pale brown throughout.

##### Color of metasoma in female:

variably patterned in alternating orange and brown. Posterior margin of transverse sulcus on T2: strongly convex. Sublateral tergal carina on T2: absent. Microsculpture on T2: absent. Microsculpture on T3: absent. Microsculpture on T4: absent. Horn on T1 in female: present as a large protuberance, curved posteriorly at apex. Macrosculpture of T2 in female: striate anteriorly, with few striae reaching T3. Macrosculpture of medial T3 in female: absent. Macrosculpture of lateral T3 in female: absent. Macrosculpture of medial T4 in female: absent. Macrosculpture of lateral T4 in female: absent; weakly obliquely rugulose along anterior margin. Punctation of T4 in female: absent along midline, otherwise sparse. Macrosculpture of T5 in female: absent. Punctation of T5 in female: moderately dense throughout. Shape of T5 in female: width of posterior margin greater than or equal to length. Microscupture on T6 in female: absent. Sculpture of T6 in female: smooth with fine setigerous punctures along lateral margin. Sculpture of S2: crenulate along anterior margin, otherwise smooth with small punctures throughout. Prominent longitudinal median carina on S2: absent.

##### Wings:

brachypterous, apex of forewing ending before midpoint of T3. Color of forewing in female: slightly infuscate throughout. Color of hind wing: slightly infuscate throughout. Density of setation in fore wing: uniform throughout. Density of setation in hind wing: uniform throughout. Length of R1: more than 1.5 times as long as r.

#### Diagnosis.

Trichoteleia parvipennis, Trichoteleia echinata and Trichoteleia halterata are the only known brachypterous species of Trichoteleia. Trichoteleia parvipennis has a basiconic sensillum on A7 that separates it from these species in the female sex.

#### Etymology.

The Latin name *parvipennis,* a noun in apposition, refers to the reduced size of the wings.

#### Link to Distribution Map.

[http://hol.osu.edu/map-large.html?id=243850]

#### Material Examined.

Holotype, female: **MADAGASCAR:** Fianarantsoa Auto. Prov., rainforest, 36km S Ambalavao, 22°12'S 46°58'E, 23.X.1993, Berlese funnel, B. L. Fisher, OSUC 181021 (deposited in CASC). Paratypes: **MADAGASCAR:** 3 females, CASENT 2135903, OSUC 181022, OSUC 181023 (CASC).

### Trichoteleia pauliani

(Risbec)

urn:lsid:zoobank.org:act:E584F9D9-5C8F-4868-9860-83B0902D4505

urn:lsid.biosci.ohio-state.edu:osuc_concepts: 5508

Figures 4 198–203 Morphbank ^31^

Alloteleia pauliani : Risbec 1956: 255. Original description.Trichoteleia pauliani: Masner 1976: 41. Generic transfer.

#### Description.

##### Female body length:

2.99–3.25 mm (n=5). Male body length: 2.60–3.16 mm (n=19). Color of head: dark brown to black; yellow, becoming darker dorsally; yellow throughout. Central keel of frons: absent; present, ending above interantennal process. Sculpture of medial frons in female: smooth. Sculpture of medial frons in male: smooth. Number of mandibular teeth: three. Basal node on mandible: absent. Sculpture of frons below median ocellus: densely punctate throughout. Sculpture of posterior vertex: densely punctate; moderately punctate medially, densely punctate laterally. Occipital rim: comprised of small to miniscule cells. Sculpture of gena: punctate rugose; dorsoventrally strigose. Basiconic sensillum on A7: absent.

##### Color of mesosoma in female:

dark brown to black; yellow throughout. Color of mesosoma in male: dark brown to black; variably yellow to brown. Sculpture along posterior pronotal sulcus: striate, striae well defined. Notaulus: smooth furrow incomplete, reaching suprahumeral sulcus as row of punctures. Sculpture of medial mesoscutum: moderately punctate in posterior half, becoming denser anteriorly. Sculpture of mesoscutellum: smooth with sparse fine punctures throughout; smooth medially, sparsely punctate laterally. Postacetabular sulcus: comprised of small cells. Mesopleural carina: absent; present. Sculpture along ventral half of prespecular sulcus: punctate. Sculpture of posterolateral mesepisternum: smooth; smooth except for 1 or 2 rows of fine punctures parallel to mesopleural carina. Sculpture of ventral surface of mesepisternum: smooth. Setation of ventral metapleural area: absent. Setation of metapleural triangle: absent; sparse. Sculpture of metapleural triangle: smooth; finely punctate. Posterior margin of metapleuron below propodeal spiracle: straight to moderately convex; with blunt kink near intersection with metapleural sulcus. Color of legs: yellow throughout; pale brown throughout.

##### Color of metasoma in female:

dark brown to black throughout; T1, medial T4 brown, otherwise yellow. Color of metasoma in male: pale to dark brown throughout; dark brown to black throughout. Posterior margin of transverse sulcus on T2: strongly convex. Sublateral tergal carina on T2: absent. Microsculpture on T2: present. Microsculpture on T3: present. Microsculpture on T4: present. Horn on T1 in female: present as a large, apically rounded protuberance. Macrosculpture of T2 in female: weakly longitudinally striate throughout. Macrosculpture of medial T3 in female: absent. Macrosculpture of lateral T3 in female: absent. Macrosculpture of medial T4 in female: absent. Macrosculpture of lateral T4 in female: absent. Punctation of T4 in female: sparse in medial third, moderately dense laterally; absent in medial third, sparse laterally. Macrosculpture of T5 in female: absent. Punctation of T5 in female: moderately dense throughout. Shape of T5 in female: width of posterior margin greater than or equal to length. Microscupture on T6 in female: absent. Sculpture of T6 in female: densely and finely punctate. Macrosculpture of T2 in male: longitudinally striate throughout; weakly longitudinally striate throughout. Macrosculpture of medial T3 in male: absent. Macrosculpture of lateral T3 in male: absent. Macrosculpture of T4 in male: absent. Punctation of T4 in male: sparse in medial third, moderately dense laterally; sparse throughout. Macrosculpture of T5 in male: absent. Punctation of T5 in male: sparse in medial third, dense laterally; sparse in medial third, moderately dense laterally. Sculpture of S2: crenulate along anterior margin, otherwise smooth with small punctures throughout. Prominent longitudinal median carina on S2: absent.

##### Wings:

macropterous, apex or forewing extending beyond posterior margin of T3. Color of forewing in female: slightly infuscate with longitudinal dark streak. Color of forewing in male: slightly infuscate throughout; slightly infuscate with longitudinal dark streak. Color of hind wing: hyaline throughout; hyaline with faint infuscate patches posterior to R and along anterior margin apical to R. Density of setation in fore wing: uniform throughout. Density of setation in hind wing: uniform throughout. Length of R1: more than 1.5 times as long as r. M+Cu and RS+M in forewing: nebulous.

**Figures 198–203.**
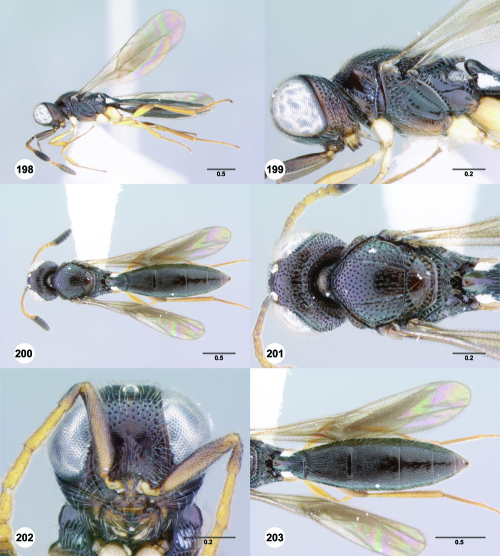
^100^ Trichoteleia pauliani sp. n. **198** Lateral habitus, female (CASENT 2134096) **199** Head and mesosoma, lateral view, female (CASENT 2134096) **200** Dorsal habitus, female (CASENT 2134096) **201** Head and mesosoma, dorsal view, female (CASENT 2134096) **202** Head, anterior view, female (CASENT 2134091) **203** Metasoma, dorsal view, female (CASENT 2134096). Scale bars in millimeters.

#### Diagnosis.

This species is similar to Trichoteleia hemlyae and Trichoteleia ravaka. It can be separated by its lack of a well-developed central keel on the frons (Figs 4, 202) and the smooth to sparsely and finely punctate metapleural triangle (Fig. 199).

#### Link to Distribution Map.

[http://hol.osu.edu/map-large.html?id=243414]

#### Material Examined.

Lectotype, female: **MADAGASCAR:** Antsiranana Auto. Prov., forest moss, Mount Maromokotro (Tsaratanana), 1500–1800m, X–1949, R. Paulian, MNHN 0030 (deposited in MNHN). Other material: **MADAGASCAR:** 6 females, 18 males, CASENT 2043796, 2118412, 2134093, 2134096, 2135789, 2135794, 2135807, 2135812, 2135827, 2135902, 2136139, 2136148, 2136164, 2136418, OSUC 181016, OSUC 215779 (CASC); OSUC 181017, 181063 (CNCI); CASENT 2134091, 2135811, 2135826, 2136005, 2136149, 2136193 (OSUC).

### Trichoteleia picturata

Talamas sp. n.

urn:lsid:zoobank.org:act:BE93D63B-CBB3-449A-93C5-49AC57A88713

urn:lsid.biosci.ohio-state.edu:osuc_concepts:243412

Figures 18 33 204–209 Morphbank ^32^ 

#### Description.

##### Female body length:

2.83 mm (n=1). Color of head: pale brown, becoming darker at vertex. Central keel of frons: present, extending onto interantennal process. Sculpture of medial frons in female: smooth. Number of mandibular teeth: three. Basal node on mandible: present. Sculpture of frons below median ocellus: punctate rugulose throughout. Sculpture of posterior vertex: punctate rugulose. Occipital rim: comprised of small to miniscule cells. Sculpture of gena: dorsoventrally strigose.

##### Basiconic sensillum on A7:

absent.

##### Color of mesosoma in female:

variably orange to brown. Sculpture along posterior pronotal sulcus: striate, striae well defined. Notaulus: percurrent, reaching suprahumeral sulcus as a smooth furrow. Sculpture of medial mesoscutum: longitudinally rugulose posteriorly, transversely rugulose anteriorly. Sculpture of mesoscutellum: smooth medially, coarsely punctate laterally. Postacetabular sulcus: present as a smooth furrow. Mesopleural carina: present. Sculpture along ventral half of prespecular sulcus: punctate rugose. Sculpture of posterolateral mesepisternum: smooth. Sculpture of ventral surface of mesepisternum: smooth. Setation of ventral metapleural area: absent. Setation of metapleural triangle: sparse. Sculpture of metapleural triangle: rugulose. Posterior margin of metapleuron below propodeal spiracle: with blunt kink near intersection with metapleural sulcus. Color of legs: coxae and trochanters yellow, otherwise pale brown, hindlegs the darkest.

##### Color of metasoma in female:

yellow, posterior corners of T2–T4 dark brown. Posterior margin of transverse sulcus on T2: strongly convex. Sublateral tergal carina on T2: absent. Microsculpture on T2: absent. Microsculpture on T3: absent. Microsculpture on T4: absent. Horn on T1 in female: present as a large, apically rounded protuberance. Macrosculpture of T2 in female: longitudinally striate throughout. Macrosculpture of medial T3 in female: absent. Macrosculpture of lateral T3 in female: weakly longitudinally striate. Macrosculpture of medial T4 in female: absent. Macrosculpture of lateral T4 in female: obliquely strigose. Punctation of T4 in female: sparse in medial third, moderately dense laterally. Macrosculpture of T5 in female: absent. Punctation of T5 in female: sparse in medial third, moderately dense laterally. Shape of T5 in female: width of posterior margin greater than or equal to length. Microscupture on T6 in female: absent. Sculpture of T6 in female: smooth with fine setigerous punctures along lateral margin. Sculpture of S2: longitudinally striate anteriorly, smooth posteriorly. Prominent longitudinal median carina on S2: absent.

##### Wings:

macropterous, apex or forewing extending beyond posterior margin of T3. Color of forewing in female: infuscate in apical two-thirds, white spot near apex, two white patches medially at anterior and posterior margins. Color of hind wing: hyaline throughout. Density of setation in fore wing: uniform throughout. Density of setation in hind wing: uniform throughout. Length of R1: more than 1.5 times as long as r. M+Cu and RS+M in forewing: nebulous.

**Figures 204–209.**
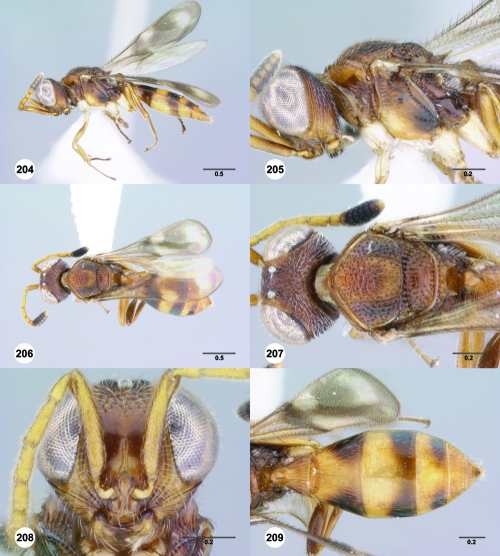
^101^Trichoteleia picturata sp. n., female holotype (CASENT 2135904). **204** Lateral habitus **205** Head and mesosoma, lateral view **206** Dorsal habitus **207** Head and mesosoma, dorsal view **208** Head, anterior view **209** Metasoma, dorsal view. Scale bars in millimeters.

#### Diagnosis.

Trichoteleia picturata is similar to Trichoteleia cincta, Trichoteleia delilah, and Trichoteleia zuparkoi and shares with them the pattern of coloration on the forewings. It can be separated from Trichoteleia cincta by the punctate rugulose sculpture below the median ocellus (Fig. 208), from Trichoteleia delilah by the absence of a basiconic sensillum on A7, and from Trichoteleia zuparkoi by longitudinally rugulose sculpture in the posterior half of the medial mesoscutum (Fig. 207).

#### Etymology.

The adjectival epithet *picturata*, meaning “painted”, refers to the color pattern of the forewing in this species.

#### Link to Distribution Map.

[http://hol.osu.edu/map-large.html?id=243412]

#### Material Examined.

Holotype, female: **MADAGASCAR:** Antsiranana Auto. Prov., 20.4km (219°) SW Antanambao, leaf mold / rotten wood / rainforest, #1990, Manongarivo Special Reserve, 14°02.8'S 48°24.1'E, 1860m, 3.XI.1998, sifted litter, B. L. Fisher, CASENT 2135904 (deposited in CASC).

### Trichoteleia prima

Talamas sp. n.

urn:lsid:zoobank.org:act:E9611F48-6B5E-441D-A372-FECA63C75DEE

urn:lsid.biosci.ohio-state.edu:osuc_concepts:241276

Figures 11 210–215 Morphbank ^33^ 

#### Description.

##### Female body length:

4.01–4.45 mm (n=3). Color of head: orange throughout; dark red, becoming darker dorsally. Central keel of frons: present, extending onto interantennal process. Sculpture of medial frons in female: smooth. Number of mandibular teeth: three. Basal node on mandible: absent. Sculpture of frons below median ocellus: finely punctate throughout, surface uneven. Sculpture of posterior vertex: moderately punctate. Occipital rim: comprised of small to miniscule cells. Sculpture of gena: dorsoventrally strigose. Basiconic sensillum on A7: absent.

##### Color of mesosoma in female:

orange throughout; variably red to black. Sculpture along posterior pronotal sulcus: striate, striae well defined. Notaulus: percurrent, reaching suprahumeral sulcus as a smooth furrow. Sculpture of medial mesoscutum: moderately punctate in posterior half, becoming denser anteriorly. Sculpture of mesoscutellum: smooth medially, sparsely punctate laterally. Postacetabular sulcus: comprised of small cells. Mesopleural carina: present. Sculpture along ventral half of prespecular sulcus: finely punctate. Sculpture of posterolateral mesepisternum: smooth. Sculpture of ventral surface of mesepisternum: smooth. Setation of ventral metapleural area: absent. Setation of metapleural triangle: sparse; moderately dense. Sculpture of metapleural triangle: rugulose. Posterior margin of metapleuron below propodeal spiracle: straight to moderately convex. Color of legs: yellow throughout.

##### Color of metasoma in female:

dark brown to black throughout. Posterior margin of transverse sulcus on T2: strongly convex. Sublateral tergal carina on T2: absent. Microsculpture on T2: present. Microsculpture on T3: present. Microsculpture on T4: absent. Horn on T1 in female: present as a large, apically rounded protuberance. Macrosculpture of T2 in female: longitudinally striate throughout; longitudinally strigose throughout. Macrosculpture of medial T3 in female: longitudinally striate; rugulose. Macrosculpture of lateral T3 in female: longitudinally striate; longitudinally strigose. Macrosculpture of medial T4 in female: absent. Macrosculpture of lateral T4 in female: longitudinally strigose. Punctation of T4 in female: sparse in medial third, dense laterally. Macrosculpture of T5 in female: absent; longitudinally strigose laterally. Punctation of T5 in female: dense throughout; sparse along midline, otherwise dense. Shape of T5 in female: width of posterior margin less than length. Microscupture on T6 in female: present along anterior margin. Sculpture of T6 in female: finely rugulose along anterior margin, otherwise smooth with sparse punctation. Sculpture of S2: longitudinally striate medially, punctate interstitially. Prominent longitudinal median carina on S2: absent.

##### Wings:

macropterous, apex or forewing extending beyond posterior margin of T3. Color of forewing in female: slightly infuscate throughout. Color of hind wing: slightly infuscate throughout. Density of setation in fore wing: uniform throughout. Density of setation in hind wing: uniform throughout. Length of R1: more than 1.5 times as long as r. M+Cu and RS+M in forewing: nebulous.

**Figures 210–215.**
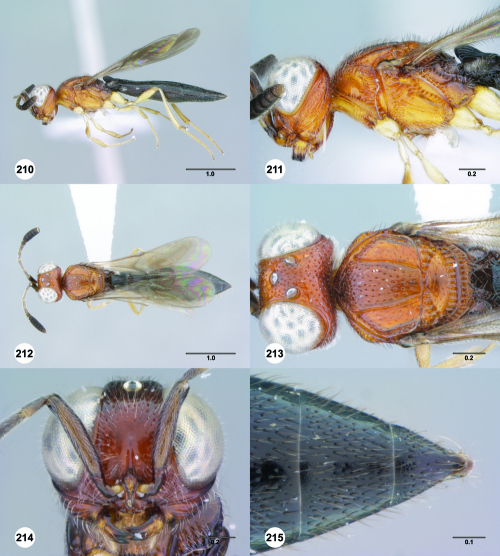
^102^Trichoteleia prima sp. n. **210** Lateral habitus, female holotype (CASENT 2043321) **211** Head and mesosoma, lateral view, female holotype (CASENT 2043321) **212** Dorsal habitus, female holotype (CASENT 2043321) **213** Head and mesosoma, dorsal view, female holotype (CASENT 2043321) **214** Head, anterior view, female (CASENT 2134201) **215** T5–T6, dorsal view, female (CASENT 2134201). Scale bars in millimeters.

#### Diagnosis.

Trichoteleia prima is most similar to Trichoteleia levii and Trichoteleia tonsa. In these three species the short striae along the pronotal shoulder and those of the lateral pronotum form a continuous band. Trichoteleia prima is separated from the others in this cluster by the combination of a coarsely sculptured metapleural triangle and an absence of microsculpture in the posterior half of T6.

#### Etymology.

This species was the first that the first author examined during this revision. The adjectival epithet *prima*, meaning “first,” reflects this.

#### Link to Distribution Map.

[http://hol.osu.edu/map-large.html?id=241276]

#### Material Examined.

Holotype, female: **MADAGASCAR:** Antsiranana Auto. Prov., MA-01-01D-08, Montagne d’Ambre National Park, 12°31'13"S, 49°10'45"E, 1125m, 21.IV–26.IV.2001, malaise trap, R. Harin’Hala, CASENT 2043321 (deposited in CASC). Paratypes: **MADAGASCAR:** 4 females, CASENT 2008744, 2008754, 2134201 (CASC); OSUC 215614 (OSUC).

### Trichoteleia prolixa

Talamas sp. n.

urn:lsid:zoobank.org:act:94B84B17-EA48-4A3C-95A4-D1BF227C9477

urn:lsid:biosci.ohio-state.edu:osuc_concepts:253566

**Figures 216–220.**
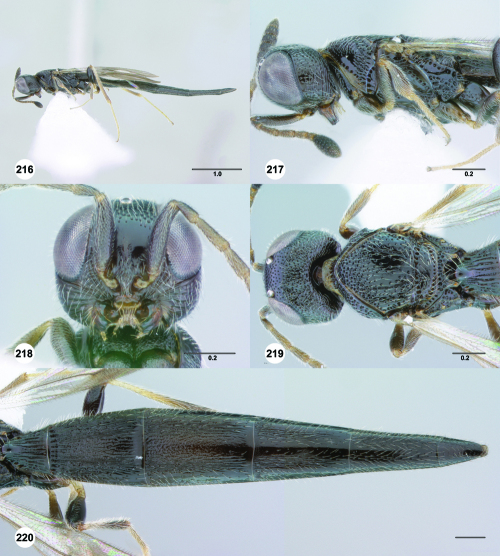
^103^Trichoteleia prolixa sp. n., female holotype (OSUC 254609). **216** Lateral habitus **217** Head and mesosoma, lateral view **218** head, anterior view **219** Head and mesosoma, dorsal view **220** Metasoma, dorsal view. Scale bars in millimeters.

Figures 216–220 Morphbank ^34^ 

#### Description.

##### Female Anterior width of T5:

0.31 mm (n=1). Female body length: 4.55 mm (n=1). Female Length of t5 in female: 0.62 mm (n=1). Female Posteroir width of T5: 0.21 mm (n=1). Color of head: black throughout. Central keel of frons: present, bifurcating ventrally around interantennal process. Sculpture of medial frons in female: smooth. Number of mandibular teeth: two. Basal node on mandible: absent. Sculpture of frons below median ocellus: densely punctate throughout. Sculpture of posterior vertex: densely punctate. Sculpture of gena: dorsoventrally strigose. Basiconic sensillum on A7: absent.

##### Color of mesosoma in female:

dark brown to black. Sculpture along posterior pronotal sulcus: striate, striae short and poorly defined. Notaulus: smooth furrow incomplete, reaching suprahumeral sulcus as row of punctures. Sculpture of medial mesoscutum: smooth and sparsely punctate posteriorly, densely punctate and transverely rugulose anteriorly. Sculpture of mesoscutellum: smooth medially, sparsely punctate laterally. Sculpture along ventral half of prespecular sulcus: coarsely punctate. Sculpture of posterolateral mesepisternum: coarsely punctate. Setation of ventral metapleural area: absent. Setation of metapleural triangle: sparse. Sculpture of metapleural triangle: punctate rugose. Posterior margin of metapleuron below propodeal spiracle: straight to moderately convex. Color of legs: coxae and femora dark brown, trochanters, tibiae and tarsi pale brown to yellow.

##### Color of metasoma in female:

dark brown to black throughout. Posterior margin of transverse sulcus on T2: weakly convex. Sublateral tergal carina on T2: absent. Microsculpture on T2: present. Microsculpture on T3: present. Microsculpture on T4: present. Horn on T1 in female: present as a large, apically rounded protuberance. Macrosculpture of T2 in female: longitudinally strigose throughout. Macrosculpture of medial T3 in female: rugulose. Macrosculpture of lateral T3 in female: longitudinally strigose. Macrosculpture of medial T4 in female: absent. Macrosculpture of lateral T4 in female: longitudinally strigose. Punctation of T4 in female: sparse in medial third, moderately dense laterally. Macrosculpture of T5 in female: absent. Punctation of T5 in female: moderately dense throughout. Shape of T5 in female: width of posterior margin less than length. Microscupture on T6 in female: absent. Sculpture of T6 in female: smooth with fine setigerous punctures laterally. Sculpture of S2: coarsely punctate throughout. Prominent longitudinal median carina on S2: absent.

##### Wings:

macropterous, apex or forewing extending beyond posterior margin of T3. Color of forewing in female: hyaline with infuscate patch anad of R. Color of hind wing: hyaline throughout. Density of setation in fore wing: uniform throughout. Density of setation in hind wing: uniform throughout. Length of R1: more than 1.5 times as long as r. M+Cu and RS+M in forewing: nebulous.

#### Diagnosis.

The combination of a conspicuously elongate metasoma and bidentate mandibles renders this species rather straightforward to identify.

#### Etymology.

The adjectival epithet *prolixa*, meaning “stretched out” or “long” in Latin, refers to the length of the metasoma in this species.

#### Link to Distribution Map.

[http://hol.osu.edu/map-large.html?id=253566]

#### Material Examined.

Holotype, female: **MADAGASCAR:** Mahajanga Auto. Prov., Belambo Commune, 20km NW Boriziny (Port-Bergé), secondary growth / white sand, MG-33-01, Ambovomamy, 15°27.07'S 47°36.80'E, 33m, 4.I–14.I.2007, malaise trap, R. Harin’Hala, M. Irwin & F. Parker, OSUC 254609 (deposited in CASC).

### Trichoteleia quazii

Talamas sp. n.

urn:lsid:zoobank.org:act:66A7D751-B50A-4EB0-99CA-437B503B34FF

urn:lsid.biosci.ohio-state.edu:osuc_concepts:241270

Figures 32 221–226 Morphbank ^35^ 

#### Description.

##### Female body length:

4.74–5.08 mm (n=4). Male body length: 4.07 mm (n=1). Color of head: dark brown to black. Central keel of frons: present, bifurcating ventrally around interantennal process. Sculpture of medial frons in female: smooth. Sculpture of medial frons in male: smooth. Number of mandibular teeth: three. Basal node on mandible: absent. Sculpture of frons below median ocellus: densely punctate throughout. Sculpture of posterior vertex: densely punctate. Occipital rim: comprised of medium to large sized cells. Sculpture of gena: punctate rugose; dorsoventrally strigose. Basiconic sensillum on A7: absent.

##### Color of mesosoma in female:

dark brown to black. Color of mesosoma in male: dark brown to black. Sculpture along posterior pronotal sulcus: striate, striae well defined. Notaulus: smooth furrow incomplete, reaching suprahumeral sulcus as row of punctures. Sculpture of medial mesoscutum: moderately punctate in posterior half, becoming denser anteriorly. Sculpture of mesoscutellum: smooth medially, sparsely punctate laterally. Postacetabular sulcus: comprised of small cells. Mesopleural carina: present. Sculpture along ventral half of prespecular sulcus: punctate. Sculpture of posterolateral mesepisternum: punctate. Sculpture of ventral surface of mesepisternum: finely punctate. Setation of ventral metapleural area: absent. Setation of metapleural triangle: sparse. Sculpture of metapleural triangle: punctate rugose. Posterior margin of metapleuron below propodeal spiracle: straight to moderately convex. Color of legs: coxae and trochanters yellow, otherwise brown, becoming paler distally.

##### Color of metasoma in female:

dark brown to black throughout. Color of metasoma in male: dark brown to black throughout. Posterior margin of transverse sulcus on T2: strongly convex. Sublateral tergal carina on T2: absent. Microsculpture on T2: present. Microsculpture on T3: present. Microsculpture on T4: present. Horn on T1 in female: present as a large, apically rounded protuberance. Macrosculpture of T2 in female: longitudinally strigose throughout. Macrosculpture of medial T3 in female: reticulate rugose. Macrosculpture of lateral T3 in female: longitudinally strigose. Macrosculpture of medial T4 in female: absent. Macrosculpture of lateral T4 in female: absent; weakly rugulose. Punctation of T4 in female: dense throughout; sparse along midline, otherwise dense. Macrosculpture of T5 in female: absent. Punctation of T5 in female: dense throughout. Shape of T5 in female: width of posterior margin less than length. Microscupture on T6 in female: present along anterior margin. Sculpture of T6 in female: densely and finely punctate. Macrosculpture of T2 in male: longitudinally strigose. Macrosculpture of medial T3 in male: weakly rugulose. Macrosculpture of lateral T3 in male: longitudinally strigose. Macrosculpture of T4 in male: absent. Punctation of T4 in male: sparse along midline, otherwise dense. Macrosculpture of T5 in male: absent. Punctation of T5 in male: sparse along midline, otherwise dense throughout. Sculpture of S2: coarsely punctate throughout. Prominent longitudinal median carina on S2: present.

##### Wings:

macropterous, apex or forewing extending beyond posterior margin of T3. Color of forewing in female: infuscate throughout with metallic purple sheen along veins. Color of forewing in male: infuscate throughout with metallic purple sheen along veins. Color of hind wing: slightly infuscate throughout. Density of setation in fore wing: uniform throughout. Density of setation in hind wing: uniform throughout. Length of R1: more than 1.5 times as long as r. M+Cu and RS+M in forewing: nebulous.

**Figures 221–226.**
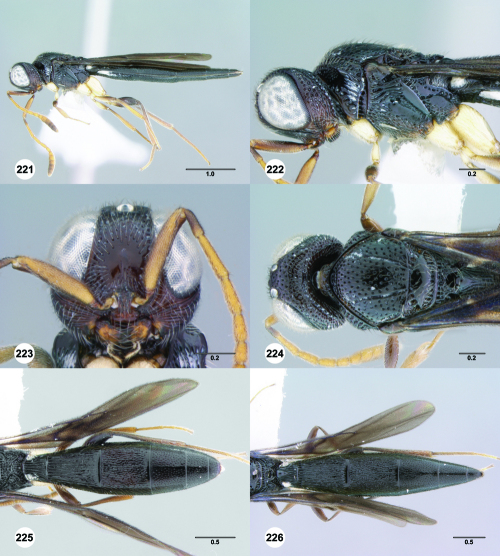
^104^Trichoteleia quazii sp. n. **221** Lateral habitus, female holotype (CASENT 2132026) **222** Head and mesosoma, lateral view, female holotype (CASENT 2132026) **223** Head, anterior view, female holotype (CASENT 2132026) **224** Head and mesosoma, dorsal view, female holotype (CASENT 2132026) **225** Metasoma, dorsal view, male (CASENT 2043792) **226** Metasoma, dorsal view, female (CASENT 2043798). Scale bars in millimeters.

#### Diagnosis.

Trichoteleia quazii is similar to Trichoteleia longiventris and Trichoteleia carinata in the elongate shape and dark color of the body. Trichoteleia quazii shares metallic purple sheen along the forewing veins with Trichoteleia longiventris; the presence of a longitudinal medial carina on S2 in Trichoteleia quazii (as in Fig. 45) separates it. Trichoteleia carinata bears a prominent longitudinal carina on lateral T2 (Fig. 84); Trichoteleia quazii does not.

#### Etymology.

Trichoteleia quazii is named for James R. Quazi (USA), a friend of the first author, to commemorate his wedding to Inga Sheffield (Mexico).

#### Link to Distribution Map.

[http://hol.osu.edu/map-large.html?id=241270]

#### Material Examined.

Holotype, female: **MADAGASCAR:** Fianarantsoa Auto. Prov., radio tower, forest edge / mixed tropical forest, MA-02-09B-61, Ranomafana National Park, 21°15.05'S 47°24.43'E, 1130m, 17.V–30.V.2003, malaise trap, R. Harin’Hala, CASENT 2132026 (deposited in CASC). Paratypes: **MADAGASCAR:** 3 females, 1 male, CASENT 2043792, 2043798, 2134082 (CASC); CASENT 2043941 (OSUC).

### Trichoteleia ravaka

Talamas & Masner sp. n.

urn:lsid:zoobank.org:act:BAC8D487-65B7-4F9E-B133-344C541A2CF5

urn:lsid.biosci.ohio-state.edu:osuc_concepts: 249112

**Figures 227–232.**
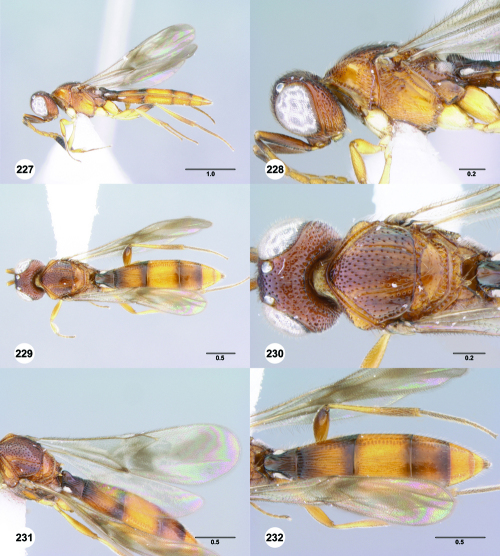
^105^Trichoteleia ravaka sp. n., female holotype (CASENT 2135788). **227** Lateral habitus **228** Head and mesosoma, lateral view **229** Dorsal habitus **230** Head and mesosoma, dorsal view **231** Fore and hind wing, dorsal view **232** Metasoma, dorsal view. Scale bars in millimeters.

Figures 227–232 Morphbank ^36^ 

#### Description.

##### Female body length:

3.72 mm (n=1). Color of head: dark orange, becoming brown at vertex. Central keel of frons: present, extending onto interantennal process. Sculpture of medial frons in female: smooth. Number of mandibular teeth: three. Basal node on mandible: absent. Sculpture of frons below median ocellus: densely punctate throughout. Sculpture of posterior vertex: moderately punctate. Occipital rim: comprised of medium to large sized cells. Sculpture of gena: dorsoventrally strigose. Basiconic sensillum on A7: absent.

##### Color of mesosoma in female:

variably orange to brown. Sculpture along posterior pronotal sulcus: striate, striae well defined. Notaulus: smooth furrow incomplete, reaching suprahumeral sulcus as row of punctures. Sculpture of medial mesoscutum: moderately punctate in posterior half, becoming denser anteriorly. Sculpture of mesoscutellum: smooth with sparse fine punctures throughout. Postacetabular sulcus: comprised of small cells. Mesopleural carina: absent. Sculpture along ventral half of prespecular sulcus: punctate. Sculpture of posterolateral mesepisternum: smooth except for 1 or 2 rows of fine punctures parallel to mesopleural carina. Sculpture of ventral surface of mesepisternum: smooth. Setation of ventral metapleural area: absent. Setation of metapleural triangle: sparse. Sculpture of metapleural triangle: rugulose. Posterior margin of metapleuron below propodeal spiracle: straight to moderately convex. Color of legs: apical hind femur and tibia, all tarsi brown, legs otherwise yellow.

##### Color of metasoma in female:

T1, junctions of tergites brown, otherwise orange. Posterior margin of transverse sulcus on T2: strongly convex. Sublateral tergal carina on T2: absent. Microsculpture on T2: present. Microsculpture on T3: present. Microsculpture on T4: present. Horn on T1 in female: present as a large, apically rounded protuberance. Macrosculpture of T2 in female: longitudinally striate throughout. Macrosculpture of medial T3 in female: longitudinally strigose. Macrosculpture of lateral T3 in female: longitudinally strigose. Macrosculpture of medial T4 in female: absent. Macrosculpture of lateral T4 in female: weakly longitudinally strigose. Punctation of T4 in female: sparse throughout. Macrosculpture of T5 in female: absent. Punctation of T5 in female: sparse throughout. Shape of T5 in female: width of posterior margin greater than or equal to length. Microscupture on T6 in female: absent. Sculpture of T6 in female: finely punctate throughout. Sculpture of S2: longitudinally striate anteriorly, smooth posteriorly. Prominent longitudinal median carina on S2: absent.

##### Wings:

macropterous, apex or forewing extending beyond posterior margin of T3. Color of forewing in female: infuscate in apical two-thirds, with white patches along anterior and posterior margins. Color of hind wing: slightly infuscate throughout. Density of setation in fore wing: uniform throughout. Density of setation in hind wing: uniform throughout. Length of R1: more than 1.5 times as long as r. M+Cu and RS+M in forewing: nebulous.

#### Diagnosis.

This species is similar to Trichoteleia hemlyae and Trichoteleia pauliani. It differs from Trichoteleia pauliani by having a central keel (as in Figs 3, 130) and a punctate to rugulose metapleural triangle (Fig. 228), and from Trichoteleia hemlyae by the presence of banding in the forewing (Fig. 231). Color patterns of the body in this species group are particularly plastic and should be avoided during identification.

#### Etymology.

The word *ravaka* means “jewel” in Malagasy and is used as a noun in apposition in reference to the beauty and rarity of this species.

#### Link to Distribution Map.

[http://hol.osu.edu/map-large.html?id=249112]

#### Material Examined.

Holotype, female: **MADAGASCAR:** Toamasina Auto. Prov., botanical garden nr. entrance, tropical forest, MA-01-08B-15, Mantadia (Andasibe) National Park, 18°55.58'S 48°24.47'E, 1025m, 8.X–16.X.2001, malaise trap, R. Harin’Hala, CASENT 2135788 (deposited in CASC).

### Trichoteleia rugifrons

Talamas & Masner sp. n.

urn:lsid:zoobank.org:act:03916211-7889-4831-A70C-6EAF97AE9794

urn:lsid:biosci.ohio-state.edu:osuc_concepts:238242

Figures 39 233–238 Morphbank ^37^ 

#### Description.

##### Female body length:

2.45–3.26 mm (n=18). Male body length: 2.65–2.92 mm (n=14). Color of head: dark brown to black. Central keel of frons: present, extending onto interantennal process. Sculpture of medial frons in female: smooth. Sculpture of medial frons in male: smooth. Number of mandibular teeth: three. Basal node on mandible: present. Sculpture of frons below median ocellus: finely punctate throughout, dorsoventrally strigose laterally; dorsoventrally strigose throughout. Sculpture of posterior vertex: moderately punctate, rugose posterior to eyes and posterior ocellus. Occipital rim: comprised of medium to large sized cells. Sculpture of gena: dorsoventrally strigose. Basiconic sensillum on A7: absent.

##### Color of mesosoma in female:

dark brown to black; reddish brown throughout. Color of mesosoma in male: dark brown to black. Sculpture along posterior pronotal sulcus: striate, striae well defined; striate, striae short and poorly defined. Notaulus: percurrent, reaching suprahumeral sulcus as a smooth furrow. Sculpture of medial mesoscutum: moderately punctate in posterior half, becoming denser anteriorly. Sculpture of mesoscutellum: smooth with sparse fine punctures throughout; smooth medially, coarsely punctate laterally; smooth medially, sparsely punctate laterally. Postacetabular sulcus: comprised of small cells. Mesopleural carina: present. Sculpture along ventral half of prespecular sulcus: coarsely punctate. Sculpture of posterolateral mesepisternum: smooth. Sculpture of ventral surface of mesepisternum: smooth. Setation of ventral metapleural area: absent. Setation of metapleural triangle: sparse. Sculpture of metapleural triangle: punctate rugose. Posterior margin of metapleuron below propodeal spiracle: with blunt kink near intersection with metapleural sulcus. Color of legs: yellow throughout.

##### Color of metasoma in female:

dark brown to black throughout; reddish brown. Color of metasoma in male: pale to dark brown throughout; reddish brown. Posterior margin of transverse sulcus on T2: strongly convex. Sublateral tergal carina on T2: absent. Microsculpture on T2: absent. Microsculpture on T3: absent. Microsculpture on T4: absent. Horn on T1 in female: present as a large, apically rounded protuberance. Macrosculpture of T2 in female: longitudinally striate throughout. Macrosculpture of medial T3 in female: weakly longitudinally striate; absent; longitudinally striate. Macrosculpture of lateral T3 in female: longitudinally striate. Macrosculpture of medial T4 in female: absent. Macrosculpture of lateral T4 in female: punctate rugulose; longitudinally strigose. Punctation of T4 in female: absent in medial third, moderately dense laterally. Macrosculpture of T5 in female: punctate rugulose throughout; punctate rugulose laterally. Punctation of T5 in female: moderately dense throughout; absent along midline, otherwise moderately dense. Shape of T5 in female: width of posterior margin greater than or equal to length. Microscupture on T6 in female: absent. Sculpture of T6 in female: punctate rugulose. Macrosculpture of T2 in male: longitudinally striate throughout. Macrosculpture of medial T3 in male: longitudinally striate. Macrosculpture of lateral T3 in male: longitudinally striate. Macrosculpture of T4 in male: longitudinally strigose laterally; punctate rugulose laterally. Punctation of T4 in male: sparse in medial third, moderately dense laterally; sparse throughout; absent in medial third, sparse laterally. Macrosculpture of T5 in male: punctate crenulate. Punctation of T5 in male: absent along midline, otherwise moderately dense throughout. Sculpture of S2: longitudinally striate medially with sparse fine punctation. Prominent longitudinal median carina on S2: absent.

##### Wings:

macropterous, apex or forewing extending beyond posterior margin of T3. Color of forewing in female: infuscate throughout; slightly infuscate throughout. Color of forewing in male: slightly infuscate throughout. Color of hind wing: slightly infuscate throughout. Density of setation in fore wing: uniform throughout. Density of setation in hind wing: uniform throughout. Length of R1: more than 1.5 times as long as r. M+Cu and RS+M in forewing: nebulous.

**Figures 233–238.**
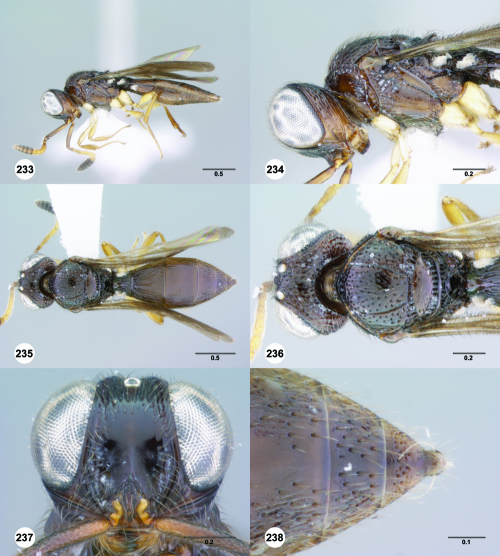
^106^Trichoteleia rugifrons sp. n. **233** Lateral habitus, female (CASENT 2118419) **234** Head and mesosoma, lateral view, female (CASENT 2118419) **235** Dorsal habitus, female (CASENT 2118419) **236** Head and mesosoma, dorsal view, female (CASENT 2118419) **237** Head, anterior view, female (CASENT 2042871) **238** T5**–**T6, dorsal view, female (CASENT 2118419). Scale bars in millimeters.

#### Diagnosis.

This species is most similar to Trichoteleia solocis and Trichoteleia warreni. Trichoteleia warreni has evenly striate sculpture immediately below the median ocellus (Fig. 291), whereas the striae in Trichoteleia rugifrons and Trichoteleia solocis attenuate medially. Trichoteleia solocis is entirely yellow and has weak rugae laterad of the median ocellus (Fig. 243). In Trichoteleia rugifrons the rugae laterad of the median ocellus are prominent (Fig. 237) and the specimens are brown to black.

#### Etymology.

The name *rugifrons,* a noun in apposition, refers to the coarse sculpture of the upper frons.

#### Link to Distribution Map.

[http://hol.osu.edu/map-large.html?id=238242]

#### Material Examined.

Holotype, female: **MADAGASCAR:** Fianarantsoa Auto. Prov., rainforest, 7km W Ranomafana, 21°16'S 47°25'E, 1000m, 10.IX–22.IX.1993, pan trap, W. Steiner, OSUC 181042 (deposited in CNCI). Paratypes: **MADAGASCAR:** 18 females, 14 males, CASENT 2042871, 2118419, 2134777, 2135938–2135939, 2135945–2135946, 2135952–2135953, 2135955, 2135957, 2136015, 2136162, 2137894, OSUC 181039 (CASC); OSUC 181045, 181048–181049 (CNCI); CASENT 2135760, 2135940, 2135958, 2136157 (OSUC); OSUC 181040–181041, 181043–181044, 181047, 181050, 223959–223960, 241855, 241857 (USNM). Other material: **MADAGASCAR:** 1 male, 1 unknown, OSUC 265214, 266092 (OSUC).

### Trichoteleia solocis

Talamas sp. n.

urn:lsid:zoobank.org:act:0E94B844-AD89-4400-A11D-16EAB505E4B4

urn:lsid.biosci.ohio-state.edu:osuc_concepts:248829

**Figures 239–244.**
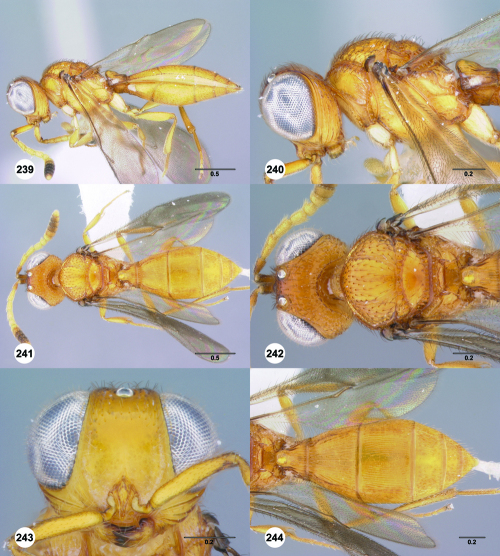
^107^Trichoteleia solocis sp. n. **239** Lateral habitus, female (CASENT 2135949) **240** Head and mesosoma, lateral view, female (CASENT 2135949) **241** Dorsal habitus, female holotype (CASENT 2135951) **242** Head and mesosoma, dorsal view, female holotype (CASENT 2135951) **243** Head, anterior view, female (CASENT 2135949) **244** Metasoma, dorsal view, female holotype (CASENT 2135951). Scale bars in millimeters. Scale bars in millimeters.

Figures 239–244 Morphbank ^38^ 

#### Description.

##### Female body length:

2.90–3.02 mm (n=2). Color of head: yellow throughout. Central keel of frons: present, extending onto interantennal process. Sculpture of medial frons in female: smooth. Number of mandibular teeth: three. Basal node on mandible: present. Sculpture of frons below median ocellus: finely punctate throughout, dorsoventrally strigose laterally. Sculpture of posterior vertex: moderately punctate. Occipital rim: comprised of small to miniscule cells. Sculpture of gena: dorsoventrally strigose. Basiconic sensillum on A7: absent.

##### Color of mesosoma in female:

yellow throughout. Sculpture along posterior pronotal sulcus: striate, striae short and poorly defined; rugulose. Notaulus: percurrent, reaching suprahumeral sulcus as a smooth furrow. Sculpture of medial mesoscutum: moderately punctate throughout. Sculpture of mesoscutellum: smooth medially, sparsely punctate laterally. Postacetabular sulcus: comprised of small cells. Mesopleural carina: present. Sculpture along ventral half of prespecular sulcus: coarsely punctate. Sculpture of posterolateral mesepisternum: smooth. Sculpture of ventral surface of mesepisternum: smooth. Setation of ventral metapleural area: absent. Setation of metapleural triangle: sparse. Sculpture of metapleural triangle: punctate rugose. Posterior margin of metapleuron below propodeal spiracle: with blunt kink near intersection with metapleural sulcus. Color of legs: yellow throughout.

##### Color of metasoma in female:

yellow. Posterior margin of transverse sulcus on T2: strongly convex. Sublateral tergal carina on T2: absent. Microsculpture on T2: absent. Microsculpture on T3: absent. Microsculpture on T4: absent. Horn on T1 in female: present as a large, apically rounded protuberance. Macrosculpture of T2 in female: longitudinally striate throughout. Macrosculpture of medial T3 in female: longitudinally striate. Macrosculpture of lateral T3 in female: longitudinally striate. Macrosculpture of medial T4 in female: absent. Macrosculpture of lateral T4 in female: longitudinally strigose. Punctation of T4 in female: absent along midline, otherwise sparse. Macrosculpture of T5 in female: punctate rugulose laterally. Punctation of T5 in female: absent along midline, otherwise moderately dense. Shape of T5 in female: width of posterior margin greater than or equal to length. Microscupture on T6 in female: absent. Sculpture of T6 in female: moderately punctate throughout. Sculpture of S2: longitudinally striate anteromedially, otherwise smooth. Prominent longitudinal median carina on S2: absent.

##### Wings:

macropterous, apex or forewing extending beyond posterior margin of T3. Color of forewing in female: slightly infuscate throughout. Color of hind wing: slightly infuscate throughout. Density of setation in fore wing: uniform throughout. Density of setation in hind wing: uniform throughout. Length of R1: more than 1.5 times as long as r. M+Cu and RS+M in forewing: nebulous.

#### Diagnosis.

This species is most similar to Trichoteleia rugifrons and Trichoteleia warreni. It may be separated from Trichoteleia warreni by the smooth frons immediately below the median ocellus (Fig. 243) and from Trichoteleia rugifrons by its yellow color and weak rugae laterad of the median ocellus (Fig. 243).

#### Etymology.

The adjectival epithet *solocis*, meaning “coarse” or “rough” in Latin, refers to the sculpture of T6 in females of this species.

#### Link to Distribution Map.

[http://hol.osu.edu/map-large.html?id=248829]

#### Material Examined.

Holotype, female: **MADAGASCAR:** Toamasina Auto. Prov., 18km (21°) NNE Ambinanitelo, rainforest, BLF8003, Mount Anjanaharibe, 15°11'18"S, 49°36'54"E, 450m, 8.III–12.III.2003, pitfall trap, Fisher, Griswold et al., CASENT 2136151 (deposited in CASC). Paratype: **MADAGASCAR:** 1 female, CASENT 2135949 (CASC).

### Trichoteleia sphaerica

Talamas sp. n.

urn:lsid:zoobank.org:act:038D912F-CFA7-4804-89AE-23C3B2DA2C22

urn:lsid.biosci.ohio-state.edu:osuc_concepts:243954

**Figures 245–250.**
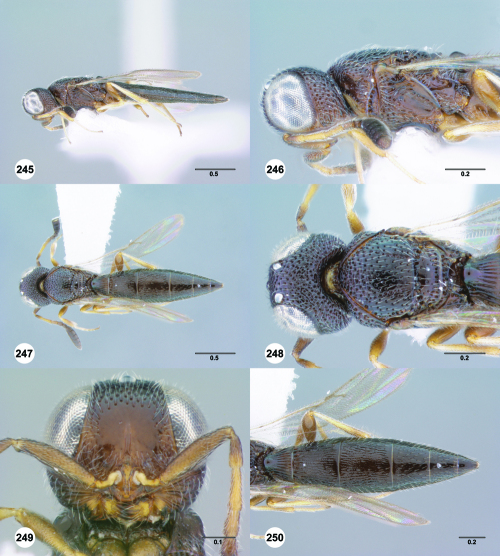
^108^Trichoteleia sphaerica sp. n., female holotype (CASENT 2135723). **245** Lateral habitus **246** Head and mesosoma, lateral view **247** Dorsal habitus **248** Head and mesosoma, dorsal view **249** Head, anterior view **250** Metasoma, dorsal view. Scale bars in millimeters.

Figures 245–250 Morphbank ^39^ 

#### Description.

##### Female body length:

2.61 mm (n=1). Color of head: reddish brown. Central keel of frons: present, extending onto interantennal process. Sculpture of medial frons in female: smooth. Number of mandibular teeth: three. Basal node on mandible: absent. Sculpture of frons below median ocellus: densely punctate throughout. Sculpture of posterior vertex: densely punctate. Occipital rim: comprised of small to miniscule cells. Sculpture of gena: punctate rugose. Basiconic sensillum on A7: absent.

##### Color of mesosoma in female:

reddish brown throughout. Sculpture along posterior pronotal sulcus: rugulose. Notaulus: smooth furrow incomplete, reaching suprahumeral sulcus as row of punctures. Sculpture of medial mesoscutum: moderately punctate in posterior half, becoming denser and transversely rugulose anteriorly. Sculpture of mesoscutellum: smooth medially, sparsely punctate laterally. Mesopleural carina: present only in anterior half. Sculpture along ventral half of prespecular sulcus: coarsely punctate. Sculpture of posterolateral mesepisternum: coarsely punctate. Setation of ventral metapleural area: absent. Setation of metapleural triangle: moderately dense. Sculpture of metapleural triangle: punctate rugose. Posterior margin of metapleuron below propodeal spiracle: straight to moderately convex. Color of legs: pale brown throughout.

##### Color of metasoma in female:

reddish brown. Posterior margin of transverse sulcus on T2: strongly convex. Sublateral tergal carina on T2: absent. Microsculpture on T2: present. Microsculpture on T3: present. Microsculpture on T4: present. Horn on T1 in female: present as a large, apically rounded protuberance. Macrosculpture of T2 in female: longitudinally strigose throughout. Macrosculpture of medial T3 in female: weakly longitudinally strigose. Macrosculpture of lateral T3 in female: longitudinally strigose. Macrosculpture of medial T4 in female: absent. Macrosculpture of lateral T4 in female: weakly rugulose. Punctation of T4 in female: moderately dense throughout. Macrosculpture of T5 in female: absent. Punctation of T5 in female: moderately dense throughout. Shape of T5 in female: width of posterior margin less than length. Microscupture on T6 in female: absent. Sculpture of T6 in female: smooth with sparse fine setigerous punctures throughout. Sculpture of S2: coarsely punctate throughout. Prominent longitudinal median carina on S2: absent.

##### Wings:

macropterous, apex or forewing extending beyond posterior margin of T3. Color of forewing in female: hyaline throughout. Color of hind wing: hyaline throughout. Density of setation in fore wing: uniform throughout. Density of setation in hind wing: uniform throughout. Length of R1: more than 1.5 times as long as r. M+Cu and RS+M in forewing: spectral.

#### Diagnosis.

Trichoteleia sphaerica resembles Trichoteleia bicolor in shape of the body but can be distinguished easily by its entirely hyaline forewings.

#### Etymology.

This species is named for the roughly spherical shape of its head. The epithet is considered an adjective.

#### Link to Distribution Map.

[http://hol.osu.edu/map-large.html?id=243954]

#### Material Examined.

Holotype, female: **MADAGASCAR:** Mahajanga Auto. Prov., 12.4km (337°) NNW Soalala, tropical dry forest, Baie de Baly National Park, 16°00'36"S, 45°15'54"E, 10m, 26.XI–30.XI.2002, yellow pan trap, Fisher, Griswold et al., CASENT 2135723 (deposited in CASC).

### Trichoteleia subtilis

Talamas & Masner sp. n.

urn:lsid:zoobank.org:act:AFC56042-5B79-42EF-B019-8410FACF5473

urn:lsid.biosci.ohio-state.edu:osuc_concepts:24340

Figures 5 251–256 Morphbank ^40^ 

#### Description.

##### Female body length:

2.22–3.32 mm (n=7). Male body length: 2.05–2.44 mm (n=20). Color of head: dark brown to black. Central keel of frons: present, bifurcating ventrally around interantennal process. Sculpture of medial frons in female: smooth. Sculpture of medial frons in male: smooth. Number of mandibular teeth: three. Basal node on mandible: absent. Sculpture of frons below median ocellus: dorsoventrally strigose throughout; densely punctate throughout. Sculpture of posterior vertex: moderately punctate; densely punctate; rugulose with faint concentric tendency. Occipital rim: comprised of small to miniscule cells. Sculpture of gena: dorsoventrally strigose. Basiconic sensillum on A7: absent.

##### Color of mesosoma in female:

dark brown to black. Color of mesosoma in male: dark brown to black. Sculpture along posterior pronotal sulcus: striate, striae well defined; striate, striae short and poorly defined. Notaulus: percurrent, reaching suprahumeral sulcus as a smooth furrow. Sculpture of medial mesoscutum: smooth and sparsely punctate posteriorly, densely punctate and transverely rugulose anteriorly; moderately punctate in posterior half, becoming denser anteriorly. Sculpture of mesoscutellum: smooth with sparse fine punctures throughout; smooth medially, sparsely punctate laterally. Postacetabular sulcus: present as a smooth furrow. Mesopleural carina: present. Sculpture along ventral half of prespecular sulcus: punctate rugose; weakly rugulose. Sculpture of posterolateral mesepisternum: smooth. Sculpture of ventral surface of mesepisternum: smooth. Setation of ventral metapleural area: absent. Setation of metapleural triangle: sparse. Sculpture of metapleural triangle: punctate rugose. Posterior margin of metapleuron below propodeal spiracle: with blunt kink near intersection with metapleural sulcus. Color of legs: yellow throughout; brown throughout.

##### Color of metasoma in female:

dark brown to black throughout. Color of metasoma in male: dark brown to black throughout. Posterior margin of transverse sulcus on T2: strongly convex. Sublateral tergal carina on T2: absent. Microsculpture on T2: absent. Microsculpture on T3: absent. Microsculpture on T4: absent. Horn on T1 in female: absent; present as a small bulge. Macrosculpture of T2 in female: longitudinally striate throughout. Macrosculpture of medial T3 in female: weakly longitudinally striate; absent. Macrosculpture of lateral T3 in female: weakly longitudinally striate; absent. Macrosculpture of medial T4 in female: absent. Macrosculpture of lateral T4 in female: absent. Punctation of T4 in female: moderately dense throughout; absent along midline, otherwise sparse; absent along midline, otherwise moderately dense. Macrosculpture of T5 in female: absent. Punctation of T5 in female: moderately dense throughout; sparse throughout. Shape of T5 in female: width of posterior margin greater than or equal to length. Microscupture on T6 in female: absent. Sculpture of T6 in female: smooth with fine setigerous punctures laterally. Macrosculpture of T2 in male: longitudinally striate throughout. Macrosculpture of medial T3 in male: absent; weakly longitudinally striate. Macrosculpture of lateral T3 in male: absent; weakly longitudinally striate. Macrosculpture of T4 in male: absent. Punctation of T4 in male: sparse throughout; absent along midline, otherwise sparse. Macrosculpture of T5 in male: absent. Punctation of T5 in male: moderately dense throughout; sparsely present throughout. Sculpture of S2: longitudinally striate throughout; longitudinally striate anteromedially, otherwise smooth. Prominent longitudinal median carina on S2: absent.

##### Wings:

macropterous, apex or forewing extending beyond posterior margin of T3. Color of forewing in female: slightly infuscate throughout. Color of forewing in male: slightly infuscate throughout. Color of hind wing: slightly infuscate throughout. Density of setation in fore wing: uniform throughout. Density of setation in hind wing: uniform throughout. Length of R1: more than 1.5 times as long as r. M+Cu and RS+M in forewing: nebulous.

**Figures 251–256.**
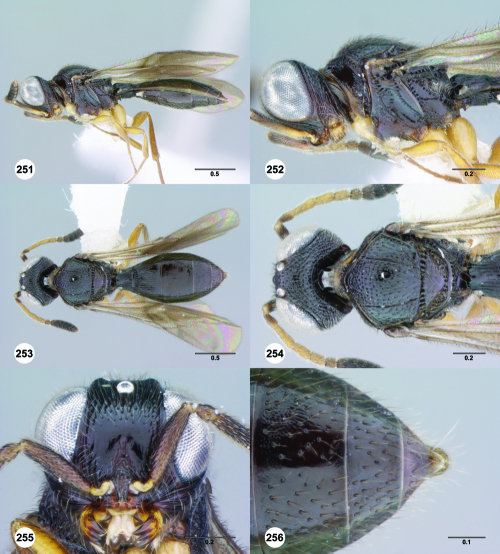
^109^Trichoteleia subtilis sp. n. **251** Lateral habitus, female (OSUC 237786) **252** Head and mesosoma, lateral view, female (OSUC 237786) **253** Dorsal habitus, female (OSUC 237786) **254** Head and mesosoma, dorsal view, female (OSUC 237786) **255** Head, anterior view, male (CASENT 2134187) **256** Metasoma, dorsal view, female (OSUC 237786). Scale bars in millimeters.

#### Diagnosis.

Trichoteleia subtilis is best identified by the fine striation of the gena, the absence of a basal node on the mandible, and the ventrally bifurcating central keel on the frons. T3–T4 are typically smooth, but some specimens bear faint striation on T3.

#### Etymology.

The Latin adjectival epithet *subtilis* refers to the fine striation of the gena.

#### Link to Distribution Map.

[http://hol.osu.edu/map-large.html?id=243406]

#### Material Examined.

Holotype, female: **MADAGASCAR:** Fianarantsoa Auto. Prov., Bell Vue Trail, tropical forest, Ranomafana National Park, 1000m, 12.XII–21.XII.1999, malaise trap, M. E. Irwin & E. I. Schlinger, OSUC 237786 (deposited in CASC). Paratypes: **MADAGASCAR:** 6 females, 33 males, CASENT 2043434, 2118411, 2134158, 2134173, 2134187, 2135761, 2135779–2135781, 2135798, 2135821, 2135830, 2135870, 2135874, 2135878, 2136121, 2136161, 2136183, 8106464 (CASC); OSUC 181052–181053, 181055–181059, 181064, 181067–181068 (CNCI); CASENT 2042843, 2043799, 2118410, 2135833, 2135871, 2136061, 2136181, 2136186 (OSUC); OSUC 241854, 241856 (USNM).

#### Comments.

This species varies in the definition of the striae on the vertex and below the median ocellus. Some specimens have well-defined striae, and others have primarily punctate sculpture with only a trace of striation.

### Trichoteleia tahotra

Talamas & Masner sp. n.

urn:lsid:zoobank.org:act:550240E6-EDA5-4C17-B954-D4F88DCC2816

urn:lsid.biosci.ohio-state.edu:osuc_concepts: 241271

Figures 16 19 25 36 257–262 Morphbank ^41^ 

#### Description.

##### Female body length:

2.96–3.48 mm (n=8). Male body length: 2.71–3.28 mm (n=16). Color of head: dark brown to black. Central keel of frons: present, extending onto interantennal process. Sculpture of medial frons in female: smooth. Sculpture of medial frons in male: smooth. Number of mandibular teeth: three. Basal node on mandible: present. Sculpture of frons below median ocellus: moderately punctate throughout; punctate throughout with a groove parallel to orbital carina; finely punctate throughout. Sculpture of posterior vertex: moderately punctate; punctate rugulose; finely punctate. Occipital rim: comprised of medium to large sized cells. Sculpture of gena: dorsoventrally strigose. Basiconic sensillum on A7: present.

##### Color of mesosoma in female:

variably orange to brown; reddish brown throughout. Color of mesosoma in male: pale brown throughout; dark brown to black. Sculpture along posterior pronotal sulcus: striate, striae short and poorly defined; rugulose. Notaulus: percurrent, reaching suprahumeral sulcus as a smooth furrow. Sculpture of medial mesoscutum: mostly smooth with few punctures, punctures denser anteriorly; smooth and sparsely punctate posteriorly, densely punctate and transverely rugulose anteriorly; longitudinally rugulose posteriorly, transversely rugulose anteriorly; moderately punctate in posterior half, becoming denser anteriorly; sparsely punctate, becoming denser anteriorly. Sculpture of mesoscutellum: smooth with sparse fine punctures throughout; smooth medially, coarsely punctate laterally; smooth medially, moderately punctate laterally. Postacetabular sulcus: present as a smooth furrow. Mesopleural carina: present only in anterior half. Sculpture along ventral half of prespecular sulcus: weakly rugulose. Sculpture of posterolateral mesepisternum: smooth. Sculpture of ventral surface of mesepisternum: smooth. Setation of ventral metapleural area: absent. Setation of metapleural triangle: sparse. Sculpture of metapleural triangle: rugulose; punctate rugose. Posterior margin of metapleuron below propodeal spiracle: straight to moderately convex; with blunt kink near intersection with metapleural sulcus. Color of legs: yellow throughout; pale brown throughout.

##### Color of metasoma in female:

dark brown to black throughout. Color of metasoma in male: pale to dark brown throughout; dark brown to black throughout. Posterior margin of transverse sulcus on T2: strongly convex. Sublateral tergal carina on T2: absent. Microsculpture on T2: absent; present. Microsculpture on T3: absent; present. Microsculpture on T4: absent; present. Horn on T1 in female: absent. Macrosculpture of T2 in female: longitudinally striate throughout. Macrosculpture of medial T3 in female: weakly longitudinally striate; longitudinally striate. Macrosculpture of lateral T3 in female: longitudinally striate. Macrosculpture of medial T4 in female: absent. Macrosculpture of lateral T4 in female: longitudinally strigose; longitudinally striate. Punctation of T4 in female: sparse laterally and along posterior margin, otherwise absent. Macrosculpture of T5 in female: absent; weakly rugulose laterally. Punctation of T5 in female: sparse throughout. Shape of T5 in female: width of posterior margin greater than or equal to length. Microscupture on T6 in female: absent. Sculpture of T6 in female: moderately punctate throughout. Macrosculpture of T2 in male: longitudinally striate throughout. Macrosculpture of medial T3 in male: absent; weakly longitudinally striate. Macrosculpture of lateral T3 in male: longitudinally striate. Macrosculpture of T4 in male: longitudinally strigose laterally; absent. Punctation of T4 in male: sparse laterally and along posterior margin, otherwise absent. Macrosculpture of T5 in male: absent; weakly rugulose laterally. Punctation of T5 in male: sparsely present throughout; absent in medial third, sparse laterally. Sculpture of S2: longitudinally striate throughout; longitudinally strigose throughout, punctate interstitially. Prominent longitudinal median carina on S2: absent.

##### Wings:

macropterous, apex or forewing extending beyond posterior margin of T3. Color of forewing in female: infuscate throughout; slightly infuscate throughout. Color of forewing in male: slightly infuscate throughout. Color of hind wing: slightly infuscate throughout. Density of setation in fore wing: uniform throughout. Density of setation in hind wing: uniform throughout. Length of R1: less than 1.5 times as long as rs. M+Cu and RS+M in forewing: nebulous.

**Figures 257–262.**
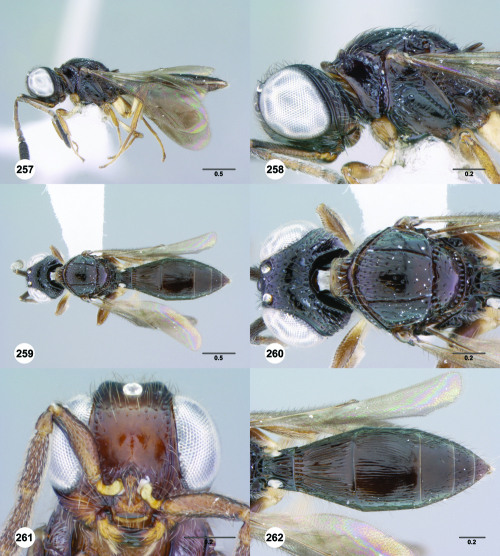
^110^Trichoteleia tahotra sp. n. **257** Lateral habitus, female (CASENT 2136058) **258** Head and mesosoma, lateral view, female (CASENT 2136058) **259** Dorsal habitus, female (CASENT 2136058) **260** Head and mesosoma, dorsal view, female (CASENT 2136058) **261** Head, anterior view, male (CASENT 2134151) **262** Metasoma, dorsal view, female (CASENT 2136058). Scale bars in millimeters.

#### Diagnosis.

Trichoteleia tahotra shares the presence of a posterior projection of the propodeum and shape of the lateral propodeal area with Trichoteleia oculea. In addition, R1 (postmarginal vein) in both species is approximately equal in length to r (stigmal vein). It differs from Trichoteleia oculea by the presence of a basiconic sensillum on A7 and in having short rugulae along the posterior margin of the lateral pronotum instead of well-defined striae (Fig. 16).

#### Etymology.

The Malagasy word for “fear”, *tahotra*, is given to this species as a noun in apposition in reference to its typically large metascutellar spines.

#### Link to Distribution Map.

[http://hol.osu.edu/map-large.html?id=241271]

#### Material Examined.

Holotype, female: **MADAGASCAR:** Antsiranana Auto. Prov., Marojejy National Park, 14°06'S 49°46.7'E, 750m, 22.X–19.XI.1996, malaise trap, E. Quinter, OSUC 181002 (deposited in CNCI). Paratypes: **MADAGASCAR:** 7 females, 17 males, CASENT 2043325, 2043340, 2043782, 2043787, 2134151, 2134186, 2134759, 2135842, 2136058, 2136422, 2136571, 2138067, OSUC 181000, OSUC 181009, OSUC 181010, OSUC 181065, OSUC 181066 (CASC); OSUC 181001, 262099 (CNCI); CASENT 2135802, 2135843, 2136187, 2136575, OSUC 215615 (OSUC). Other material: **MADAGASCAR:** 1 male, CASENT 2134733 (CASC).

#### Comments.

This species exhibits variation in color, ranging from pale brown to nearly black, in the length of the metascutellar spines, and in overall body size.

### Trichoteleia takariva

Talamas sp. n.

urn:lsid:zoobank.org:act:618B3053-16FC-4790-8E53-75206186C1A9

urn:lsid:biosci.ohio-state.edu:osuc_concepts: 244609

Figures 7 13 46 263–268 Morphbank ^42^ 

#### Description.

##### Male body length:

3.64 mm (n=1). Color of head: dark brown to black. Central keel of frons: present, extending onto interantennal process. Sculpture of medial frons in male: smooth. Number of mandibular teeth: three. Basal node on mandible: present. Sculpture of frons below median ocellus: punctate rugulose throughout. Sculpture of posterior vertex: moderately punctate, rugose posterior to eyes and posterior ocellus. Occipital rim: comprised of medium to large sized cells. Sculpture of gena: dorsoventrally strigose. Basiconic sensillum on A7: absent.

##### Color of mesosoma in male:

dark brown to black. Sculpture along posterior pronotal sulcus: striate, striae well defined. Notaulus: percurrent, reaching suprahumeral sulcus as a smooth furrow. Sculpture of medial mesoscutum: moderately punctate in posterior half, becoming denser anteriorly. Sculpture of mesoscutellum: smooth medially, moderately punctate laterally. Postacetabular sulcus: comprised of small cells. Mesopleural carina: present only in anterior half. Sculpture along ventral half of prespecular sulcus: punctate. Sculpture of posterolateral mesepisternum: smooth. Sculpture of ventral surface of mesepisternum: smooth. Setation of ventral metapleural area: absent. Setation of metapleural triangle: sparse. Sculpture of metapleural triangle: punctate rugose. Posterior margin of metapleuron below propodeal spiracle: with blunt kink near intersection with metapleural sulcus. Color of legs: coxae and trochanters yellow, otherwise brown.

##### Color of metasoma in male:

dark brown to black throughout. Posterior margin of transverse sulcus on T2: strongly convex. Sublateral tergal carina on T2: absent. Microsculpture on T2: absent. Microsculpture on T3: absent. Microsculpture on T4: absent. Macrosculpture of T2 in male: longitudinally striate throughout. Macrosculpture of medial T3 in male: longitudinally striate. Macrosculpture of lateral T3 in male: longitudinally striate. Macrosculpture of T4 in male: longitudinally strigose laterally. Punctation of T4 in male: absent along midline, otherwise moderately dense throughout. Macrosculpture of T5 in male: absent. Punctation of T5 in male: absent along midline, otherwise moderately dense throughout. Sculpture of S2: longitudinally striate anteriorly, smooth posteriorly. Prominent longitudinal median carina on S2: absent.

##### Wings:

Wings: macropterous, apex or forewing extending beyond posterior margin of T3. Color of forewing in male: infuscate throughout. Color of hind wing: slightly infuscate throughout. Density of setation in fore wing: uniform throughout. Density of setation in hind wing: uniform throughout. Length of R1: more than 1.5 times as long as r. M+Cu and RS+M in forewing: nebulous.

**Figures 263–268.**
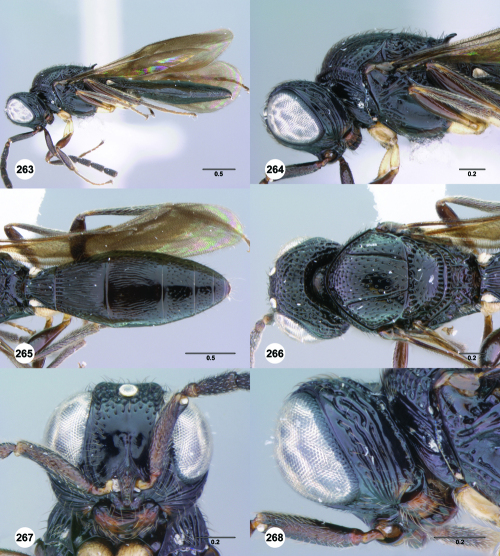
^111^Trichoteleia takariva sp. n., male holotype (CASENT 2043217). **263** Lateral habitus **264** Head and mesosoma, lateral view **265** Metasoma, dorsal view. **266** Head and mesosoma, dorsal view **267** Head, anterior view **268** Gena, posterolateral view. Scale bars in millimeters.

#### Diagnosis.

Trichoteleia takariva is most similar to males of Trichoteleia orona. Trichoteleia takariva lacks a dorsal flange on the interantennal process (Fig. 264) and has moderately dense punctation on T5 (Fig. 265), whereas in Trichoteleia orona the flange is present (Fig. 187) and the punctation of T5 is dense (Fig. 40).

#### Etymology.

The epithet *takariva* means “nightfall” in Malagasy. It is applied to this species because of its black color and dusky wings. The epithet is a noun in apposition.

#### Link to Distribution Map.

[http://hol.osu.edu/map-large.html?id=244609]

#### Material Examined.

Holotype, male: **MADAGASCAR:** Toliara Auto. Prov., nr. Bellevue, parcel II, spiny forest, MA-02-14B-05, Bezaha Mahafaly Special Reserve, 23°41.39'S 44°34.53'E, 180m, 4.XII–11.XII.2001, malaise trap, R. Harin’Hala, CASENT 2043217 (deposited in CASC). Paratype: **MADAGASCAR:** 1 male, CASENT 2137241 (CASC).

### Trichoteleia tezitra

Talamas sp. n.

urn:lsid:zoobank.org:act:6D355E62-584A-46F2-B9A9-DBF221D0E1BB

urn:lsid.biosci.ohio-state.edu:osuc_concepts:241273

**Figures 269–274.**
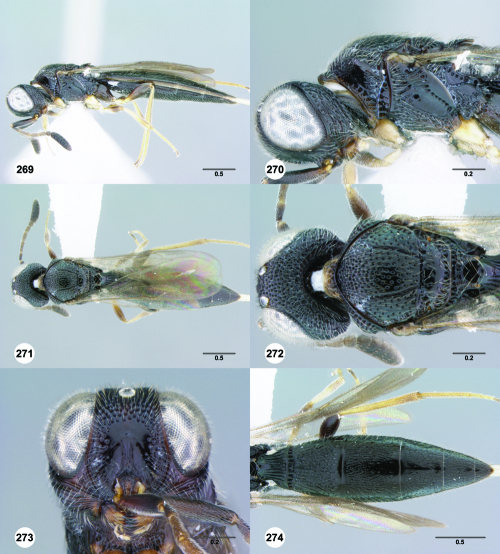
^112^Trichoteleia tezitra sp. n. **269** Lateral habitus, female (CASENT 2133132) **270** Head and mesosoma, lateral view, female (CASENT 2133132) **271** Dorsal habitus, female (CASENT 2133132) **272** Head and mesosoma, dorsal view, female (CASENT 2133132) **273** Head, anterior view, female (CASENT 2135929) **274** Metasoma, dorsal view, female (CASENT 2133132). Scale bars in millimeters.

Figures 269–274 Morphbank ^43^ 

#### Description.

##### Female body length:

3.67–4.25 mm (n=3). Male body length: 3.56 mm (n=1). Color of head: dark brown to black. Central keel of frons: present, bifurcating ventrally around interantennal process. Sculpture of medial frons in female: smooth. Sculpture of medial frons in male: smooth. Number of mandibular teeth: three. Basal node on mandible: absent. Sculpture of frons below median ocellus: densely punctate throughout. Sculpture of posterior vertex: densely punctate. Occipital rim: comprised of medium to large sized cells. Sculpture of gena: dorsoventrally strigose. Basiconic sensillum on A7: absent.

##### Color of mesosoma in female:

dark brown to black. Color of mesosoma in male: dark brown to black. Sculpture along posterior pronotal sulcus: striate, striae well defined; rugulose. Notaulus: smooth furrow incomplete, reaching suprahumeral sulcus as row of punctures. Sculpture of medial mesoscutum: densely punctate posteriorly, becoming denser and transversely rugulose anteriorly. Sculpture of mesoscutellum: smooth medially, moderately punctate laterally. Postacetabular sulcus: comprised of small cells. Mesopleural carina: present. Sculpture along ventral half of prespecular sulcus: coarsely punctate. Sculpture of posterolateral mesepisternum: coarsely punctate. Sculpture of ventral surface of mesepisternum: finely punctate; moderately punctate. Setation of ventral metapleural area: absent. Setation of metapleural triangle: sparse. Sculpture of metapleural triangle: punctate rugose. Posterior margin of metapleuron below propodeal spiracle: straight to moderately convex; with blunt kink near intersection with metapleural sulcus. Color of legs: femora and metacoxa brown, otherwise yellow.

##### Color of metasoma in female:

dark brown to black throughout. Color of metasoma in male: dark brown to black throughout. Posterior margin of transverse sulcus on T2: strongly convex. Sublateral tergal carina on T2: absent. Microsculpture on T2: present. Microsculpture on T3: present. Microsculpture on T4: absent. Horn on T1 in female: present as a large, apically rounded protuberance. Macrosculpture of T2 in female: longitudinally strigose throughout. Macrosculpture of medial T3 in female: absent; longitudinally strigose; rugulose. Macrosculpture of lateral T3 in female: longitudinally strigose. Macrosculpture of medial T4 in female: absent. Macrosculpture of lateral T4 in female: absent; weakly rugulose. Punctation of T4 in female: sparse in medial third, dense laterally; absent along midline, otherwise moderately dense. Macrosculpture of T5 in female: absent; weakly rugulose laterally. Punctation of T5 in female: dense throughout; sparse along midline, otherwise dense; absent along midline, otherwise moderately dense. Shape of T5 in female: width of posterior margin less than length. Microscupture on T6 in female: absent. Sculpture of T6 in female: densely and finely punctate. Macrosculpture of T2 in male: longitudinally strigose. Macrosculpture of medial T3 in male: longitudinally strigose. Macrosculpture of lateral T3 in male: longitudinally strigose. Macrosculpture of T4 in male: absent. Punctation of T4 in male: sparse in medial third, dense laterally. Macrosculpture of T5 in male: absent. Punctation of T5 in male: sparse along midline, otherwise dense throughout. Sculpture of S2: coarsely punctate throughout. Prominent longitudinal median carina on S2: absent.

##### Wings:

macropterous, apex or forewing extending beyond posterior margin of T3. Color of forewing in female: slightly infuscate throughout. Color of forewing in male: slightly infuscate throughout. Color of hind wing: slightly infuscate throughout. Density of setation in fore wing: uniform throughout. Density of setation in hind wing: uniform throughout. Length of R1: more than 1.5 times as long as r. M+Cu and RS+M in forewing: spectral.

#### Diagnosis.

The body shape, color and size of Trichoteleia tezitra is similar to Trichoteleia bidentata, Trichoteleia funesta and, to a lesser extent, Trichoteleia quazii. S2 in Trichoteleia quazii has a longitudinal median carina (as in Fig. 45), whereas S2 in Trichoteleia tezitra is uniformly punctate with no carina. Trichoteleia tezitra shares with Trichoteleia bidentata spectral Rs+M and M+Cu veins, and differs in having three, versus two, mandibular teeth.

#### Etymology.

This species is given the name *tezitra*, meaning “angry” in Malagasy, for the appearance of its head. The epithet is to be considered as a noun in apposition.

#### Link to Distribution Map.

[http://hol.osu.edu/map-large.html?id=241273]

#### Material Examined.

Holotype, female: **MADAGASCAR:** Toliara Auto. Prov., 19.8km (84°) E Sakaraha, leaf mold / rotten wood / tropical dry forest, BLF7510, Zombitse-Vohibasia National Park, 22°50'36"S, 44°42'36"E, 770m, 5.II–9.II.2003, sifted litter, Fisher, Griswold et al., CASENT 2136542 (deposited in CASC). Paratypes: **MADAGASCAR:** 3 females, 1 male, CASENT 2133132, 2133135, OSUC 334221 (CASC); CASENT 2135929 (OSUC).

### Trichoteleia tigris

Talamas sp. n.

urn:lsid:zoobank.org:act:DD0DBED7-3C5B-4A99-BDD6-DF3531135DC5

urn:lsid.biosci.ohio-state.edu:osuc_concepts:241286

Figures 22 28 53–54 275–280 Morphbank ^44^ 

#### Description.

##### Female body length:

3.26 mm (n=1). Male body length: 2.65–3.55 mm (n=12). Color of head: pale brown throughout; reddish brown. Central keel of frons: present, extending onto interantennal process. Sculpture of medial frons in female: punctate rugulose. Sculpture of medial frons in male: punctate. Number of mandibular teeth: three. Basal node on mandible: absent. Sculpture of frons below median ocellus: punctate rugulose throughout; densely punctate throughout. Sculpture of posterior vertex: punctate rugose. Occipital rim: comprised of medium to large sized cells. Sculpture of gena: punctate rugose; coarsely strigose; dorsoventrally strigose. Basiconic sensillum on A7: absent.

##### Color of mesosoma in female:

variably orange to brown. Color of mesosoma in male: pale brown throughout. Sculpture along posterior pronotal sulcus: striate, striae well defined; striate, striae short and poorly defined. Notaulus: percurrent, reaching suprahumeral sulcus as a smooth furrow. Sculpture of medial mesoscutum: densely punctate throughout; moderately punctate in posterior half, becoming denser anteriorly. Sculpture of mesoscutellum: smooth with few very fine punctures laterally. Postacetabular sulcus: comprised of small cells. Mesopleural carina: present. Sculpture along ventral half of prespecular sulcus: longitudinally striate; coarsely punctate. Sculpture of posterolateral mesepisternum: punctate. Sculpture of ventral surface of mesepisternum: smooth. Setation of ventral metapleural area: absent. Setation of metapleural triangle: dense; moderately dense. Sculpture of metapleural triangle: punctate rugose. Posterior margin of metapleuron below propodeal spiracle: with blunt kink near intersection with metapleural sulcus. Color of legs: coxae and trochanters yellow, otherwise pale brown, hindlegs the darkest.

##### Color of metasoma in female:

T1–T2, T4 brown medially, otherwise orange throughout. Color of metasoma in male: pale to dark brown throughout. Posterior margin of transverse sulcus on T2: strongly convex. Sublateral tergal carina on T2: absent. Microsculpture on T2: present. Microsculpture on T3: present. Microsculpture on T4: absent. Horn on T1 in female: absent. Macrosculpture of T2 in female: longitudinally striate throughout. Macrosculpture of medial T3 in female: longitudinally striate. Macrosculpture of lateral T3 in female: longitudinally striate. Macrosculpture of medial T4 in female: absent. Macrosculpture of lateral T4 in female: absent. Punctation of T4 in female: absent along midline, otherwise moderately dense. Macrosculpture of T5 in female: absent. Punctation of T5 in female: absent along midline, otherwise moderately dense. Shape of T5 in female: width of posterior margin greater than or equal to length. Microscupture on T6 in female: absent. Sculpture of T6 in female: smooth with fine setigerous punctures along lateral margin. Macrosculpture of T2 in male: longitudinally striate throughout; weakly longitudinally striate throughout. Macrosculpture of medial T3 in male: longitudinally striate. Macrosculpture of lateral T3 in male: longitudinally striate. Macrosculpture of T4 in male: absent. Punctation of T4 in male: absent along midline, otherwise sparse; absent along midline, otherwise moderately dense throughout. Macrosculpture of T5 in male: absent. Punctation of T5 in male: absent along midline, otherwise moderately dense throughout; absent along midline, otherwise sparse. Sculpture of S2: longitudinally strigose throughout, punctate interstitially. Prominent longitudinal median carina on S2: absent.

##### Wings:

macropterous, apex or forewing extending beyond posterior margin of T3. Color of forewing in female: hyaline with transverse infuscate bands medially and subapically. Color of forewing in male: slightly infuscate throughout. Color of hind wing: slightly infuscate throughout. Density of setation in fore wing: reduced posterior to Sc+R in basal half. Density of setation in hind wing: reduced posterior to Sc+R. Length of R1: more than 1.5 times as long as r. M+Cu and RS+M in forewing: nebulous.

**Figures 275–280.**
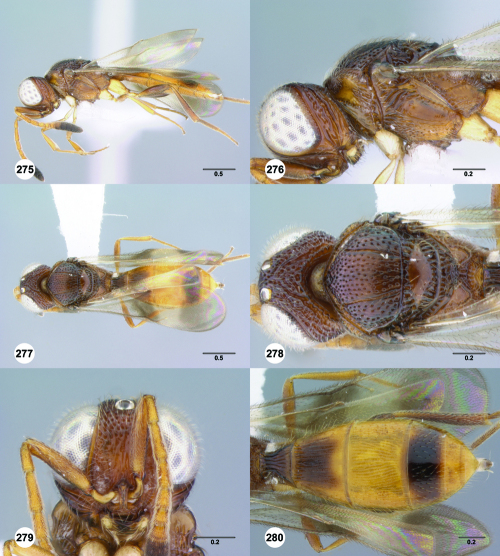
^113^Trichoteleia tigris sp. n., female holotype (CASENT 2043960). **275** Lateral habitus **276** Head and mesosoma, lateral view **277** Dorsal habitus **278** Head and mesosoma, dorsal view **279** Head, anterior view **280** Metasoma, dorsal view. Scale bars in millimeters.

#### Diagnosis.

Trichoteleia tigris is a rather disparate species with unclear affinities. The smooth area present above the interantennal process in other species of Trichoteleia is entirely rugulose in the female specimen and mostly rugulose in the male specimens. The coarse punctation and rugosity of the head (Fig. 278), the short, blunt metascutellar points (Fig. 22), banded wings (females only) (Fig. 277), and the large, mostly smooth lateral propodeal area (Fig. 28) serve well to the identify this species.

#### Etymology.

The epithet *tigris* refers to the banding pattern of the metasoma in females of this species. The name is used as a noun in apposition.

#### Link to Distribution Map.

[http://hol.osu.edu/map-large.html?id=241286]

#### Material Examined.

Holotype, female: **MADAGASCAR:** Fianarantsoa Auto. Prov., radio tower, forest edge / mixed tropical forest, MA-02-09B-53, Ranomafana National Park, 21°15.05'S 47°24.43'E, 1130m, 18.II–27.II.2003, malaise trap, R. Harin’Hala, CASENT 2043960 (deposited in CASC). Paratypes: **MADAGASCAR:** 14 males, CASENT 2135854, 2135960, 2135963, 2135965, 2135969–2135970, 2136013, 2136185, 2137692 (CASC); CASENT 2135972 (CNCI); CASENT 2135820, 2135822, 2135966, OSUC 254610 (OSUC).

### Trichoteleia tonsa

Talamas & Masner sp. n.

urn:lsid:zoobank.org:act:C7C17BE8-7410-4767-BB68-E540E35D6E3C

urn:lsid.biosci.ohio-state.edu:osuc_concepts:241292

**Figures 281–286.**
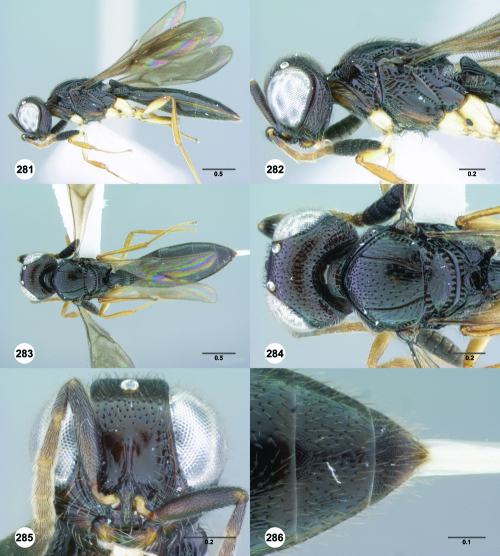
^114^Trichoteleia tonsa sp. n. **281** Lateral habitus, female (CASENT 2042828) **282** Head and mesosoma, lateral view, female (CASENT 2042828) **283** Dorsal habitus, female holotype (CASENT 2042832). **284** Head and mesosoma, dorsal view, female holotype (CASENT 2042832) **285** Head, anterior view, female holotype (CASENT 2042832) **286** T5**–**T6, dorsal view, female holotype (CASENT 2042832). Scale bars in millimeters.

Figures 281–286 Morphbank ^45^ 

#### Description.

##### Female body length:

3.26–3.72 mm (n=12). Male body length: 3.36 mm (n=1). Color of head: dark brown to black. Central keel of frons: present, extending onto interantennal process. Sculpture of medial frons in female: smooth. Sculpture of medial frons in male: smooth. Number of mandibular teeth: three. Basal node on mandible: absent. Sculpture of frons below median ocellus: finely punctate throughout. Sculpture of posterior vertex: moderately punctate; finely punctate. Occipital rim: comprised of small to miniscule cells. Sculpture of gena: dorsoventrally strigose. Basiconic sensillum on A7: absent.

##### Color of mesosoma in female:

dark brown to black. Color of mesosoma in male: dark brown to black. Sculpture along posterior pronotal sulcus: striate, striae well defined. Notaulus: percurrent, reaching suprahumeral sulcus as a smooth furrow. Sculpture of medial mesoscutum: moderately punctate in posterior half, becoming denser anteriorly. Sculpture of mesoscutellum: smooth medially, sparsely punctate laterally. Postacetabular sulcus: comprised of small cells. Mesopleural carina: present. Sculpture along ventral half of prespecular sulcus: punctate. Sculpture of posterolateral mesepisternum: smooth. Sculpture of ventral surface of mesepisternum: smooth. Setation of ventral metapleural area: absent. Setation of metapleural triangle: absent; sparse. Sculpture of metapleural triangle: smooth. Posterior margin of metapleuron below propodeal spiracle: straight to moderately convex. Color of legs: yellow throughout; coxae, trochanters and femora yellow, otherwise pale to dark brown.

##### Color of metasoma in female:

dark brown to black throughout. Color of metasoma in male: dark brown to black throughout. Posterior margin of transverse sulcus on T2: strongly convex. Sublateral tergal carina on T2: absent. Microsculpture on T2: present. Microsculpture on T3: present. Microsculpture on T4: absent. Horn on T1 in female: present as a large, apically rounded protuberance. Macrosculpture of T2 in female: longitudinally striate throughout. Macrosculpture of medial T3 in female: longitudinally striate. Macrosculpture of lateral T3 in female: longitudinally striate. Macrosculpture of medial T4 in female: absent. Macrosculpture of lateral T4 in female: longitudinally strigose. Punctation of T4 in female: sparse in medial third, moderately dense laterally; absent along midline, otherwise moderately dense. Macrosculpture of T5 in female: absent; weakly rugulose laterally. Punctation of T5 in female: moderately dense throughout. Shape of T5 in female: width of posterior margin greater than or equal to length. Microscupture on T6 in female: present throughout. Sculpture of T6 in female: finely punctate throughout. Macrosculpture of T2 in male: longitudinally striate throughout. Macrosculpture of medial T3 in male: longitudinally striate. Macrosculpture of lateral T3 in male: longitudinally striate. Macrosculpture of T4 in male: longitudinally strigose laterally. Punctation of T4 in male: sparse throughout. Macrosculpture of T5 in male: weakly rugulose laterally. Punctation of T5 in male: sparsely present throughout. Sculpture of S2: longitudinally striate anteriorly, smooth posteriorly. Prominent longitudinal median carina on S2: absent.

##### Wings:

macropterous, apex or forewing extending beyond posterior margin of T3. Color of forewing in female: infuscate throughout; slightly infuscate throughout. Color of forewing in male: slightly infuscate throughout. Color of hind wing: infuscate throughout; slightly infuscate throughout. Density of setation in fore wing: uniform throughout. Density of setation in hind wing: uniform throughout. Length of R1: more than 1.5 times as long as r. M+Cu and RS+M in forewing: nebulous.

#### Diagnosis.

Trichoteleia tonsa belongs to the cluster of species, also containing Trichoteleia levii and Trichoteleia prima, in which the short striae along the pronotal shoulder and those of the lateral pronotum form a continuous band. Females of Trichoteleia tonsa are separated by the presence of dense microsculpture throughout T6 (Fig. 286), and males by the smooth metapleural triangle (Fig. 282) and posteriorly rounded axillular carina (as in Fig. 11).

#### Etymology.

The epithet *tons*a, meaning “paddle” in Latin, refers to the shape of the forewing in this species. It is used as a noun in apposition.

#### Link to Distribution Map.

[http://hol.osu.edu/map-large.html?id=241292]

#### Material Examined.

Holotype, female: **MADAGASCAR:** Toamasina Auto. Prov., 18km (21°) NNE Ambinanitelo, rainforest, BLF8004, Mount Anjanaharibe, 15°11'18"S, 49°36'54"E, 450m, 8.III–12.III.2003, yellow pan trap, Fisher, Griswold et al., CASENT 2042832 (deposited in CASC). Paratypes: **MADAGASCAR:** 11 females, 1 male, CASENT 2042826, 2042828, 2042864, 2135942, 2135947, 2135950, 2135954, 2135956 (CASC); CASENT 2135951 (CNCI); CASENT 2135941, 2135943, 2135948 (OSUC).

### Trichoteleia warreni

Talamas & Masner sp. n.

urn:lsid:zoobank.org:act:4D495022-4B36-4E46-9659-6ED006733AF2

urn:lsid.biosci.ohio-state.edu:osuc_concepts:243938

**Figures 287–292.**
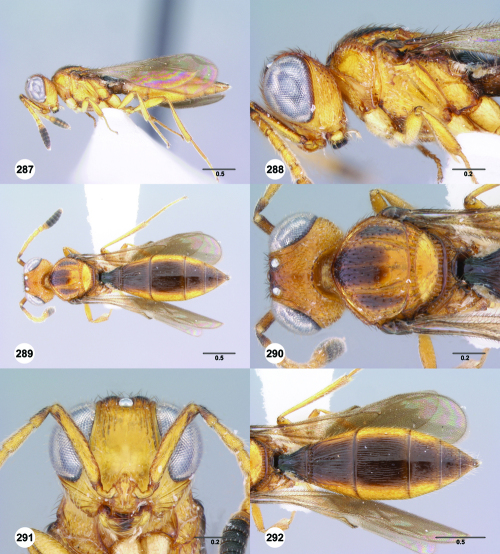
^115^Trichoteleia warreni sp. n., female holotype (OSUC 181060). **287** Lateral habitus **288** Head and mesosoma, lateral view **289** Dorsal habitus **290** Head and mesosoma, dorsal view **291** Head, anterior view **292** Metasoma, dorsal view. Scale bars in millimeters.

Figures 287–292 Morphbank ^46^ 

#### Description.

##### Female body length:

3.29 mm (n=1). Color of head: yellow throughout. Central keel of frons: present, extending onto interantennal process. Sculpture of medial frons in female: smooth. Number of mandibular teeth: three. Basal node on mandible: present. Sculpture of frons below median ocellus: dorsoventrally strigose throughout. Sculpture of posterior vertex: moderately punctate, rugose posterior to eyes and posterior ocellus. Occipital rim: comprised of medium to large sized cells. Sculpture of gena: dorsoventrally strigose. Basiconic sensillum on A7: absent.

##### Color of mesosoma in female:

variably yellow to brown. Sculpture along posterior pronotal sulcus: striate, striae short and poorly defined. Notaulus: percurrent, reaching suprahumeral sulcus as a smooth furrow. Sculpture of medial mesoscutum: moderately punctate in posterior half, becoming denser anteriorly. Sculpture of mesoscutellum: smooth medially, sparsely punctate laterally. Postacetabular sulcus: comprised of small cells. Mesopleural carina: present. Sculpture along ventral half of prespecular sulcus: punctate rugose. Sculpture of posterolateral mesepisternum: smooth. Sculpture of ventral surface of mesepisternum: smooth. Setation of ventral metapleural area: absent. Setation of metapleural triangle: sparse. Sculpture of metapleural triangle: punctate rugose. Posterior margin of metapleuron below propodeal spiracle: with blunt kink near intersection with metapleural sulcus. Color of legs: yellow throughout.

##### Color of metasoma in female:

T1, S1, medial T2–T6 brown, otherwise yellow. Posterior margin of transverse sulcus on T2: strongly convex. Sublateral tergal carina on T2: absent. Microsculpture on T2: present. Microsculpture on T3: present. Microsculpture on T4: absent. Horn on T1 in female: present as a large, apically rounded protuberance. Macrosculpture of T2 in female: longitudinally striate throughout. Macrosculpture of medial T3 in female: longitudinally striate. Macrosculpture of lateral T3 in female: longitudinally striate. Macrosculpture of medial T4 in female: absent. Macrosculpture of lateral T4 in female: longitudinally strigose. Punctation of T4 in female: absent along midline, otherwise moderately dense. Macrosculpture of T5 in female: punctate rugulose laterally. Punctation of T5 in female: absent along midline, otherwise moderately dense. Shape of T5 in female: width of posterior margin greater than or equal to length. Microscupture on T6 in female: absent. Sculpture of T6 in female: punctate rugulose. Sculpture of S2: longitudinally strigose medially, otherwise smooth with sparse fine punctation. Prominent longitudinal median carina on S2: absent.

##### Wings:

macropterous, apex or forewing extending beyond posterior margin of T3. Color of forewing in female: slightly infuscate throughout. Color of hind wing: slightly infuscate throughout. Density of setation in fore wing: uniform throughout. Density of setation in hind wing: uniform throughout. Length of R1: more than 1.5 times as long as r. M+Cu and RS+M in forewing: nebulous.

#### Diagnosis.

Trichoteleia warreni is similar to Trichoteleia rugifrons and Trichoteleia solocis with which it shares striae or rugulae on lateral T4–T5 that span the length of the tergites. The frons below the median ocellus in Trichoteleia warreni is evenly dorsoventrally strigose (Fig. 291), whereas in Trichoteleia rugifrons and Trichoteleia solocis, the dorsoventral rugae attenuate or are absent medially (Figs 243, 237).

#### Etymology.

Trichoteleia warreni is named for Warren Steiner of the US National Museum of Natural History for collection of the holotype specimen of this species, and for the contribution of many other specimens treated in this revision.

#### Link to Distribution Map.

[http://hol.osu.edu/map-large.html?id=243938]

#### Material Examined.

Holotype, female: **MADAGASCAR:** Fianarantsoa Auto. Prov., 7km W Ranomafana, 1100m, 22.X–31.X.1988, malaise trap, W. Steiner, OSUC 181060 (deposited in USNM). Paratype: **MADAGASCAR:** 1 female, OSUC 254607 (CASC).

### Trichoteleia xantrox

Talamas sp. n.

urn:lsid:zoobank.org:act:DD00884E-5D03-42A0-9E34-412EB772070B

urn:lsid.biosci.ohio-state.edu:osuc_concepts:241274

**Figures 293–298.**
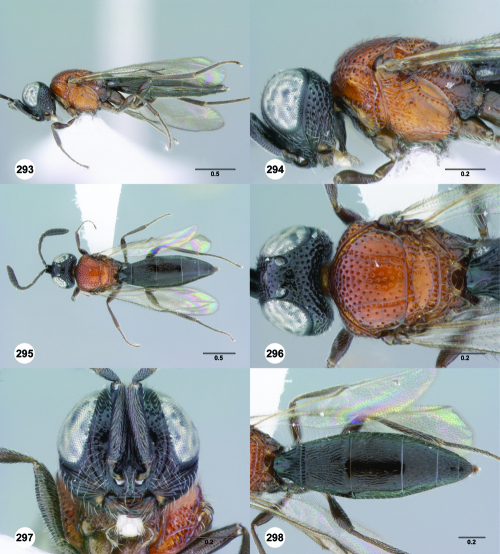
Trichoteleia xantrox sp. n., female holotype (CASENT 2043317) **293** Lateral habitus **294** Head and mesosoma, lateral view **295** Dorsal habitus **296** Head and mesosoma, dorsal view **297** Head, anterior view **298** Metasoma, dorsal view. Scale bars in millimeters.

Figures 293–298 Morphbank ^47^ 

#### Description.

##### Female body length:

2.65 mm (n=1). Color of head: dark brown to black. Central keel of frons: present, extending onto interantennal process. Sculpture of medial frons in female: smooth. Number of mandibular teeth: three. Basal node on mandible: absent. Sculpture of frons below median ocellus: punctate laterally, mostly smooth medially. Sculpture of posterior vertex: moderately punctate. Occipital rim: comprised of medium to large sized cells. Sculpture of gena: punctate rugose. Basiconic sensillum on A7: absent.

##### Color of mesosoma in female:

posterior metapleuron brown, otherwise orange. Sculpture along posterior pronotal sulcus: rugulose. Notaulus: smooth furrow incomplete, reaching suprahumeral sulcus as row of punctures. Sculpture of medial mesoscutum: moderately punctate in posterior half, becoming denser anteriorly. Sculpture of mesoscutellum: smooth medially, sparsely punctate laterally. Postacetabular sulcus: comprised of small cells. Mesopleural carina: present. Sculpture along ventral half of prespecular sulcus: punctate. Sculpture of posterolateral mesepisternum: smooth except for 1 or 2 rows of fine punctures parallel to mesopleural carina. Sculpture of ventral surface of mesepisternum: smooth. Setation of ventral metapleural area: absent. Setation of metapleural triangle: sparse. Sculpture of metapleural triangle: punctate rugose. Posterior margin of metapleuron below propodeal spiracle: straight to moderately convex. Color of legs: brown throughout.

##### Color of metasoma in female:

dark brown to black throughout. Posterior margin of transverse sulcus on T2: straight. Sublateral tergal carina on T2: absent. Microsculpture on T2: present. Microsculpture on T3: present. Microsculpture on T4: absent. Horn on T1 in female: present as a large, apically rounded protuberance. Macrosculpture of T2 in female: longitudinally strigose throughout. Macrosculpture of medial T3 in female: absent. Macrosculpture of lateral T3 in female: weakly longitudinally strigose. Macrosculpture of medial T4 in female: absent. Macrosculpture of lateral T4 in female: absent. Punctation of T4 in female: absent along midline, otherwise sparse. Macrosculpture of T5 in female: absent. Punctation of T5 in female: sparse throughout. Shape of T5 in female: width of posterior margin greater than or equal to length. Microscupture on T6 in female: absent. Sculpture of T6 in female: finely punctate throughout. Sculpture of S2: coarsely punctate throughout. Prominent longitudinal median carina on S2: absent.

##### Wings:

macropterous, apex or forewing extending beyond posterior margin of T3. Color of forewing in female: hyaline throughout. Color of hind wing: hyaline throughout. Density of setation in fore wing: uniform throughout. Density of setation in hind wing: uniform throughout. Length of R1: more than 1.5 times as long as r. M+Cu and RS+M in forewing: spectral.

#### Diagnosis.

The most distinguishing character of Trichoteleia xantrox is the transverse furrow on T2, which has the anterior and posterior margins parallel across the entire tergite (Fig. 298). Trichoteleia jiro and Trichoteleia afo have the margins of the furrow parallel medially but converging laterally. Trichoteleia xantrox may also be separated from these two species by the deep furrow laterad of the orbital carina that extends to the vertex along the inner margin of the eye (Fig. 297).

#### Etymology.

The epithet *xantrox* is a meaningless, euphonic combination of letters. It is to be treated as a noun in apposition.

#### Link to Distribution Map.

[http://hol.osu.edu/map-large.html?id=241274]

#### Material Examined.

Holotype, female: **MADAGASCAR:** Antsiranana Auto. Prov., MA-01-01D-08, Montagne d’Ambre National Park, 12°31'13"S, 49°10'45"E, 1125m, 21.IV–26.IV.2001, malaise trap, R. Harin’Hala, CASENT 2043317 (deposited in CASC). Paratypes: **MADAGASCAR:** 2 females, CASENT 2008587, 2134767 (CASC).

### Trichoteleia zuparkoi

Talamas & Masner sp. n.

urn:lsid:zoobank.org:act:51C372B1-014B-48A3-A271-73B24DD0B5F2

urn:lsid.biosci.ohio-state.edu:osuc_concepts:243937

**Figures 299–304.**
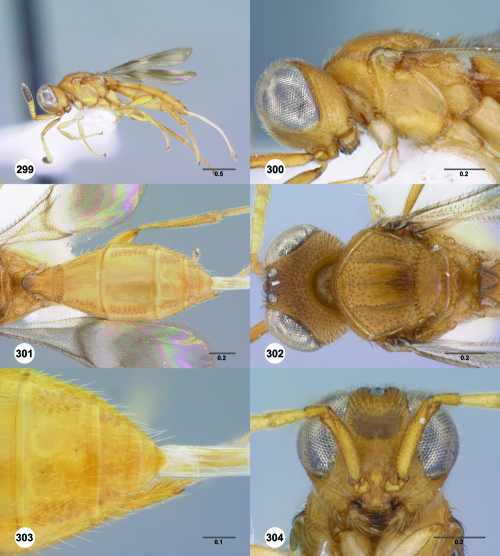
^117^Trichoteleia zuparkoi sp. n., female holotype (OSUC 181161). **299** Lateral habitus **300** Head and mesosoma, lateral view **301** Metasoma, dorsal view **302** Head and mesosoma, dorsal view **303** T4–T6, dorsal view **304** Head, anterior view. Scale bars in millimeters.

Figures 299–304 Morphbank ^48^ 

#### Description.

##### Female body length:

2.55 mm (n=1). Color of head: yellow throughout. Central keel of frons: present, extending onto interantennal process. Sculpture of medial frons in female: smooth. Number of mandibular teeth: three. Basal node on mandible: present. Sculpture of frons below median ocellus: punctate rugulose throughout. Sculpture of posterior vertex: moderately punctate, rugulose posterior to eyes and posterior ocellus. Occipital rim: comprised of medium to large sized cells. Sculpture of gena: punctate rugulose. Basiconic sensillum on A7: absent.

##### Color of mesosoma in female:

yellow throughout. Sculpture along posterior pronotal sulcus: striate, striae well defined. Notaulus: percurrent, reaching suprahumeral sulcus as a smooth furrow. Sculpture of medial mesoscutum: moderately punctate in posterior half, becoming denser and transversely rugulose anteriorly. Sculpture of mesoscutellum: smooth medially, moderately punctate laterally. Postacetabular sulcus: present as a smooth furrow. Mesopleural carina: present. Sculpture along ventral half of prespecular sulcus: punctate rugose. Sculpture of posterolateral mesepisternum: smooth. Sculpture of ventral surface of mesepisternum: smooth. Setation of ventral metapleural area: absent. Setation of metapleural triangle: sparse. Sculpture of metapleural triangle: rugulose. Posterior margin of metapleuron below propodeal spiracle: straight to moderately convex. Color of legs: yellow throughout.

##### Color of metasoma in female:

yellow. Posterior margin of transverse sulcus on T2: strongly convex. Sublateral tergal carina on T2: absent. Microsculpture on T2: absent. Microsculpture on T3: absent. Microsculpture on T4: absent. Horn on T1 in female: present as a large, apically rounded protuberance. Macrosculpture of T2 in female: longitudinally striate throughout. Macrosculpture of medial T3 in female: absent. Macrosculpture of lateral T3 in female: longitudinally striate. Macrosculpture of medial T4 in female: absent. Macrosculpture of lateral T4 in female: obliquely strigose. Punctation of T4 in female: sparse throughout. Macrosculpture of T5 in female: weakly rugulose laterally. Punctation of T5 in female: sparse throughout. Shape of T5 in female: width of posterior margin greater than or equal to length. Microscupture on T6 in female: absent. Sculpture of T6 in female: smooth with fine setigerous punctures along lateral margin. Sculpture of S2: longitudinally striate anteromedially, otherwise smooth. Prominent longitudinal median carina on S2: absent.

##### Wings:

macropterous, apex or forewing extending beyond posterior margin of T3. Color of forewing in female: infuscate apical to R with white spot near apex of wing. Color of hind wing: hyaline throughout. Density of setation in fore wing: uniform throughout. Density of setation in hind wing: uniform throughout. Length of R1: more than 1.5 times as long as r. M+Cu and RS+M in forewing: nebulous.

**Figures 305–310.**
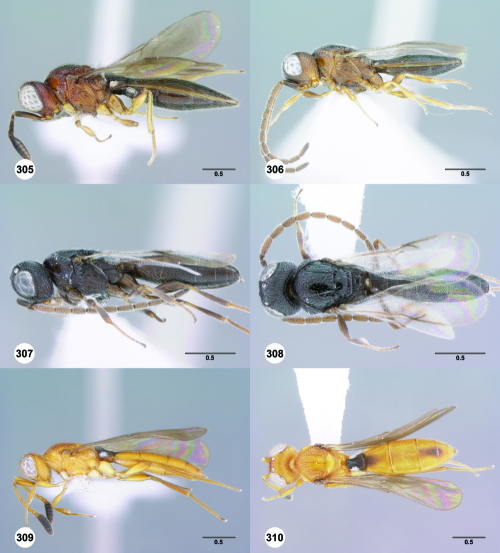
^118^ 305, Trichoteleia levii sp. n., Lateral habitus, female (CASENT 2043789) **306** Trichoteleia jiro sp. n., Lateral habitus, male (OSUC 143329) **307** Trichoteleia bicolor sp. n., Lateral habitus, male (CASENT 2135801) **308** Trichoteleia bicolor sp. n., Dorsal habitus, male (CASENT 2135801) **309** Trichoteleia hemlyae sp. n., Lateral habitus, female (CASENT 2136418) **310** Trichoteleia hemlyae sp. n., Dorsal habitus, female (CASENT 2136418). Scale bars in millimeters

#### Diagnosis.

Trichoteleia zuparkoi shares its pattern of wing coloration with Trichoteleia cincta, Trichoteleia delilah, and Trichoteleia picturata. The posterior half of the medial mesoscutum in Trichoteleia delilah and Trichoteleia picturata is longitudinally rugulose, and mostly smooth with moderately dense punctation in Trichoteleia zuparkoi. In Trichoteleia cincta, the frons below the median ocellus is dorsoventrally strigose and in Trichoteleia zuparkoi it is punctate rugulose.

#### Etymology.

This species is named for Dr. Robert Zuparko in gratitude for the tremendous amount of work involved to make the multitude of specimens available for this study.

#### Link to Distribution Map.

[http://hol.osu.edu/map-large.html?id=243937]

#### Material Examined.

Holotype, female: **MADAGASCAR:** Antsiranana Auto. Prov., #1938, Manongarivo Special Reserve, 13°59.9'S 48°25.7'E, 1175m, 20.X.1998, B. L. Fisher, OSUC 181061 (deposited in CASC). Other material: **MADAGASCAR:** 1 female, OSUC 266091 (CASC).

## Supplementary Material

XML Treatment for Trichoteleia

XML Treatment for Trichoteleia afo

XML Treatment for Trichoteleia albidipes

XML Treatment for Trichoteleia bicolor

XML Treatment for Trichoteleia bidentata

XML Treatment for Trichoteleia carinata

XML Treatment for Trichoteleia cincta

XML Treatment for Trichoteleia delilah

XML Treatment for Trichoteleia eburata

XML Treatment for Trichoteleia echinata

XML Treatment for Trichoteleia fisheri

XML Treatment for Trichoteleia funesta

XML Treatment for Trichoteleia halterata

XML Treatment for Trichoteleia hemlyae

XML Treatment for Trichoteleia irwini

XML Treatment for Trichoteleia janus

XML Treatment for Trichoteleia jiro

XML Treatment for Trichoteleia ketrona

XML Treatment for Trichoteleia levii

XML Treatment for Trichoteleia longiventris

XML Treatment for Trichoteleia minima

XML Treatment for Trichoteleia nify

XML Treatment for Trichoteleia oculea

XML Treatment for Trichoteleia orona

XML Treatment for Trichoteleia parvipennis

XML Treatment for Trichoteleia pauliani

XML Treatment for Trichoteleia picturata

XML Treatment for Trichoteleia prima

XML Treatment for Trichoteleia prolixa

XML Treatment for Trichoteleia quazii

XML Treatment for Trichoteleia ravaka

XML Treatment for Trichoteleia rugifrons

XML Treatment for Trichoteleia solocis

XML Treatment for Trichoteleia sphaerica

XML Treatment for Trichoteleia subtilis

XML Treatment for Trichoteleia tahotra

XML Treatment for Trichoteleia takariva

XML Treatment for Trichoteleia tezitra

XML Treatment for Trichoteleia tigris

XML Treatment for Trichoteleia tonsa

XML Treatment for Trichoteleia warreni

XML Treatment for Trichoteleia xantrox

XML Treatment for Trichoteleia zuparkoi

## Endnotes

1 http://biocol.org:urn:lsid:biocol.org:col:1011

2 http://biocol.org:urn:lsid:biocol.org:col:1012

3 http://biocol.org:urn:lsid:biocol.org:col:33864

4 http://biocol.org:urn:lsid:biocol.org:col:1014

5 http://biocol.org:urn:lsid:biocol.org:col:34411

6 http://biocol.org:urn:lsid:biocol.org:col:1019

7 http:// morphbank.net/?id=578543

8 http:// morphbank.net/?id=578544

9 http:// morphbank.net/?id=578545

10 http://morphbank.net/?id=578546

11 http://morphbank.net/?id=578547

12 http://morphbank.net/?id=578535

13 http://morphbank.net/?id=578548

14 http://morphbank.net/?id=578550

15 http://morphbank.net/?id=578549

16 http://morphbank.net/?id=578552

17 http://morphbank.net/?id=578551

18 http://morphbank.net/?id=578536

19 http://morphbank.net/?id=578582

20 http://morphbank.net/?id=578553

21 http://morphbank.net/?id=578554

22 http://morphbank.net/?id=578555

23 http://morphbank.net/?id=578556

24 http://morphbank.net/?id=578557

25 http://morphbank.net/?id=578558

26 http://morphbank.net/?id=578559

27 http://morphbank.net/?id=578560

28 http://morphbank.net/?id=578562

29 http://morphbank.net/?id=578561

30 http://morphbank.net/?id=578564

31 http://morphbank.net/?id=578565

32 http://morphbank.net/?id=578563

33 http://morphbank.net/?id=578566

34 http://morphbank.net/?id=578537

35 http://morphbank.net/?id=578567

36 http://morphbank.net/?id=578569

37 http://morphbank.net/?id=578568

38 http://morphbank.net/?id=578570

39 http://morphbank.net/?id=578572

40 http://morphbank.net/?id=578571

41 http://morphbank.net/?id=578574

42 http://morphbank.net/?id=578576

43 http://morphbank.net/?id=578575

44 http://morphbank.net/?id=578577

45 http://morphbank.net/?id=578573

46 http://morphbank.net/?id=578623

47 http://morphbank.net/?id=578579

48 http://morphbank.net/?id=578580

49 http://lsid.tdwg.org/urn:lsid:biosci.ohio-state.edu:osuc_pubs:131

50 http://lsid.tdwg.org/urn:lsid:biosci.ohio-state.edu:osuc_pubs:410

51 http://lsid.tdwg.org/urn:lsid:biosci.ohio-state.edu:osuc_pubs:334

52 http://lsid.tdwg.org/urn:lsid:biosci.ohio-state.edu:osuc_pubs:409

53 http://lsid.tdwg.org/urn:lsid:biosci.ohio-state.edu:osuc_pubs:344

54 http://lsid.tdwg.org/urn:lsid:biosci.ohio-state.edu:osuc_pubs:353

55 http://lsid.tdwg.org/urn:lsid:biosci.ohio-state.edu:osuc_pubs:229

56 http://lsid.tdwg.org/urn:lsid:biosci.ohio-state.edu:osuc_pubs:309

57 http://lsid.tdwg.org/urn:lsid:biosci.ohio-state.edu:osuc_pubs:387

58 http://lsid.tdwg.org/urn:lsid:biosci.ohio-state.edu:osuc_pubs:310

59 http://lsid.tdwg.org/urn:lsid:biosci.ohio-state.edu:osuc_pubs:7039

60 http://lsid.tdwg.org/urn:lsid:biosci.ohio-state.edu:osuc_pubs:342

61 http://lsid.tdwg.org/urn:lsid:biosci.ohio-state.edu:osuc_pubs:311

62 http://lsid.tdwg.org/urn:lsid:biosci.ohio-state.edu:osuc_pubs:474

63 http://lsid.tdwg.org/urn:lsid:biosci.ohio-state.edu:osuc_pubs:312

64 http://www.mapress.com/zootaxa/2007f/zt01571p078.pdf

65 http://lsid.tdwg.org/urn:lsid:biosci.ohio-state.edu:osuc_pubs:240

66 http://lsid.tdwg.org/urn:lsid:biosci.ohio-state.edu:osuc_pubs:20959

67 http://lsid.tdwg.org/urn:lsid:biosci.ohio-state.edu:osuc_pubs:405

68 http://morphbank.net/?id=578585

69 http://morphbank.net/?id=578627

70 http://morphbank.net/?id=578626

71 http://morphbank.net/?id=578628

72 http://morphbank.net/?id=578629

73 http://morphbank.net/?id=578630

74 http://morphbank.net/?id=578631

75 http://morphbank.net/?id=578632

76 http://morphbank.net/?id=578633

77 http://morphbank.net/?id=578583

78 http://morphbank.net/?id=578586

79 http://morphbank.net/?id=578587

80 http://morphbank.net/?id=578588

81 http://morphbank.net/?id=578589

82 http://morphbank.net/?id=578590

83 http://morphbank.net/?id=578591

84 http://morphbank.net/?id=578592

85 http://morphbank.net/?id=578593

86 http://morphbank.net/?id=578594

87 http://morphbank.net/?id=578540

88 http://morphbank.net/?id=578596

89 http://morphbank.net/?id=578597

90 http://morphbank.net/?id=578598

91 http://morphbank.net/?id=578599

92 http://morphbank.net/?id=578600

93 http://morphbank.net/?id=578601

94 http://morphbank.net/?id=578602

95 http://morphbank.net/?id=578603

96 http://morphbank.net/?id=578604

97 http://morphbank.net/?id=578605

98 http://morphbank.net/?id=578606

99 http://morphbank.net/?id=578607

100 http://morphbank.net/?id=578608

101 http://morphbank.net/?id=578609

102 http://morphbank.net/?id=578610

103 http://morphbank.net/?id=578611

104 http://morphbank.net/?id=578612

105 http://morphbank.net/?id=578613

106 http://morphbank.net/?id=578614

107 http://morphbank.net/?id=578615

108 http://morphbank.net/?id=578616

109 http://morphbank.net/?id=578617

110 http://morphbank.net/?id=578618

111 http://morphbank.net/?id=578619

112 http://morphbank.net/?id=578620

113 http://morphbank.net/?id=578621

114 http://morphbank.net/?id=578622

115 http://morphbank.net/?id=578578

116 http://morphbank.net/?id=578624

117 http://morphbank.net/?id=578625

118 http://morphbank.net/?id=578634

119 http://morphbank.net/?id=578595

120 http://www.gbif.org/informatics/standards-and-tools/publishing-data/data-standards/darwin-core-archives/

121 http://rs.tdwg.org/dwc/terms/guides/text/index.htm

119 http://code.google.com/p/gbif-providertoolkit/

## Supplementary Material

Appendix I. Occurrence data (doi: 10.3897/zookeys.80.907.app.1a) and taxon checklist (doi: 10.3897/zookeys.80.907.app.1b) for all records used in the present paper. File format: DarwinCore Archive. **Explanation note:** Darwin Core Archive^120^ (DwC-A) is an international biodiversity informatics data standard and the preferred format for publishing data to the Global Biodiversity Information Facility (GBIF) network. Each Darwin Core Archive consists of at least three files: (1) one or more data files keeping all records of the particular dataset in a tabular format such as a comma-separated or tab-separated list; (2) the archive descriptor (meta.xml) file describing the individual data file columns used and mapping them to DwC terms; and (3) a metadata file describing the entire dataset which GBIF recommends to be EML (Ecological Metadata Language) 2.1.1 based. The format is defined in the Darwin Core text guidelines^121^. Darwin Core is no longer strictly bound to occurrence data, and together with Dublin Core (on which its ideas are based), it is used by GBIF and others to encode data about organism names, taxonomies and species information. The Darwin Core Archive is available as supplementary file to the present revision and can be also simultaneously published via the GBIF infrastructure through the Integrated Publishing Toolkit^122^ (IPT).

## References

[B1] Bin F (1981) Definition of female antennal clava based on its plate sensilla in Hymenoptera Scelionidae Telenominae. Redia 64: 245–261 ^49^

[B2] Dodd A (1914) Further new genera and species of Australian Proctotrypoidea. Proceedings of the Royal Society of Queensland 26: 91–140 ^50^

[B3] Dodd A (1915) Notes and corrections on Australian Prototrypoidea, with descriptions of forty-five new species. Archiv fur Naturgeschichte 80(9): 1–32 ^51^

[B4] Dodd A (1920) Notes on the exotic Proctotrupoidea in the British and Oxford University Museums, with descriptions of new genera and species. Transactions of the Entomological Society of London 1919: 321–382 ^52^

[B5] Dodd A (1929) A revision of four genera of Australian Scelionidae. Proceedings of the Royal Society of Queensland 40: 30–50 ^53^

[B6] Galloway ID (1976) The types of Australian species of the subfamily Scelioninae (Hymenoptera: Scelionidae). Queensland Journal of Agricultural and Animal Sciences 33(1): 83–114 ^54^

[B7] Johnson NF (1992) Catalog of world Proctotrupoidea excluding Platygastridae. Memoirs of the American Entomological Institute 51: 1–825 ^55^

[B8] Kieffer JJ (1910) Hymenoptera. Fam. Scelionidae. Addenda et corrigenda. Genera Insectorum 80: 61–112 ^56^

[B9] Kieffer JJ (1912) Hymenoptera, Proctotrupoidea. Transactions of the Linnean Society of London, Zoology 15: 45–80 ^57^

[B10] Kieffer JJ (1926) Scelionidae. Das Tierreich. Vol. 48 Walter de Gruyter & Co., Berlin, 885 pp. ^58^

[B11] Mason WRM (1986) Standard drawing conventions and definitions for venational and other features of wings of Hymenoptera. Proc. Entomol. Soc. Wash. 88: 1–7 ^59^

[B12] Masner L (1965) The types of Proctotrupoidea (Hymenoptera) in the British Museum (Natural History) and in the Hope Department of Entomology, Oxford. Bulletin of the British Museum (Natural History) Entomology Supplement 1: 1–154 ^60^

[B13] Masner L (1976) Revisionary notes and keys to world genera of Scelionidae (Hymenoptera: Proctotrupoidea). Memoirs of the Entomological Society of Canada 97: 1–87 ^61^

[B14] Masner L (1980) Key to genera of Scelionidae of the Holarctic region, with descriptions of new genera and species (Hymenoptera: Proctotrupoidea). Memoirs of the Entomological Society of Canada 113: 1–54 ^62^

[B15] Masner L Muesebeck CFW (1968) The types of Proctotrupoidea (Hymenoptera) in the United States National Museum. Bulletin of the United States National Museum 270: 1–143 ^63^

[B16] Mikó I Vilhelmsen L Johnson NF Masner L Pénzes Z (2007) Skeletomusculature of Scelionidae (Hymenoptera: Platygastroidea): head and mesosoma. Zootaxa 1571: 1–78 ^64^

[B17] Muesebeck CFW Walkley LM (1956) Type species of the genera and subgenera of parasitic wasps comprising the superfamily Proctotrupoidea (order Hymenoptera). Proceedings of the U.S. National Museum 105: 319–419 ^65^

[B18] Polaszek A Agosti D Alonso-Zarazaga M Beccaloni G de Place Bjørn P Bouchet P Brothers DJ Earl of Cranbrook Evenhuis NL Godfray HCJ Johnson NF Krell FT Lipscomb D Lyal CHC Mace GM Mawatari SF Miller SE Minelli A Morris S Ng PKL Patterson DJ Pyle RL Robinson N Rogo L Taverne J Thompson FC van Tol J Wheeler QD Wilson EO (2005) A universal register for animal names. Nature 437: 477 ^66^10.1038/437477a 16177765

[B19] Risbec J (1956) Proctotrupides Scelionini de Madagascar [Hymenopteres]. Revue Française d’Entomologie 23: 244–264 ^67^

